# Association of Disease-Modifying Antirheumatic Drugs (DMARDs) with Cardiovascular Diseases: Evidence from a Drug Target Mendelian Randomization Study

**DOI:** 10.5334/gh.1526

**Published:** 2026-02-26

**Authors:** Chengui Zhuo, Lei Chen, Xiangjie Sun, Ting Chen, Haipeng Cai, Xiaosheng Hu

**Affiliations:** 1 Department of Cardiology, The First Affiliated Hospital, Zhejiang University School of Medicine, Hangzhou, Zhejiang, China; 2 Department of Cardiology, Taizhou Central Hospital (Taizhou University Hospital), Taizhou, Zhejiang, China

**Keywords:** DMARD target genes, cardiovascular diseases, colocalization analyses, summary-data-based MR, two-sample Mendelian randomization

## Abstract

**Objectives:**

Cardiovascular diseases (CVDs) still represent a major cause of mortality, with inflammation playing a key role in their pathogenesis. Thus, elucidating the possible effects of disease-modifying antirheumatic drugs (DMARDs) on CVD risk in the general population may hold considerable clinical implications.

**Methods:**

Genetic instruments were employed to proxy the pharmacological effects of seven DMARD classes, including sulfasalazine, cyclosporine, leflunomide, IL-6 inhibitors, TNF-alpha inhibitors, abatacept, rituximab, and JAK inhibitors. To investigate their potential causal associations with 11 CVD outcomes, a comprehensive framework incorporating two-sample Mendelian randomization (TSMR), summary-data-based MR (SMR), and colocalization analysis was developed. Lastly, several sensitivity analyses were undertaken to verify the robustness of our findings.

**Results:**

In the primary TSMR results, sulfasalazine targeting PLA2G1B was linked to reduced risks of heart failure (OR: 0.86, 95% CI: 0.80–0.94), total cholesterol (OR: 0.89, 95% CI: 0.83–0.95), high-density lipoprotein cholesterol (OR: 0.88, 95% CI: 0.82–0.94), and aortic stenosis (OR: 0.72, 95% CI: 0.62–0.84). Sulfasalazine targeting RELB exhibited similar protective associations, whereas RELA exhibited the opposite associations. Moreover, IL-6R was robustly associated with increased risks of atrial fibrillation (OR: 1.29, 95% CI: 1.16–1.44), coronary artery disease (OR: 1.38, 95% CI: 1.23–1.56), myocardial infarction (OR: 1.27, 95% CI: 1.11–1.44), ischemic stroke (OR: 1.34, 95% CI: 1.22–1.48), and aortic stenosis (OR: 1.75, 95% CI: 1.46–2.09). Genetically higher IL-6R expression was associated with increased CVD risk, suggesting that IL-6 inhibition may confer cardiovascular benefit. SMR analysis further validated the associations of RELA, CD80, and IL-6R with one or more cardiovascular phenotypes. Finally, colocalization analyses for IL-6R and RELB provided strong evidence supporting their involvement in multiple CVDs.

**Conclusion:**

Overall, this study presents evidence supporting a causal association between DMARDs and several CVDs. Nevertheless, further clinical investigations are necessary to validate our findings.

## Introduction

As is well documented, cardiovascular diseases (CVDs) are a primary factor contributing to mortality and disability worldwide, comprising approximately 30% of global mortality and imposing a heavy load on healthcare systems (1). This burden highlights the necessity of investigating more efficient preventive and therapeutic approaches. Notably, rheumatoid arthritis (RA), a systemic autoimmune disorder defined by long-standing and progressive inflammation, has been associated with a two-fold increased risk of CVDs, comparable to that observed in type 2 diabetes mellitus (2,3). This association emphasizes the potential of disease-modifying antirheumatic drugs (DMARDs) in preventing CVDs.

Given that inflammation performs a central role in the development of CVDs, DMARDs have been hypothesized to improve cardiovascular outcomes (4). However, emerging evidence suggests that the impact of DMARDs on CVD risk remains controversial (3,5). In a systematic review that involved four studies evaluating the impact of methotrexate on myocardial infarction (MI), one study demonstrated a statistically significant decline in MI risk, whereas the remaining three studies only indicated a trend toward risk reduction (5). Tumor Necrosis Factor Alpha (TNF-alpha) inhibitors have been established to improve the prognosis of CVDs (6,7). Nevertheless, the majority of related studies are limited to RA populations, and their cardiovascular benefits in the general population remain elusive. A randomized controlled trial (RCT) has reported that a single 280 mg infusion of tocilizumab (interleukin-6 (IL-6) inhibitor) can enhance myocardial salvage in patients with acute MI. However, this study encompassed only a six-month follow-up duration, and the long-term prognostic effect of tocilizumab, as well as its impacts on other CVDs, remain unknown (8).

Considering the complexities of CVDs and the pleiotropic effects of DMARDs, elucidating the therapeutic potential of these agents in CVDs is critical and demands robust methodologies. Mendelian randomization (MR) is a powerful method employed to analyze causal associations between exposures and outcomes (9). By utilizing genetic variants that mimic the pharmacological actions of drug targets, MR studies offer an effective and robust alternative to RCTs for exploring the disease-preventive potential of drugs (10). Therefore, in the present study, a drug target MR analysis was employed to assess the association between DMARDs and CVDs, including coronary artery disease (CAD), MI, lipid profile, heart failure (HF), atrial fibrillation (AF), hypertension (HTN), ischemic stroke (IS), and aortic stenosis (AS).

## Method

### Study design

**Figure 1:**
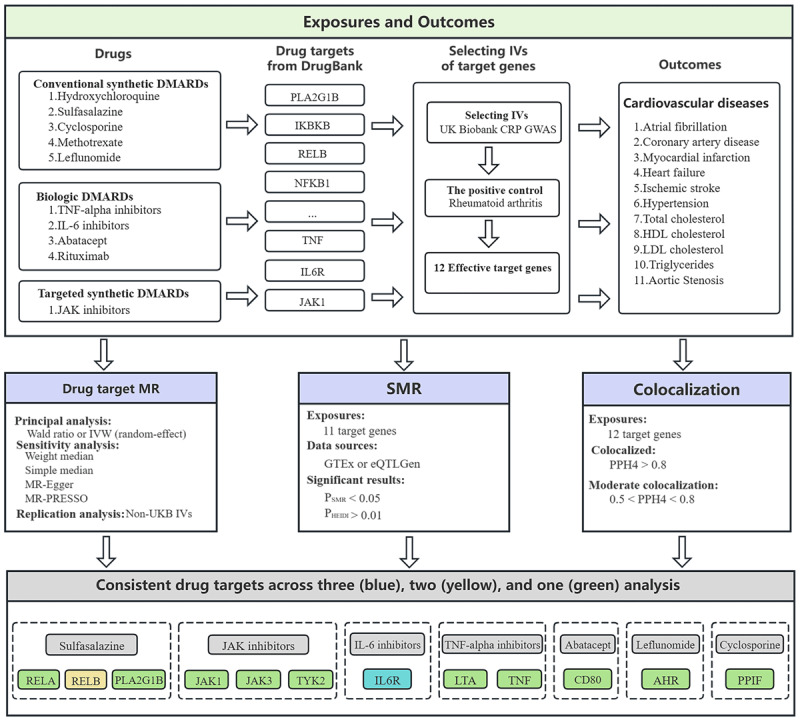
Flowchart depicting the systematic framework used to investigate the associations between DMARD target genes and cardiovascular diseases. Abbreviations: IVs, instrumental variables; CRP, C-reactive protein; GWAS, genome-wide association studies; HDL cholesterol, high-density lipoprotein cholesterol; LDL cholesterol, low-density lipoprotein cholesterol; MR, Mendelian randomization; IVW, inverse-variance weighted; SMR, summary-based Mendelian randomization.

Within this study, Mendelian randomization and colocalization analyses were performed using publicly accessible summary statistics from genome-wide association studies (GWASs) and expression quantitative trait loci (eQTL) studies (Table S1). All included studies were authorized by their relevant institutional review boards. The flowchart of this study is displayed in Figure 1.

### Genetic instruments for DMARDs

The selection of genetic instruments for DMARDs adopted the following steps. Firstly, three types of DMARD (Table S2) were identified as exposures using the DrugBank database (https://go.drugbank.com/), and their target gene information is listed in Table S3. Secondly, genetic instruments were proposed by selecting genome-wide significant (P < 5E-8) single-nucleotide polymorphisms (SNPs) within the cis-region (±500kb) of each drug’s targets, using summary statistics extracted from the UK Biobank GWAS on serum C-reactive protein (CRP) levels (11,12). SNPs were clumped using a threshold of r^2^ < 0.3 (13). Where SNPs were absent in the outcome GWASs, proxy SNPs (r^2^ > 0.8) were identified. This approach resulted in the identification of several genetic instruments for the target genes of eight drugs: sulfasalazine, cyclosporine, leflunomide, abatacept, rituximab, IL-6 inhibitors, TNF-alpha inhibitors, and Janus-associated kinase (JAK) inhibitors (Table S4). Hydroxychloroquine and methotrexate were not included in further analyses due to insufficient genetic instruments. As the target genes TNF and LTA shared identical SNPs, they were combined as ‘TNF/LTA’. Finally, two GWASs on RA were applied as positive controls to verify the effectiveness of the selected target genes. In the replication analysis, instruments from other GWAS databases on CRP levels were also selected. Details of all datasets used are summarized in Table S1. Additionally, to validate the observed association using SNPs selected from GWASs, instruments for each target gene were identified from the cis-eQTLs located within 1 Mb of the target genes. Considering the potential complexity of multi-target DMARDs, we additionally performed MR analyses using cis-eQTL-defined SNPs.

### Outcome sources

The characteristics of all CVD outcomes are outlined in Table S1. The proportion of overlapping samples between the exposure and each outcome GWAS ranged from approximately 0% to 40.33% (Table S5). To evaluate potential sample overlap biases, replication analyses were conducted using other exposure GWAS datasets.

### Statistical analyses

#### Primary analysis

A two-sample MR (TSMR) analysis was utilized to assess the causal effects of target genes on positive controls and CVDs. The random-effects inverse-variance weighting (IVW) method was employed as the primary analysis, supplemented by the Wald ratio approach for instruments with only a single SNP. Considering the divergent pharmacological effects of DMARDs (Table S3), the overall effect of each DMARD on cardiovascular outcomes was estimated by aggregating target-specific effects according to their pharmacological direction of action. Using summary-level eQTL data as instruments, summary-data-based Mendelian randomization (SMR) analysis was performed to investigate the association between the expression of target genes and the risk of CVDs using the SMR software with default settings.

Additionally, a Bayesian colocalization approach was implemented to explore the degree to which the expression of target genes and CVDs is influenced by the same causal genetic variant. This approach generated posterior probabilities for five hypotheses (PPH): H0 denotes the variant showing no association with either trait; H1 reflects an association exclusively with trait 1; H2 denotes a relationship solely with trait 2; H3 proposes that each trait is associated with different variants; and H4 indicates that both traits share an association with the same causal variant (14). A colocalization approach was applied via the ‘coloc’ R package (version 5.2.3) with default settings (p1 = 1 × 10^–04^, p2 = 1 × 10^–04^, p12 = 1 × 10^–05^), reflecting the assumed probabilities of association with each trait and their joint association. A PPH(H4) ≥ 0.9 was interpreted as strong colocalization between target genes and CVD outcomes, whereas 0.8 ≤ PPH(H4) < 0.9 indicated moderate colocalization, and 0.5 ≤ PPH(H4) < 0.8 indicated weak colocalization (Figure 1).

#### Sensitivity analysis

To enhance the robustness of the results, several sensitivity analyses were applied. Firstly, the F-statistic was computed to evaluate the strength of the chosen SNPs via the formula \[
F~=~\frac{{{R}^{2}}\left(N~-~2\right)}{\left(1~-~{{R}^{2}}\right)}\] , where R^2^ denotes the proportion of the phenotypic variation attributable to SNPs and N stands for the GWAS sample size. To reduce the possibility of weak instrument bias, SNPs with an F-statistic exceeding 10 were retained (15). Secondly, heterogeneity was assessed using the I^2^ and Cochran’s Q statistic, and heterogeneity was considered present if I^2^ exceeded 25% or the P-value from Cochran’s Q test was below 0.05. In this study, the random-effects IVW approach was employed to address potential bias arising from heterogeneity (16). Thirdly, to evaluate the existence of horizontal pleiotropy, MR-Egger regression and the Mendelian Randomization Pleiotropy RESidual Sum and Outlier (MR-PRESSO) method were implemented. In MR-Egger regression, an intercept-derived P-value < 0.05 suggests the presence of pleiotropy (17). The MR-PRESSO method, on the other hand, was applied to identify outliers potentially driven by horizontal pleiotropic effects and generate corrected causal estimates, with a Global Test P-value < 0.05 suggesting the existence of outliers (18). Fourthly, leave-one-out analyses were used to evaluate whether the observed association was affected by individual SNPs. Additionally, other TSMR methods, including weighted median, simple median, IVW (fixed-effects), and IVW (multiplicative random effects), were adopted to ensure the robustness of causal estimation. All TSMR analyses were performed via the ‘TwoSampleMR’ R package (version 0.6.15) with default settings. Summary statistics from exposure and outcome GWAS datasets were harmonized using harmonise_data() in ‘TwoSampleMR’, ensuring alleles were strand-aligned and consistently oriented across datasets.

For the SMR approach, the heterogeneity in dependent instruments (HEIDI) test was applied using the SMR tool to evaluate the possibility that the identified association between gene expression and outcome could be attributed to linkage, which may fail to satisfy the fundamental assumptions of MR. A HEIDI test P-value of less than 0.01 was deemed to indicate a linkage-driven association rather than one mediated by gene expression regulation (19).

Statistical significance was established as a two-sided P-value of less than 0.05. To control for multiple comparisons, a false discovery rate (FDR)-adjusted significance threshold of q < 0.05 (Benjamini–Hochberg method) was adopted as the primary inferential framework. Additionally, a Bonferroni-adjusted significance threshold of P-values < 4.13 × 10^–4^ was further applied as a conservative safeguard. All statistical analyses were conducted using R software (version 4.3.0) and the SMR software (version 1.3.1) on the Windows platform.

## Results

### Effective DMARD target genes and selection of genetic instruments

Nineteen drug targets across DMARDs were identified that could be harmonized with CVD outcomes: sulfasalazine (PLA2G1B, REL, RELA, RELB, ABCG2, NFKB1), cyclosporine (PPP3R2, PPIF), leflunomide (AHR), TNF-alpha inhibitors (TNF, LTA, FCGR1 A, C1QA/C1QB/C1QC), IL-6 inhibitors (IL-6R), abatacept (CD80), rituximab (MS4 A1), and JAK inhibitors (JAK1, JAK3, TYK2). A total of 11 target genes (PLA2G1B, RELA, RELB, PPIF, AHR, TNF/LTA, IL-6R, CD80, JAK1, JAK3, TYK2) were significantly causally associated with positive controls and thus selected for further analysis (Table S6). The remaining targets were excluded (REL, ABCG2, NFKB1, PPP3R2, FCGR1 A, MS4 A1, C1QA/C1QB/C1QC), with details shown in Table S6. Finally, a total of 271 independent SNPs were identified as instruments for DMARDs, and the F-statistics for each of the selected genetic instruments were higher than 24 (Table S4).

### TSMR analysis

The primary TSMR analysis revealed that all selected target genes demonstrated potential causal associations with at least one CVD phenotype. To avoid over-interpretation of drug efficacy, Table S7 provides a structured framework summarizing drug–target action directions, biological pathways, and integrated drug-level TSMR associations. Under the primary FDR correction, significant associations were observed for PLA2G1B with CAD, HF, IS, TC, HDL-C, LDL-C, and AS; RELB with CAD, MI, HF, TC, HDL-C, LDL-C, and TG; RELA with AF, CAD, MI, HF, and HTN; IL-6R with AF, CAD, MI, IS, HDL-C, and AS; and additional signals (Table S8). In addition, following Bonferroni correction for multiple testing (P < 0.05/121 [4.13 × 10^–4^]), statistically significant associations remained for PLA2G1B, RELA, and RELB (targets of sulfasalazine); CD80 (target of abatacept); TNF/LTA (target of TNF-alpha inhibitors); IL-6R (target of IL-6 inhibitors); and JAK3 (target of JAK inhibitors), each with at least one CVD phenotype.

**Figure 2:**
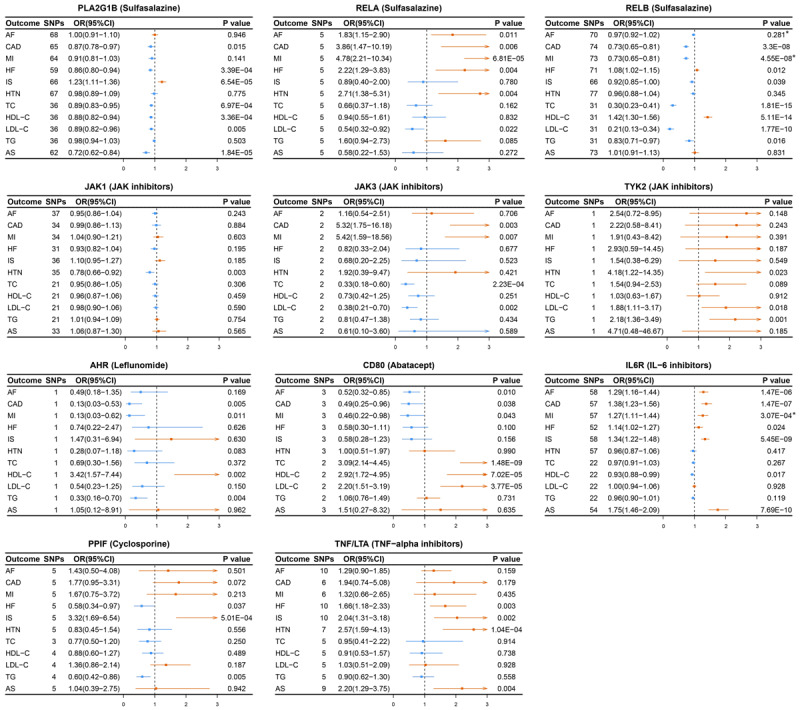
Forest plots showing the causal effects of the 12 DMARD target genes on cardiovascular diseases, with blue indicating odds ratios (OR) < 1 (protective effects) and orange denoting OR > 1 (harmful effects). * indicates P < 0.05 in the MR-Egger intercept test. Abbreviations: AF, atrial fibrillation; CAD, coronary artery disease; MI, myocardial infarction; HF, heart failure; IS, ischemic stroke; HTN, hypertension; TC, total cholesterol; HDL-C, high-density lipoprotein cholesterol; LDL-C, low-density lipoprotein cholesterol; TG, triglycerides; AS, aortic stenosis.

As illustrated in Figure 2, the random-effects IVW approach suggested that genetic proxies for PLA2G1B inhibition (mimicking sulfasalazine action) were associated with a decreased risk of multiple CVD phenotypes, including CAD (OR: 0.87, 95% CI: 0.78–0.97, P = 0.015), HF (OR: 0.86, 95% CI: 0.80–0.94, P = 3.39 × 10^–4^), TC (OR: 0.89, 95% CI: 0.83–0.95, P = 6.97 × 10^–4^), HDL-C (OR: 0.88, 95% CI: 0.82–0.94, P = 3.36 × 10^–4^), LDL-C (OR: 0.89, 95% CI: 0.82–0.96, P = 0.005), and AS (OR: 0.72, 95% CI: 0.62–0.84, P = 1.84 × 10^–5^). Following correction for multiple testing, the associations with HF, HDL-C, and AS remained statistically significant. Similarly, sulfasalazine targeting RELB demonstrated consistent protective associations with CAD (OR: 0.73, 95% CI: 0.65–0.81, P = 3.30 × 10^–8^), MI (OR: 0.73, 95% CI: 0.65–0.81, P = 4.55 × 10^–8^), TC (OR: 0.30, 95% CI: 0.23–0.41, P = 1.81 × 10^–15^), HDL-C (OR: 1.42, 95% CI: 1.30–1.56, P = 5.11 × 10^–14^), and LDL-C (OR: 0.21, 95% CI: 0.13–0.34, P = 1.77 × 10^–10^). In contrast, targeting RELA showed the opposite trend, with evidence suggestive of increased risks of CAD and MI. Given the antagonistic effect of sulfasalazine on PLA2G1B and its activating effect on RELA and RELB (Table S3), these targets were combined based on pharmacological action to estimate the overall effect of sulfasalazine. The combined target analysis (Figure S1) unveiled significant associations with the risks of HF (OR: 1.11, 95% CI: 1.06–1.17, P = 1.68 × 10^–5^), IS (OR: 0.88, 95% CI: 0.83–0.94, P = 6.02 × 10^–5^), TC (OR: 0.54, 95% CI: 0.44–0.67, P = 9.98 × 10^–9^), HDL-C (OR: 1.28, 95% CI: 1.20–1.36, P = 4.32 × 10^–15^), and LDL-C (OR: 0.45, 95% CI: 0.33–0.61, P = 1.65 × 10^–7^).

Moreover, IL-6R, targeted by IL-6 inhibitors, was significantly associated with increased risks of AF (OR: 1.29, 95% CI: 1.16–1.44, P = 1.47 × 10^–6^), CAD (OR: 1.38, 95% CI: 1.23–1.56, P = 1.47 × 10^–7^), MI (OR: 1.27, 95% CI: 1.11–1.44, P = 3.07 × 10^–4^), IS (OR: 1.34, 95% CI: 1.22–1.48, P = 5.45 × 10^–9^), and AS (OR: 1.75, 95% CI: 1.46–2.09, P = 7.69 × 10^–10^). Accordingly, IL-6 inhibitors may exert potential protective effects against these cardiovascular outcomes, as shown in Figure S1. Meanwhile, TNF/LTA, the target of TNF-alpha inhibitors, was linked to an increased risk of HTN (OR: 2.57, 95% CI: 1.59–4.13, P = 1.04 × 10^–4^). JAK3, targeted by JAK inhibitors, showed a significant inverse association with TC levels (OR: 0.33, 95% CI: 0.18–0.60, P = 2.23 × 10^–4^), whereas CD80, targeted by abatacept, exhibited a positive association (OR: 3.09, 95% CI: 2.14–4.45, P = 1.48 × 10^–9^). Although JAK1 and TYK2 (targets of JAK inhibitors), AHR (target of leflunomide), and PPIF (target of cyclosporine) showed associations with at least one CVD phenotype, none remained significant after Bonferroni-adjusted multiple testing correction (Figure 2). Considering the divergent pharmacological effects of DMARDs, the overall estimated causal effect of IL-6R inhibitors and other DMARDs on CVD risk is summarized in Figure S1.

Sensitivity analyses, employing the weighted median, simple median, MR-Egger, fixed-effects IVW, and multiplicative random-effects IVW methods, yielded results largely consistent with the primary TSMR findings (Table S9). For quality control, the MR-Egger intercept test revealed indications of pleiotropy in only a limited number of target–outcome pairs (Figure 2; Table S10). Additionally, the MR-PRESSO analysis detected outliers; however, their exclusion did not materially alter the results, indicating modest bias from horizontal pleiotropy (Table S11). In replication analyses based on other GWAS datasets, associations involving PLA2G1B, RELA, RELB, IL-6R, and TNF/LTA were replicated at least once (Figures S2–3), thereby attenuating the potential for bias caused by sample overlap. For key drug target signals, including IL-6R, PLA2G1B, RELA, and RELB, subsequent MR-Egger intercept and MR-PRESSO analyses demonstrated that potential pleiotropy did not generally significantly modify either the direction or the magnitude of the causal estimates. The only exception to this was the association between RELB and LDL-C, where MR-PRESSO showed evidence that pleiotropy materially affected the effect size and should be interpreted with caution. The leave-one-out analyses (Figures S4–S13) showed that the causal estimates for most associations between key drug targets and cardiovascular outcomes were generally robust. However, the analyses also identified potentially influential SNPs that may drive the estimates for several specific associations, including RELA with CAD and LDL-C, RELB with IS and TG, PLA2G1B with LDL-C, and IL-6R with HF. Consequently, these specific findings warrant careful interpretation.

The cis-eQTL-based MR analyses yielded effect estimates that were generally directionally consistent, which supported the robustness of our main findings (Figure S14). At the same time, the comparison between the two instrument sources revealed several outcome-specific discrepancies in effect direction. For genetic proxies of IL-6 inhibitors, differences were observed for HF and HDL-C, whereas for sulfasalazine-related targets, directional inconsistencies were noted for AF, IS, HDL-C, and AS.

### SMR analysis

**Figure 3:**
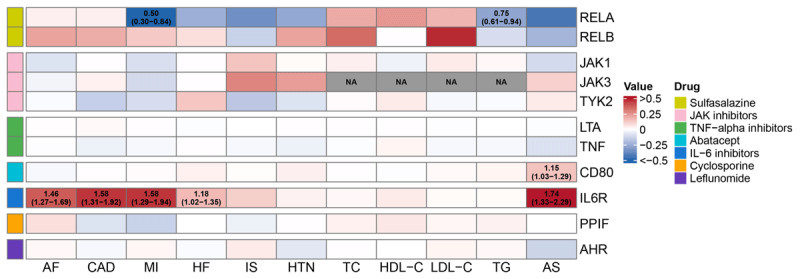
Gene expression analysis of DMARD target genes in relation to cardiovascular diseases. Positive associations are shown in red, negative associations in blue, and ORs with 95% CIs are presented for results that are statistically significant and meet the HEIDI test criteria.

For the SMR method, the gene expression levels of RELA, CD80, and IL-6R were significantly associated with one or more cardiovascular phenotype and passed the HEIDI test (Figure 3), implying that the associations were unlikely to be confounded by linkage. Specifically, increased expression of IL-6R was associated with an increased risk of AF (OR: 1.46, 95% CI: 1.27–1.69, P = 2.41 × 10^–7)^, CAD (OR: 1.58, 95% CI: 1.31–1.92, P = 2.17 × 10^–6)^, MI (OR: 1.58, 95% CI: 1.29–1.94, P = 1.10 × 10^–5^), HF (OR: 1.18, 95% CI: 1.02–1.35, P = 0.022), and AS (OR: 1.74, 95% CI: 1.33–2.29, P = 6.05 × 10^–5^). Similarly, higher CD80 expression, targeted by abatacept, was associated with an elevated risk of AS (OR: 1.15, 95% CI: 1.03–1.29, P = 0.013). Conversely, RELA expression, targeted by sulfasalazine, was linked to a decreased risk of MI (OR: 0.50, 95% CI: 0.30–0.84, P = 0.009) and TG (OR: 0.75, 95% CI: 0.61–0.94, P = 0.013).

### Colocalization analysis

**Figure 4:**
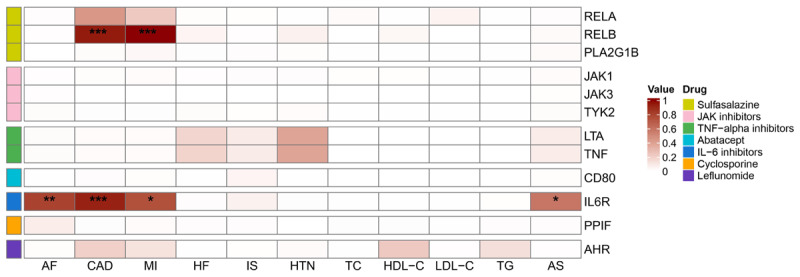
A colocalization analysis of DMARD target gene expression with cardiovascular diseases. ***Indicates strong evidence of colocalization (PPH4 ≥ 0.9), while **denotes moderate evidence (0.8 ≤ PPH4 < 0.9), and *denotes weak evidence (0.5 ≤ PPH4 < 0.8).

Colocalization analysis was performed to further verify the robustness of the observed associations between DMARD targets and CVDs. For IL-6R, strong colocalization was identified exclusively for CAD (PPH4 = 0.93). Evidence for AF was classified as moderate (PPH4 = 0.80), whereas associations with MI (PPH4 = 0.75) and AS (PPH4 = 0.58) were supported by comparatively weak colocalization evidence. Consistently, RELB demonstrated strong colocalization with both CAD (PPH4 = 0.95) and MI (PPH4 = 1.00). Notably, IL-6R demonstrated consistent associations with CVDs across TSMR, SMR, and colocalization analyses, supporting its potential causal role. Full details of the colocalization analysis are provided in Figure 4 and Figure S15.

## Discussion

The present comprehensive MR analysis provides evidence supporting a potential causal role of DMARDs in the development of CVDs. These findings should be interpreted with caution, as genetic findings primarily predict the directionality of pharmacologic action rather than the therapeutic magnitude achievable in clinical practice. Notably, IL-6 inhibitors and sulfasalazine (PLA2G1B, RELA, and RELB) demonstrated potential protective effects against CAD, MI, and IS. At the same time, IL-6 inhibitors were associated with a reduced risk of AF, HF, and AS. In contrast, sulfasalazine was linked to an increased risk of HF and AS, highlighting the complexity of its cardiovascular effects. Several other DMARDs, including JAK inhibitors, abatacept, cyclosporine, leflunomide, and TNF-alpha inhibitors, were also associated with one or more CVD phenotypes. Collectively, these findings extend beyond previous studies and offer new insights into the potential impact of DMARDs in modulating the progression of CVDs.

While earlier investigations have examined the effect of IL-6 inhibitors on the progression of CAD and MI in patients with RA, evidence in the general population remains limited (20,21,22). This study extends these observations by presenting genetic evidence supporting a causal association between IL-6 inhibitors and reduced risk of these cardiovascular outcomes in the general population. It is important to note that the genetic instrument for IL-6R reflects increased IL-6R expression and enhanced IL-6 signaling, which was associated with higher CVD risk. In contrast, IL-6R inhibitors suppress this pathway, producing the opposite biological effect. Thus, the detrimental associations observed for genetically elevated IL-6R expression are consistent with the potential protective effects of pharmacologic IL-6R inhibitors.

Interestingly, IL-6 inhibitors were found to exert protective effects against AS, an association that, to our knowledge, has not been documented in prior research. This finding was consistently supported across multiple analytical approaches, including TSMR, SMR, and colocalization analyses, highlighting the robustness and reliability of this finding. Mechanistically, IL-6 inhibitors such as tocilizumab bind to soluble IL-6R and membrane-bound IL-6R, thereby blocking IL-6-mediated signaling. In turn, this blockade suppresses the activation of T and B lymphocytes and attenuates pro-inflammatory pathways (23). Given these immunomodulatory effects, and considering that inflammatory processes are critical to all phases of atherosclerosis (24,25,26), IL-6 inhibition may exert cardioprotective effects. Additionally, IL-6 inhibitors have been reported to enhance endothelial function (27,28), further supporting their potential benefits in atherosclerotic disease.

Similarly, inflammation also makes a key contribution to the pathogenesis of AF (29). However, it is worth emphasizing that broad-spectrum anti-inflammatory therapies have failed to demonstrate efficacy in preventing AF (30). Herein, IL-6 inhibitors exerted a consistent protective effect on AF risk, which is consistent with the observations of Shitole et al., who reported that elevated levels of IL-6, high-sensitivity CRP, leukocyte count, TNF receptor 1, and IL-2 receptor alpha were positively associated with incident AF in the elderly population. However, after adjusting for these inflammatory biological markers, only IL-6 continued to be significantly linked to AF, emphasizing its pivotal function in the onset and progression of AF (31).

The causal association between sulfasalazine targets (PLA2G1B, RELA, and RELB) and CVD outcome, as identified through the TSMR analyses herein, suggests that sulfasalazine may increase the risk of HF, a finding not previously reported in prior studies. RELA, a critical constituent of the NF-κB transcription factor family, modulates the expression of multiple pro-inflammatory cytokines through the canonical NF-κB signaling pathway (32). More importantly, it has been shown to strongly contribute to the pathophysiology of myocardial fibrosis and cardiac dysfunction via sustained NF-κB activation (33). On the other hand, evidence for the role of PLA2G1B in HF remains scarce, although the results of TSMR suggested a potential inverse association between PLA2G1B expression and HF risk. Given that sulfasalazine acts as an antagonist of PLA2G1B while activating RELA and RELB, combined target analysis indicated a significant increase in HF risk. These findings highlight the need for caution when prescribing sulfasalazine to patients with, or at high risk of, HF, and the necessity for long-term clinical studies to evaluate its cardiovascular safety profile.

In our TSMR analyses, sulfasalazine targets (PLA2G1B, RELA, and RELB) showed opposing associations across some outcomes (e.g., CAD, HF, and IS), highlighting heterogeneity in target-level effects rather than a uniform drug effect. RELA primarily drives the canonical NF-κB pathway (32), whereas RELB is more closely linked to the non-canonical axis, which may yield distinct transcriptional outputs across cardiovascular phenotypes. Furthermore, Navarro et al. reported that RELB suppresses inflammatory gene expression in dendritic cells by competing with RELA for binding to NF-κB sites in target gene promoters, providing a plausible mechanistic basis for the opposite directions of associations observed for RELA and RELB (34). In addition, PLA2G1B, RELA, and RELB may influence cardiovascular outcomes not only through inflammation but also via pleiotropic pathways such as lipid metabolism, thrombotic pathways, and microvascular remodeling (35,36). Therefore, CRP-based instruments may capture only part of these drug targets’ cardiovascular effects and may provide limited estimates of CRP-independent pathways.

Although the SMR and colocalization analyses did not validate these findings, the results of TSMR suggest that several other DMARDs may also be linked to at least one CVD phenotype. Growing evidence indicates that immune dysregulation is a key contributor to the pathogenesis of hypertension (37). Previous studies have reported that targeted biologic therapies, such as TNF-alpha inhibitors, may reduce blood pressure in patients with RA, although these effects have not been consistently reproduced (38). Consistent with this, our findings also support a potential protective effect of TNF-alpha inhibitors against hypertension, providing additional genetic evidence for immune-targeted antihypertensive therapies. Furthermore, in the TSMR analysis, TNF-alpha inhibitors were also linked to a decreased risk of AS (39), highlighting their broader cardiovascular relevance.

Nevertheless, several limitations of this study merit acknowledgment. Firstly, due to the unavailability of eQTLs for PLA2G1B, the association between its gene expression and cardiovascular outcomes could not be assessed through SMR analysis. In addition, the comparatively small sample size of the GTEx V8 dataset may limit the statistical power to identify true associations. Secondly, the majority of the summary-level data used in this study was obtained from individuals of European ancestry, which may restrict the transferability of the results to other ethnic groups. Further investigations in more diverse populations are warranted to validate these results. Thirdly, although several sensitivity analyses were conducted to assess the robustness of the TSMR assumptions, the possibility of residual confounding or pleiotropy cannot be excluded. Relatedly, the selection of instruments based on CRP implicitly biases the findings toward inflammation-dependent mechanisms. This strategy may underestimate CRP-independent cardiovascular effects, particularly for DMARDs with pleiotropic actions involving endothelial, lipid metabolic, or thrombotic pathways. Fourthly, partial overlap between the GWASs for DMARDs and those for CVDs may have introduced bias. To mitigate this concern, the analysis was repeated using other GWAS datasets independent of or not solely based on UK Biobank participants. Lastly, as TSMR estimates reflect the cumulative effect of lifelong genetic exposure, they may not accurately represent the short-term pharmacologic effects of DMARDs (40). Furthermore, because TSMR estimates represent average genetic effects in the general population, the findings cannot be directly extrapolated to patients with RA, in whom cardiovascular risk is strongly influenced by disease activity, chronic inflammatory burden, and treatment timing. Consequently, both the direction and magnitude of the estimated effects may differ in RA populations, particularly among patients with high inflammatory activity or established cardiovascular disease. The TSMR findings therefore primarily inform the potential directionality of the causal effect rather than its precise magnitude and warrant validation through randomized controlled trials.

## Conclusion

In summary, this study presents genetic evidence indicating a potential causal association between DMARD target genes and the risk of CVDs. Future studies should prioritize targeted clinical trials, evaluate the effects of DMARDs on specific CVD phenotypes, and investigate the underlying mechanisms to comprehensively evaluate the therapeutic value of DMARDs in cardiovascular prevention.

## Data Accessibility Statement

All summary-level data used in this study can be accessed from publicly available repositories, as summarized in Table S1.

## Additional Files

The additional files for this article can be found as follows:

10.5334/gh.1526.s1 Supplementary File 1. Table S1–S11.

10.5334/gh.1526.s2 Supplementary File 2. Figure S1–S15.
