# Supplementary material for: Association of Disease-Modifying Antirheumatic Drugs (DMARDs) with Cardiovascular Diseases: Evidence from a Drug Target Mendelian Randomization Study
Source: Glob Heart. 2026 Feb 26;21(1):15. doi: 10.5334/gh.1526 (PMC12947826; doi:10.5334/gh.1526)
Supplement: Supplementary File 1. — Table S1–S11. [file gh-21-1-1526-s1.pdf]

**Table S1.** Summary of all data sources used in this study.

| Characteristic | Resource | Population | IEU GWAS ID | Data Source | Genome build | Statistical analysis |
|---|---|---|---|---|---|---|
| C-reactive protein | UK Biobank | European | ebi-a-GCST90025959 | https://gwas.mrcieu.ac.uk/datasets/ebi-a-GCST90025959/ | GRCh37 | Select IVs and coloclization analysis |
| C-reactive protein | UK Biobank and CHARGE Consortium | European | ebi-a-GCST90029070 | https://gwas.mrcieu.ac.uk/datasets/ebi-a-GCST90029070/ | GRCh37 | Select IVs in replication analysis |
| C-reactive protein | Ligthart et al | European | ieu-b-35 | https://gwas.mrcieu.ac.uk/datasets/ieu-b-35/ | GRCh37 | Select IVs in replication analysis |
| Rheumatoid arthritis | UK Biobank and FinnGen | Mixed | ebi-a-GCST90018910 | https://gwas.mrcieu.ac.uk/datasets/ebi-a-GCST90018910/ | GRCh37 | Positive control |
| Rheumatoid arthritis | Okada et al | European | ieu-a-832 | https://gwas.mrcieu.ac.uk/datasets/ieu-a-832/ | GRCh37 | Positive control |
| Atrial fibrillation | Nielsen et al | European | ebi-a-GCST006414 | https://gwas.mrcieu.ac.uk/datasets/ebi-a-GCST006414/ | GRCh37 | Outcomes |
| Coronary artery disease | CARDIoGRAMplusC4D | European | ieu-a-7 | https://gwas.mrcieu.ac.uk/datasets/ieu-a-7/ | GRCh37 | Outcomes |
| Myocardial infarction | CARDIoGRAMplusC4D | European | ieu-a-798 | https://gwas.mrcieu.ac.uk/datasets/ieu-a-798/ | GRCh37 | Outcomes |
| Heart failure | Shah et al | European | ebi-a-GCST009541 | https://gwas.mrcieu.ac.uk/datasets/ebi-a-GCST009541/ | GRCh37 | Outcomes |
| Ischemic stroke | Malik et al | European | ebi-a-GCST006908 | https://gwas.mrcieu.ac.uk/datasets/ebi-a-GCST006908/ | GRCh37 | Outcomes |
| Hypertension | FinnGen | European | finn-b-I9_HYPTENS | https://gwas.mrcieu.ac.uk/datasets/finn-b-I9_HYPTENS/ | GRCh37 | Outcomes |
| Total cholesterol | GLGC | Mixed | ieu-a-301 | https://gwas.mrcieu.ac.uk/datasets/ieu-a-301/ | GRCh37 | Outcomes |
| High-density lipoprotein cholesterol | GLGC | Mixed | ieu-a-299 | https://gwas.mrcieu.ac.uk/datasets/ieu-a-299/ | GRCh37 | Outcomes |
| Low-density lipoprotein cholesterol | GLGC | Mixed | ieu-a-300 | https://gwas.mrcieu.ac.uk/datasets/ieu-a-300/ | GRCh37 | Outcomes |
| Triglycerides | GLGC | Mixed | ieu-a-302 | https://gwas.mrcieu.ac.uk/datasets/ieu-a-302/ | GRCh37 | Outcomes |
| Aortic stenosis | Chen et al | European | / | https://zenodo.org/records/7829401 | GRCh37 | Outcomes |
| eQTLs | eQTLGen | Predominantly European | eqtl-a | https://www.eqtlgen.org/ | GRCh37 | SMR analysis |
| eQTLs | GTEx(V8) | Predominantly European American | / | https://yanglab.westlake.edu.cn/software/smr/#eQTLsummarydata | GRCh37 | SMR analysis |

**Table S2.** Drug targets of disease-modifying anti-rheumatic drugs (DMARDs) obtained from DrugBank.

| Drug Class | Subclass | ATC Code | Drug name | ATC Code | Target Gene(s) |
|---|---|---|---|---|---|
| Conventional synthetic DMARDs | Aminoquinolines | P01BA | Hydroxychloroquine | P01BA02 | TLR7,TLR9,ACE2 |
| | Aminosalicylic acid and similar agents | A07EC | Sulfasalazine | A07EC01 | ALOX5,PTGS2,PTGS1,ACAT1,PLA2G1B,IKBKB,REL,RELB,RELA,ABCG2,SLC7A11,NFKB1,NFKB2,CHUK,PPARG |
| | Calcineurin inhibitors | L04AD | Cyclosporine | L04AD01 | CAMLG,PPP3R2,PPIA,PPIF,PPP3CA |
| | Other immunosuppressants | L04AX | Methotrexate | L04AX03 | TYMS,ATIC,DHFR,SLC19A1,SLC46A1 |
| | Dihydroorotate dehydrogenase (DHODH) inhibitors | L04AK | Leflunomide | L04AK01 | DHODH,AHR,PTK2B |
| Biologic DMARDs | Tumor necrosis factor alpha (TNF-alpha) inhibitors | L04AB | Adalimumab | L04AB04 | TNF |
| | | | Golimumab | L04AB06 | TNF |
| | | | Certolizumab pegol | L04AB05 | TNF |
| | | | Infliximab | L04AB02 | TNF |
| | | | Etanercept | L04AB01 | TNF,LTA,FCGR1A,FCGR2A,FCGR2B,FCGR2C,FCGR3A,FCGR3B,C1QA,C1QB,C1QC |
| | Interleukin inhibitors | L04AC | Tocilizumab | L04AC07 | IL6R,IL6ST |
| | Selective immunosuppressants | L04AA | Abatacept | L04AA24 | CD80,CD86,CTLA4 |
| | Clusters of differentiation 20 (CD20) inhibitors | L01FA | Rituximab | L01FA01 | MS4A1 |
| Targeted synthetic DMARDs | Janus associated kinase (jak) inhibitors | L04AF | Tofacitinib | L04AF01 | JAK1,JAK2,JAK3,TYK2 |
| | | | Baricitinib | L04AF02 | JAK1,JAK2,JAK3,TYK2 |
| | | | Upadacitinib | L04AF03 | JAK1 |
| | | | Filgotinib | L04AF04 | JAK1 |

Classification of DMARDs from DrugBank, including drug classes, subclasses, Anatomical Therapeutic Chemical (ATC) codes, drug names, and corresponding target genes.

**Table S3.** Information and action of all target genes.

| Drug Subclass | Drug | Target Gene | Action | chromosome | Location |
|---|---|---|---|---|---|
| Aminoquinolines | Hydroxychloroquine | TLR7 | Antagonist | X | 12885191-12908480 |
| Aminoquinolines | Hydroxychloroquine | TLR9 | Antagonist | 3 | 52255096-52259661 |
| Aminoquinolines | Hydroxychloroquine | ACE2 | Modulator | X | 15536320-15625334 |
| Aminosalicylic acid and similar agents | Sulfasalazine | ALOX5 | Inhibitor | 10 | 45869664-45941565 |
| Aminosalicylic acid and similar agents | Sulfasalazine | PTGS2 | Inhibitor | 1 | 186640923-186649555 |
| Aminosalicylic acid and similar agents | Sulfasalazine | PTGS1 | Inhibitor | 9 | 125132812-125157982 |
| Aminosalicylic acid and similar agents | Sulfasalazine | ACAT1 | Inhibitor | 11 | 107987432-108018330 |
| Aminosalicylic acid and similar agents | Sulfasalazine | PLA2G1B | Antagonist | 12 | 120759918-120765582 |
| Aminosalicylic acid and similar agents | Sulfasalazine | IKBKB | Inhibitor | 8 | 42128820-42189978 |
| Aminosalicylic acid and similar agents | Sulfasalazine | REL | Activator | 2 | 61108709-61158747 |
| Aminosalicylic acid and similar agents | Sulfasalazine | RELB | Activator | 19 | 45504722-45541450 |
| Aminosalicylic acid and similar agents | Sulfasalazine | RELA | Activator | 11 | 65421072-65431328 |
| Aminosalicylic acid and similar agents | Sulfasalazine | ABCG2 | Inhibitor | 4 | 89011416-89152778 |
| Aminosalicylic acid and similar agents | Sulfasalazine | SLC7A11 | Inhibitor | 4 | 139085251-139163503 |
| Aminosalicylic acid and similar agents | Sulfasalazine | NFKB1 | Inhibitor | 4 | 103422516-103538459 |
| Aminosalicylic acid and similar agents | Sulfasalazine | NFKB2 | Inhibitor | 10 | 104153867-104162286 |
| Aminosalicylic acid and similar agents | Sulfasalazine | CHUK | Inhibitor | 10 | 101948057-101989353 |
| Aminosalicylic acid and similar agents | Sulfasalazine | PPARG | Agonist | 3 | 12328867-12475843 |
| Calcineurin inhibitors | Cyclosporine | CAMLG | Binder | 5 | 134074238-134087847 |
| Calcineurin inhibitors | Cyclosporine | PPP3R2 | Inhibitor | 9 | 104353886-104357251 |
| Calcineurin inhibitors | Cyclosporine | PPIA | Inhibitor;Binder | 7 | 44836280-44842716 |
| Calcineurin inhibitors | Cyclosporine | PPIF | Binder | 10 | 81107225-81115090 |
| Calcineurin inhibitors | Cyclosporine | PPP3CA | Inhibitor | 4 | 101944575-102268683 |

| | | | | | |
|---|---|---|---|---|---|
| Other immunosuppressants | Methotrexate | TYMS | Inhibitor | 18 | 657653-673578 |
| Other immunosuppressants | Methotrexate | ATIC | Inhibitor | 2 | 216176782-216214487 |
| Other immunosuppressants | Methotrexate | DHFR | Inhibitor | 5 | 79922045-79950802 |
| Other immunosuppressants | Methotrexate | SLC19A1 | Modulator | 21 | 46932461-46982939 |
| Other immunosuppressants | Methotrexate | SLC46A1 | Modulator | 17 | 26721661-26733230 |
| Dihydroorotate dehydrogenase (DHODH) inhibitors | Leflunomide | DHODH | Inhibitor | 16 | 72042643-72061558 |
| Dihydroorotate dehydrogenase (DHODH) inhibitors | Leflunomide | AHR | Agonist | 7 | 17338276-17385771 |
| Dihydroorotate dehydrogenase (DHODH) inhibitors | Leflunomide | PTK2B | Antagonist | 8 | 27168995-27316908 |
| Tumor necrosis factor alpha (TNF-alpha) inhibitors | Adalimumab/Golimumab/Certolizumab pegol/Infliximab | TNF | Inhibitor;Antibody | 6 | 31543342-31546113 |
| Tumor necrosis factor alpha (TNF-alpha) inhibitors | Etanercept | TNF | Inhibitor;Antibody | 6 | 31543342-31546113 |
| Tumor necrosis factor alpha (TNF-alpha) inhibitors | Etanercept | LTA | Antibody | 6 | 31539876-31542101 |
| Tumor necrosis factor alpha (TNF-alpha) inhibitors | Etanercept | FCGR1A | Ligand | 1 | 149754250-149772165 |
| Tumor necrosis factor alpha (TNF-alpha) inhibitors | Etanercept | FCGR2A | Ligand | 1 | 161475247-161489619 |
| Tumor necrosis factor alpha (TNF-alpha) inhibitors | Etanercept | FCGR2B | Ligand | 1 | 161617033-161648444 |
| Tumor necrosis factor alpha (TNF-alpha) inhibitors | Etanercept | FCGR2C | Ligand | 1 | 161551129-161571010 |
| Tumor necrosis factor alpha (TNF-alpha) inhibitors | Etanercept | FCGR3A | Ligand | 1 | 161511549-161520527 |
| Tumor necrosis factor alpha (TNF-alpha) inhibitors | Etanercept | FCGR3B | Ligand | 1 | 161592986-161601753 |
| Tumor necrosis factor alpha (TNF-alpha) inhibitors | Etanercept | C1QA | Ligand | 1 | 22962956-22966171 |
| Tumor necrosis factor alpha (TNF-alpha) inhibitors | Etanercept | C1QB | Ligand | 1 | 22979729-22988130 |
| Tumor necrosis factor alpha (TNF-alpha) inhibitors | Etanercept | C1QC | Ligand | 1 | 22970126-22974601 |
| Interleukin inhibitors | Tocilizumab | IL6R | Inhibitor | 1 | 154377819-154441926 |
| Interleukin inhibitors | Tocilizumab | IL6ST | Modulator | 5 | 55230923-55290791 |
| Selective immunosuppressants | Abatacept | CD80 | Antagonist | 3 | 119243140-119278461 |
| Selective immunosuppressants | Abatacept | CD86 | Antagonist | 3 | 121774209-121839983 |
| Clusters of differentiation 20 (CD20) inhibitors | Rituximab | MS4A1 | Antibody | 11 | 60223320-60238225 |
| Janus associated kinase (jak) inhibitors | Tofacitinib/Baricitinib | JAK1 | Inhibitor | 1 | 65298912-65533429 |

| | | | | | |
|---|---|---|---|---|---|
| Janus associated kinase (jak) inhibitors | Tofacitinib/Baricitinib | JAK2 | Inhibitor | 9 | 4984390-5129948 |
| Janus associated kinase (jak) inhibitors | Tofacitinib/Baricitinib | JAK3 | Inhibitor | 19 | 17935591-17958791 |
| Janus associated kinase (jak) inhibitors | Tofacitinib/Baricitinib | TYK2 | Inhibitor | 19 | 10461209-10491248 |
| Janus associated kinase (jak) inhibitors | Upadacitinib/Filgotinib | JAK1 | Inhibitor | 1 | 65298912-65533429 |

Information on drug target genes associated with various classes of DMARDs, with particular attention to their location and action.

**Table S4.** The single nucleotide polymorphisms (SNPs) and and their effects on C-reactive protein for each drug target.

| Drug | Target Gene | SNPs | Chr | Position | EA | OA | Beta | SE | P | F-Statistic | Included in Analysis |
|---|---|---|---|---|---|---|---|---|---|---|---|
| Cyclosporine | PPP3R2 | rs80188784 | 9 | 104118714 | T | C | 0.026 | 0.003 | 3.10E-21 | 88.49 | NO |
| Cyclosporine | PPIF | rs2012869 | 10 | 80853407 | A | C | -0.012 | 0.002 | 9.00E-09 | 31.68 | YES |
| Cyclosporine | PPIF | rs3740259 | 10 | 81074816 | A | G | 0.013 | 0.002 | 1.20E-08 | 32.36 | YES |
| Cyclosporine | PPIF | rs12360384 | 10 | 81182802 | A | G | -0.012 | 0.002 | 1.00E-10 | 32.87 | YES |
| Cyclosporine | PPIF | rs704015 | 10 | 80821208 | C | A | -0.015 | 0.003 | 1.20E-09 | 33.58 | YES |
| Cyclosporine | PPIF | rs704017 | 10 | 80819132 | G | A | -0.020 | 0.002 | 5.20E-24 | 96.07 | YES |
| Leflunomide | AHR | rs4410790 | 7 | 17284577 | C | T | -0.013 | 0.002 | 3.00E-11 | 41.50 | YES |
| Rituximab | MS4A1 | rs1151102 | 11 | 59815640 | G | A | -0.011 | 0.002 | 1.60E-08 | 28.50 | NO |
| Rituximab | MS4A1 | rs55661415 | 11 | 59952316 | G | C | -0.015 | 0.003 | 2.40E-09 | 34.08 | NO |
| Rituximab | MS4A1 | rs2583476 | 11 | 59857581 | A | G | -0.017 | 0.002 | 4.40E-19 | 68.80 | NO |
| Rituximab | MS4A1 | rs1562990 | 11 | 60023087 | A | C | 0.020 | 0.002 | 9.20E-24 | 92.20 | NO |
| Sulfasalazine | REL | rs35507645 | 2 | 61396766 | G | A | 0.012 | 0.002 | 3.30E-10 | 34.49 | NO |
| Sulfasalazine | ABCG2 | rs2231156 | 4 | 89020427 | A | C | -0.018 | 0.003 | 6.30E-09 | 28.12 | NO |
| Sulfasalazine | NFKB1 | rs13107325 | 4 | 103188709 | T | C | -0.026 | 0.004 | 6.20E-12 | 46.34 | NO |
| Sulfasalazine | RELA | rs629710 | 11 | 65109233 | C | A | 0.011 | 0.002 | 3.60E-08 | 25.40 | YES |
| Sulfasalazine | RELA | rs7115734 | 11 | 65458964 | A | G | 0.012 | 0.002 | 8.10E-09 | 35.03 | YES |
| Sulfasalazine | RELA | rs4565902 | 11 | 65810045 | G | T | 0.018 | 0.003 | 2.40E-10 | 42.82 | YES |
| Sulfasalazine | RELA | rs3741380 | 11 | 65349063 | A | G | 0.014 | 0.002 | 3.30E-11 | 47.08 | YES |
| Sulfasalazine | RELA | rs688862 | 11 | 65733393 | T | C | 0.014 | 0.002 | 1.10E-10 | 47.53 | YES |
| Sulfasalazine | PLA2G1B | rs206962 | 12 | 120855709 | T | C | 0.011 | 0.002 | 3.00E-08 | 28.71 | YES |
| Sulfasalazine | PLA2G1B | rs117692929 | 12 | 120884253 | C | G | 0.033 | 0.006 | 1.80E-09 | 29.40 | YES |
| Sulfasalazine | PLA2G1B | rs73405639 | 12 | 121039065 | G | T | -0.012 | 0.002 | 1.00E-08 | 29.79 | YES |
| Sulfasalazine | PLA2G1B | rs117633971 | 12 | 120540645 | A | G | 0.030 | 0.005 | 4.60E-08 | 31.22 | YES |
| Sulfasalazine | PLA2G1B | rs58759531 | 12 | 120731050 | T | C | 0.035 | 0.006 | 2.20E-09 | 32.06 | YES |
| Sulfasalazine | PLA2G1B | rs78860782 | 12 | 121107597 | G | A | -0.084 | 0.015 | 1.10E-08 | 32.94 | YES |
| Sulfasalazine | PLA2G1B | rs7294975 | 12 | 120546145 | G | C | 0.027 | 0.004 | 1.50E-10 | 36.68 | YES |
| Sulfasalazine | PLA2G1B | rs399956 | 12 | 12070906 | A | G | 0.015 | 0.002 | 8.50E-1 | 36.79 | YES |

| Drug | Target Gene | SNPs | Chr | Position | EA | OA | Beta | SE | P | F-Statistic | Included in Analysis |
|---|---|---|---|---|---|---|---|---|---|---|---|
| | | 1 | | 4 | | | | | 1 | | |
| Sulfasalazine | PLA2G1B | rs12322142 | 12 | 120875775 | C | T | 0.027 | 0.004 | 5.90E-10 | 36.91 | YES |
| Sulfasalazine | PLA2G1B | rs487915 | 12 | 121176549 | T | C | -0.027 | 0.004 | 9.60E-12 | 42.02 | YES |
| Sulfasalazine | PLA2G1B | rs73221336 | 12 | 120800988 | T | C | -0.025 | 0.004 | 3.10E-11 | 42.22 | YES |
| Sulfasalazine | PLA2G1B | rs188177542 | 12 | 120783870 | T | C | 0.056 | 0.009 | 2.60E-11 | 42.63 | YES |
| Sulfasalazine | PLA2G1B | rs17499109 | 12 | 120541570 | A | G | 0.021 | 0.003 | 4.20E-12 | 42.87 | YES |
| Sulfasalazine | PLA2G1B | rs74879960 | 12 | 120585236 | G | A | -0.027 | 0.004 | 4.00E-13 | 43.87 | YES |
| Sulfasalazine | PLA2G1B | rs118064568 | 12 | 120411054 | T | C | 0.042 | 0.006 | 2.50E-10 | 44.33 | YES |
| Sulfasalazine | PLA2G1B | rs111891989 | 12 | 120801099 | G | C | -0.028 | 0.004 | 1.80E-12 | 45.61 | YES |
| Sulfasalazine | PLA2G1B | rs7972240 | 12 | 120554802 | T | C | 0.026 | 0.004 | 4.70E-12 | 46.41 | YES |
| Sulfasalazine | PLA2G1B | rs77123459 | 12 | 120717111 | A | G | 0.042 | 0.006 | 3.20E-12 | 47.05 | YES |
| Sulfasalazine | PLA2G1B | rs2422 | 12 | 120565188 | A | G | 0.015 | 0.002 | 6.50E-14 | 53.84 | YES |
| Sulfasalazine | PLA2G1B | rs25664 | 12 | 120650264 | A | G | 0.036 | 0.005 | 6.20E-14 | 54.33 | YES |
| Sulfasalazine | PLA2G1B | rs73411916 | 12 | 121208238 | C | T | 0.042 | 0.006 | 9.00E-14 | 55.07 | YES |
| Sulfasalazine | PLA2G1B | rs114484676 | 12 | 120730903 | T | C | 0.047 | 0.006 | 1.10E-13 | 56.16 | YES |
| Sulfasalazine | PLA2G1B | rs78686363 | 12 | 121157983 | A | G | -0.045 | 0.006 | 4.00E-14 | 56.62 | YES |
| Sulfasalazine | PLA2G1B | rs117763632 | 12 | 120850421 | T | C | 0.090 | 0.012 | 1.90E-14 | 58.88 | YES |
| Sulfasalazine | PLA2G1B | rs117468623 | 12 | 120688572 | C | T | 0.060 | 0.008 | 2.20E-15 | 60.37 | YES |
| Sulfasalazine | PLA2G1B | rs61945816 | 12 | 120541599 | A | G | -0.057 | 0.007 | 5.50E-16 | 61.06 | YES |
| Sulfasalazine | PLA2G1B | rs77205232 | 12 | 121231816 | C | A | 0.062 | 0.008 | 6.90E-16 | 63.18 | YES |
| Sulfasalazine | PLA2G1B | rs113684685 | 12 | 120730954 | A | G | -0.062 | 0.008 | 1.70E-17 | 64.34 | YES |
| Sulfasalazine | PLA2G1B | rs117803005 | 12 | 120963926 | G | A | -0.056 | 0.007 | 1.10E-15 | 64.71 | YES |
| Sulfasalazine | PLA2G1B | rs79616458 | 12 | 121093487 | C | T | -0.103 | 0.013 | 1.60E-16 | 64.94 | YES |
| Sulfasalazine | PLA2G1B | rs35151295 | 12 | 121040529 | A | G | 0.082 | 0.010 | 6.60E-18 | 68.08 | YES |
| Sulfasalazine | PLA2G1B | rs191322615 | 12 | 120554466 | A | G | -0.039 | 0.005 | 2.20E-19 | 72.29 | YES |
| Sulfasalazine | PLA2G1B | rs2859282 | 12 | 120859363 | A | G | -0.029 | 0.003 | 7.60E-18 | 72.32 | YES |
| Sulfasalazine | PLA2G1B | rs113116967 | 12 | 120430074 | T | G | -0.038 | 0.004 | 8.10E-20 | 73.42 | YES |
| Sulfasalazine | PLA2G1B | rs4767915 | 12 | 120999127 | A | G | -0.029 | 0.003 | 4.50E-20 | 74.66 | YES |
| Sulfasalazine | PLA2G1B | rs110652 | 12 | 12114621 | T | C | -0.028 | 0.003 | 6.00E-2 | 76.51 | YES |

| Drug | Target Gene | SNPs | Chr | Position | EA | OA | Beta | SE | P | F-Statistic | Included in Analysis |
|---|---|---|---|---|---|---|---|---|---|---|---|
| | | 19 | | 9 | | | | | 0 | | |
| Sulfasalazine | PLA2G1B | rs4767878 | 12 | 120564999 | G | A | 0.057 | 0.006 | 2.90E-21 | 84.56 | YES |
| Sulfasalazine | PLA2G1B | rs7959743 | 12 | 120859417 | T | C | -0.028 | 0.003 | 9.00E-23 | 88.87 | YES |
| Sulfasalazine | PLA2G1B | rs1039302 | 12 | 121236258 | T | C | 0.026 | 0.003 | 2.50E-22 | 89.43 | YES |
| Sulfasalazine | PLA2G1B | rs78268708 | 12 | 121197324 | C | G | 0.044 | 0.005 | 4.20E-24 | 92.10 | YES |
| Sulfasalazine | PLA2G1B | rs76475417 | 12 | 120870774 | A | G | -0.057 | 0.006 | 4.70E-24 | 92.41 | YES |
| Sulfasalazine | PLA2G1B | rs4767891 | 12 | 120863422 | A | G | -0.021 | 0.002 | 5.00E-24 | 96.98 | YES |
| Sulfasalazine | PLA2G1B | rs11065079 | 12 | 120752247 | T | C | -0.069 | 0.007 | 3.10E-25 | 98.53 | YES |
| Sulfasalazine | PLA2G1B | rs73222784 | 12 | 121088369 | A | G | -0.048 | 0.005 | 6.70E-26 | 106.82 | YES |
| Sulfasalazine | PLA2G1B | rs10849778 | 12 | 121135508 | A | G | -0.031 | 0.003 | 6.80E-27 | 108.13 | YES |
| Sulfasalazine | PLA2G1B | rs1076205 | 12 | 120783527 | A | G | 0.022 | 0.002 | 5.20E-28 | 108.20 | YES |
| Sulfasalazine | PLA2G1B | rs76841017 | 12 | 121090195 | C | T | -0.063 | 0.006 | 3.40E-27 | 108.58 | YES |
| Sulfasalazine | PLA2G1B | rs11065126 | 12 | 120880707 | A | G | -0.066 | 0.006 | 4.30E-29 | 112.80 | YES |
| Sulfasalazine | PLA2G1B | rs34236994 | 12 | 120654084 | C | G | -0.033 | 0.003 | 1.40E-27 | 114.60 | YES |
| Sulfasalazine | PLA2G1B | rs117807066 | 12 | 120929463 | T | G | -0.066 | 0.006 | 6.20E-35 | 136.92 | YES |
| Sulfasalazine | PLA2G1B | rs80097744 | 12 | 121079806 | T | C | 0.034 | 0.003 | 1.40E-38 | 155.41 | YES |
| Sulfasalazine | PLA2G1B | rs116881341 | 12 | 120737796 | A | G | -0.095 | 0.007 | 1.50E-40 | 163.31 | YES |
| Sulfasalazine | PLA2G1B | rs7953949 | 12 | 120687935 | C | G | -0.040 | 0.003 | 4.60E-43 | 168.74 | YES |
| Sulfasalazine | PLA2G1B | rs34179846 | 12 | 120917722 | C | T | 0.045 | 0.003 | 1.10E-44 | 191.11 | YES |
| Sulfasalazine | PLA2G1B | rs7960743 | 12 | 120720038 | C | T | 0.035 | 0.003 | 1.60E-45 | 192.01 | YES |
| Sulfasalazine | PLA2G1B | rs56401401 | 12 | 121186314 | A | G | -0.077 | 0.006 | 1.20E-45 | 192.19 | YES |
| Sulfasalazine | PLA2G1B | rs2239206 | 12 | 120676090 | A | G | 0.028 | 0.002 | 1.40E-45 | 192.96 | YES |
| Sulfasalazine | PLA2G1B | rs73229142 | 12 | 121257090 | C | T | 0.067 | 0.004 | 1.80E-51 | 218.79 | YES |
| Sulfasalazine | PLA2G1B | rs555404 | 12 | 121175984 | C | T | -0.032 | 0.002 | 3.20E-61 | 250.47 | YES |
| Sulfasalazine | PLA2G1B | rs71454665 | 12 | 121198711 | T | C | 0.073 | 0.004 | 1.80E-71 | 291.00 | YES |
| Sulfasalazine | PLA2G1B | rs10849772 | 12 | 121070251 | A | G | -0.087 | 0.005 | 1.30E-82 | 333.69 | YES |
| Sulfasalazine | PLA2G1B | rs560736 | 12 | 121138350 | T | G | 0.111 | 0.006 | 3.40E-80 | 335.44 | YES |
| Sulfasalazine | PLA2G1B | rs1800556 | 12 | 121175678 | T | C | 0.087 | 0.005 | 5.70E-88 | 364.18 | YES |
| Sulfasalazine | PLA2G1B | rs730248 | 12 | 12105218 | C | T | 0.080 | 0.004 | 5.70E-1 | 464.12 | YES |

| Drug | Target Gene | SNPs | Chr | Position | EA | OA | Beta | SE | P | F-Statistic | Included in Analysis |
|---|---|---|---|---|---|---|---|---|---|---|---|
| | | 2 | | 0 | | | | | 11 | | |
| Sulfasalazine | PLA2G1B | rs7137504 | 12 | 121221628 | T | C | -0.049 | 0.002 | 4.40E-140 | 601.06 | YES |
| Sulfasalazine | PLA2G1B | rs609394 | 12 | 121213592 | A | G | 0.071 | 0.003 | 2.50E-162 | 703.06 | YES |
| Sulfasalazine | PLA2G1B | rs569499 | 12 | 121094110 | C | T | 0.067 | 0.002 | 1.00E-200 | 1076.11 | YES |
| Sulfasalazine | PLA2G1B | rs57830821 | 12 | 121122676 | T | C | -0.101 | 0.003 | 1.00E-200 | 1292.28 | YES |
| Sulfasalazine | RELB | rs8100718 | 19 | 45016116 | G | A | 0.010 | 0.002 | 1.00E-08 | 24.61 | YES |
| Sulfasalazine | RELB | rs78073763 | 19 | 45649838 | G | T | 0.018 | 0.003 | 1.90E-08 | 25.41 | YES |
| Sulfasalazine | RELB | rs17800819 | 19 | 45017064 | T | C | 0.015 | 0.003 | 5.70E-09 | 25.85 | YES |
| Sulfasalazine | RELB | rs2965160 | 19 | 45196135 | G | A | 0.011 | 0.002 | 5.80E-09 | 26.63 | YES |
| Sulfasalazine | RELB | rs1004165 | 19 | 45232205 | A | G | -0.010 | 0.002 | 3.80E-08 | 26.84 | YES |
| Sulfasalazine | RELB | rs28381261 | 19 | 45979984 | A | G | -0.019 | 0.004 | 4.80E-08 | 27.68 | YES |
| Sulfasalazine | RELB | rs10419931 | 19 | 45893476 | T | G | 0.013 | 0.002 | 2.10E-08 | 29.09 | YES |
| Sulfasalazine | RELB | rs139295630 | 19 | 45262848 | A | C | 0.035 | 0.006 | 1.60E-08 | 29.59 | YES |
| Sulfasalazine | RELB | rs75792246 | 19 | 45682876 | G | C | 0.027 | 0.005 | 8.70E-09 | 31.92 | YES |
| Sulfasalazine | RELB | rs73035602 | 19 | 45200681 | T | G | 0.021 | 0.004 | 1.60E-08 | 32.12 | YES |
| Sulfasalazine | RELB | rs16979595 | 19 | 45477381 | A | G | -0.016 | 0.003 | 1.10E-08 | 32.93 | YES |
| Sulfasalazine | RELB | rs73049903 | 19 | 45966736 | T | C | 0.020 | 0.004 | 3.00E-09 | 33.29 | YES |
| Sulfasalazine | RELB | rs60239918 | 19 | 45580372 | T | C | -0.029 | 0.005 | 6.10E-09 | 33.61 | YES |
| Sulfasalazine | RELB | rs80000866 | 19 | 45200634 | T | C | 0.025 | 0.004 | 8.70E-09 | 35.28 | YES |
| Sulfasalazine | RELB | rs62116896 | 19 | 45035074 | T | C | -0.018 | 0.003 | 2.40E-10 | 35.49 | YES |
| Sulfasalazine | RELB | rs2282696 | 19 | 45972039 | C | A | 0.012 | 0.002 | 6.20E-09 | 35.61 | YES |
| Sulfasalazine | RELB | rs71352236 | 19 | 45375413 | A | G | 0.045 | 0.007 | 2.40E-09 | 37.30 | YES |
| Sulfasalazine | RELB | rs140157080 | 19 | 45567648 | G | C | 0.038 | 0.006 | 6.20E-10 | 37.93 | YES |
| Sulfasalazine | RELB | rs189063316 | 19 | 45716364 | T | C | -0.047 | 0.008 | 7.70E-11 | 38.66 | YES |
| Sulfasalazine | RELB | rs28399663 | 19 | 45323801 | T | C | 0.035 | 0.005 | 1.20E-10 | 40.18 | YES |
| Sulfasalazine | RELB | rs77766022 | 19 | 45662432 | A | G | -0.045 | 0.007 | 3.00E-10 | 40.62 | YES |
| Sulfasalazine | RELB | rs874743 | 19 | 45513236 | A | G | -0.045 | 0.007 | 2.30E-11 | 41.53 | YES |
| Sulfasalazine | RELB | rs74827760 | 19 | 45790300 | T | C | 0.035 | 0.005 | 1.40E-12 | 47.34 | YES |
| Sulfasalazine | RELB | rs296517 | 19 | 45245015 | A | G | 0.014 | 0.002 | 6.10E-1 | 50.89 | YES |

| Drug | Target Gene | SNPs | Chr | Position | EA | OA | Beta | SE | P | F-Statistic | Included in Analysis |
|---|---|---|---|---|---|---|---|---|---|---|---|
| | | 4 | | | | | | | 3 | | |
| Sulfasalazine | RELB | rs12973246 | 19 | 45836204 | A | G | 0.015 | 0.002 | 1.40E-13 | 51.84 | YES |
| Sulfasalazine | RELB | rs116958755 | 19 | 45690282 | G | T | -0.040 | 0.005 | 5.50E-15 | 53.67 | YES |
| Sulfasalazine | RELB | rs55848260 | 19 | 45080369 | A | G | -0.015 | 0.002 | 9.70E-14 | 53.99 | YES |
| Sulfasalazine | RELB | rs875255 | 19 | 45493635 | G | C | -0.015 | 0.002 | 7.30E-14 | 54.33 | YES |
| Sulfasalazine | RELB | rs75161053 | 19 | 45048858 | A | G | -0.034 | 0.005 | 7.20E-15 | 54.38 | YES |
| Sulfasalazine | RELB | rs28399635 | 19 | 45323243 | G | A | 0.019 | 0.003 | 5.90E-14 | 54.77 | YES |
| Sulfasalazine | RELB | rs62119261 | 19 | 45122043 | C | A | 0.035 | 0.005 | 7.80E-16 | 55.72 | YES |
| Sulfasalazine | RELB | rs77653204 | 19 | 45614835 | G | A | 0.036 | 0.005 | 1.40E-14 | 56.10 | YES |
| Sulfasalazine | RELB | rs1799783 | 19 | 45872036 | T | C | 0.016 | 0.002 | 3.80E-16 | 61.13 | YES |
| Sulfasalazine | RELB | rs3178166 | 19 | 45594170 | G | A | 0.016 | 0.002 | 4.10E-15 | 61.65 | YES |
| Sulfasalazine | RELB | rs62120566 | 19 | 45197732 | G | C | 0.051 | 0.006 | 5.20E-17 | 62.77 | YES |
| Sulfasalazine | RELB | rs113708952 | 19 | 45595003 | C | A | 0.022 | 0.003 | 5.70E-16 | 65.51 | YES |
| Sulfasalazine | RELB | rs846848 | 19 | 45074177 | T | G | -0.023 | 0.003 | 1.80E-18 | 68.70 | YES |
| Sulfasalazine | RELB | rs8109764 | 19 | 45722743 | A | G | -0.046 | 0.005 | 2.60E-19 | 71.74 | YES |
| Sulfasalazine | RELB | rs41290102 | 19 | 45371188 | T | C | 0.072 | 0.008 | 4.00E-19 | 74.77 | YES |
| Sulfasalazine | RELB | rs519113 | 19 | 45376284 | G | C | 0.020 | 0.002 | 4.30E-18 | 78.31 | YES |
| Sulfasalazine | RELB | rs80257887 | 19 | 45020859 | A | G | -0.047 | 0.005 | 9.40E-21 | 78.38 | YES |
| Sulfasalazine | RELB | rs74607435 | 19 | 45235700 | C | T | 0.040 | 0.005 | 3.20E-22 | 80.38 | YES |
| Sulfasalazine | RELB | rs147211387 | 19 | 45261755 | A | G | -0.076 | 0.008 | 6.30E-22 | 83.62 | YES |
| Sulfasalazine | RELB | rs3810141 | 19 | 45316804 | T | C | 0.040 | 0.004 | 4.30E-22 | 83.95 | YES |
| Sulfasalazine | RELB | rs10415983 | 19 | 45711598 | T | C | -0.025 | 0.003 | 1.40E-23 | 85.33 | YES |
| Sulfasalazine | RELB | rs143674704 | 19 | 45458466 | G | A | -0.071 | 0.008 | 4.70E-21 | 86.25 | YES |
| Sulfasalazine | RELB | rs201269048 | 19 | 45574113 | G | T | 0.044 | 0.005 | 2.80E-20 | 87.06 | YES |
| Sulfasalazine | RELB | rs76419583 | 19 | 45217859 | C | T | -0.027 | 0.003 | 5.40E-24 | 93.61 | YES |
| Sulfasalazine | RELB | rs3208856 | 19 | 45296806 | T | C | 0.053 | 0.005 | 4.10E-25 | 97.52 | YES |
| Sulfasalazine | RELB | rs346751 | 19 | 45734433 | C | T | 0.020 | 0.002 | 6.40E-25 | 102.53 | YES |
| Sulfasalazine | RELB | rs7260482 | 19 | 45143942 | C | A | 0.023 | 0.002 | 1.70E-26 | 107.85 | YES |
| Sulfasalazine | RELB | rs106472 | 19 | 45422561 | G | T | 0.057 | 0.005 | 4.10E-2 | 108.50 | YES |

| Drug | Target Gene | SNPs | Chr | Position | EA | OA | Beta | SE | P | F-Statistic | Included in Analysis |
|---|---|---|---|---|---|---|---|---|---|---|---|
| | | 5 | | | | | | | 7 | | |
| Sulfasalazine | RELB | rs139425456 | 19 | 45297373 | A | T | -0.083 | 0.008 | 4.90E-28 | 108.57 | YES |
| Sulfasalazine | RELB | rs78620885 | 19 | 45591084 | T | C | -0.062 | 0.006 | 1.70E-27 | 113.82 | YES |
| Sulfasalazine | RELB | rs117326714 | 19 | 45287103 | G | A | 0.068 | 0.006 | 7.20E-29 | 121.59 | YES |
| Sulfasalazine | RELB | rs2965156 | 19 | 45188429 | C | G | 0.022 | 0.002 | 3.80E-34 | 125.25 | YES |
| Sulfasalazine | RELB | rs2306149 | 19 | 45349963 | A | C | 0.023 | 0.002 | 8.50E-33 | 129.39 | YES |
| Sulfasalazine | RELB | rs73033507 | 19 | 45431403 | T | C | 0.060 | 0.005 | 4.70E-29 | 131.90 | YES |
| Sulfasalazine | RELB | rs11881756 | 19 | 45220896 | C | T | 0.036 | 0.003 | 4.70E-35 | 134.03 | YES |
| Sulfasalazine | RELB | rs10405086 | 19 | 45627235 | T | C | -0.041 | 0.003 | 1.70E-35 | 141.85 | YES |
| Sulfasalazine | RELB | rs79429216 | 19 | 45445517 | A | G | -0.115 | 0.009 | 6.50E-37 | 151.68 | YES |
| Sulfasalazine | RELB | rs12721109 | 19 | 45447221 | A | G | 0.079 | 0.006 | 3.40E-38 | 157.67 | YES |
| Sulfasalazine | RELB | rs2965164 | 19 | 45202052 | T | C | 0.027 | 0.002 | 7.40E-41 | 165.23 | YES |
| Sulfasalazine | RELB | rs62117160 | 19 | 45232161 | A | G | 0.062 | 0.005 | 1.10E-42 | 168.46 | YES |
| Sulfasalazine | RELB | rs2927437 | 19 | 45241638 | G | A | 0.033 | 0.003 | 1.10E-43 | 174.15 | YES |
| Sulfasalazine | RELB | rs714948 | 19 | 45165912 | A | C | -0.042 | 0.003 | 2.50E-45 | 186.84 | YES |
| Sulfasalazine | RELB | rs204906 | 19 | 45461980 | T | C | 0.080 | 0.006 | 5.30E-47 | 206.38 | YES |
| Sulfasalazine | RELB | rs72654472 | 19 | 45414392 | T | G | 0.092 | 0.006 | 1.50E-55 | 227.36 | YES |
| Sulfasalazine | RELB | rs2965169 | 19 | 45251156 | C | A | 0.031 | 0.002 | 3.10E-58 | 232.35 | YES |
| Sulfasalazine | RELB | rs117264457 | 19 | 45404432 | A | G | 0.100 | 0.007 | 5.50E-56 | 232.60 | YES |
| Sulfasalazine | RELB | rs283814 | 19 | 45389224 | G | A | -0.060 | 0.004 | 2.00E-61 | 252.59 | YES |
| Sulfasalazine | RELB | rs1160984 | 19 | 45403924 | T | C | 0.078 | 0.004 | 9.50E-78 | 333.35 | YES |
| Sulfasalazine | RELB | rs393584 | 19 | 45377334 | A | G | 0.042 | 0.002 | 4.40E-89 | 390.08 | YES |
| Sulfasalazine | RELB | rs7412 | 19 | 45412079 | T | C | 0.099 | 0.004 | 5.70E-173 | 731.61 | YES |
| Sulfasalazine | RELB | rs10405693 | 19 | 45326664 | T | C | -0.059 | 0.002 | 1.20E-179 | 762.07 | YES |
| Sulfasalazine | RELB | rs6859 | 19 | 45382034 | G | A | 0.058 | 0.002 | 1.50E-192 | 815.34 | YES |
| Sulfasalazine | RELB | rs28399637 | 19 | 45324138 | A | G | -0.064 | 0.002 | 1.00E-200 | 850.70 | YES |
| Sulfasalazine | RELB | rs41289512 | 19 | 45351516 | G | C | -0.184 | 0.005 | 1.00E-200 | 1410.09 | YES |
| Sulfasalazine | RELB | rs77301115 | 19 | 45396973 | A | G | -0.252 | 0.006 | 1.00E-200 | 1584.67 | YES |
| Sulfasalazine | RELB | rs157580 | 19 | 45395266 | A | G | -0.092 | 0.002 | 1.00E-2 | 2056.84 | YES |

| Drug | Target Gene | SNPs | Chr | Position | EA | OA | Beta | SE | P | F-Statistic | Included in Analysis |
|---|---|---|---|---|---|---|---|---|---|---|---|
| | | | | | | | | | 00 | | |
| Sulfasalazine | RELB | rs184017 | 19 | 45394969 | G | T | -0.187 | 0.002 | 1.00E-200 | 5921.39 | YES |
| Abatacept | CD80 | rs3732356 | 3 | 119529113 | T | G | -0.029 | 0.004 | 7.00E-14 | 52.40 | YES |
| Abatacept | CD80 | rs6805251 | 3 | 119560606 | C | T | -0.016 | 0.002 | 1.00E-14 | 58.08 | YES |
| Abatacept | CD80 | rs7644234 | 3 | 119691611 | T | G | 0.016 | 0.002 | 1.80E-15 | 62.37 | YES |
| TNF-alpha inhibitors | TNF/LTA | rs3869132 | 6 | 31410948 | T | C | 0.017 | 0.003 | 4.00E-08 | 27.92 | YES |
| TNF-alpha inhibitors | TNF/LTA | rs2507992 | 6 | 31322459 | C | T | 0.017 | 0.003 | 2.70E-08 | 29.52 | YES |
| TNF-alpha inhibitors | TNF/LTA | rs2596501 | 6 | 31321211 | T | C | -0.016 | 0.003 | 3.90E-08 | 31.39 | YES |
| TNF-alpha inhibitors | TNF/LTA | rs9266658 | 6 | 31347644 | A | G | 0.018 | 0.003 | 4.40E-09 | 33.42 | YES |
| TNF-alpha inhibitors | TNF/LTA | rs1140546 | 6 | 31322888 | G | A | 0.019 | 0.003 | 4.70E-10 | 37.19 | YES |
| TNF-alpha inhibitors | TNF/LTA | rs2844510 | 6 | 31410408 | T | G | 0.027 | 0.004 | 1.10E-11 | 41.93 | YES |
| TNF-alpha inhibitors | TNF/LTA | rs3180379 | 6 | 31324491 | A | C | 0.029 | 0.004 | 1.60E-12 | 43.39 | YES |
| TNF-alpha inhibitors | TNF/LTA | rs9468944 | 6 | 31274582 | A | G | 0.024 | 0.003 | 7.80E-11 | 45.40 | YES |
| TNF-alpha inhibitors | TNF/LTA | rs2239704 | 6 | 31540141 | C | A | 0.021 | 0.003 | 6.80E-14 | 53.12 | YES |
| TNF-alpha inhibitors | TNF/LTA | rs142973694 | 6 | 31362941 | A | G | 0.054 | 0.006 | 1.70E-23 | 90.31 | YES |
| TNF-alpha inhibitors | FCGR1A | rs1868992 | 1 | 149908108 | G | A | -0.011 | 0.002 | 2.60E-08 | 24.71 | NO |
| TNF-alpha inhibitors | FCGR1A | rs11205277 | 1 | 149892872 | G | A | 0.010 | 0.002 | 4.20E-08 | 28.20 | NO |
| TNF-alpha inhibitors | FCGR1A | rs72692825 | 1 | 149920593 | A | C | 0.026 | 0.005 | 1.10E-09 | 30.87 | NO |
| TNF-alpha inhibitors | C1QA | rs6426749 | 1 | 22711473 | C | G | -0.015 | 0.003 | 2.60E-09 | 35.71 | NO |
| TNF-alpha inhibitors | C1QB | rs6426749 | 1 | 22711473 | C | G | -0.015 | 0.003 | 2.60E-09 | 35.71 | NO |
| TNF-alpha inhibitors | C1QC | rs6426749 | 1 | 22711473 | C | G | -0.015 | 0.003 | 2.60E-09 | 35.71 | NO |
| IL-6 inhibitors | IL6R | rs6664817 | 1 | 154684106 | T | G | -0.024 | 0.005 | 2.70E-08 | 27.58 | YES |
| IL-6 inhibitors | IL6R | rs116141616 | 1 | 154416069 | A | G | 0.031 | 0.006 | 1.90E-08 | 28.42 | YES |
| IL-6 inhibitors | IL6R | rs41265215 | 1 | 154164063 | G | A | 0.033 | 0.006 | 1.80E-09 | 32.25 | YES |
| IL-6 inhibitors | IL6R | rs8192484 | 1 | 154541839 | T | A | 0.041 | 0.007 | 2.90E-09 | 33.59 | YES |
| IL-6 inhibitors | IL6R | rs115891382 | 1 | 154277834 | C | T | 0.031 | 0.005 | 1.90E-09 | 33.70 | YES |
| IL-6 inhibitors | IL6R | rs17366139 | 1 | 154090501 | A | C | 0.018 | 0.003 | 1.60E-09 | 33.88 | YES |
| IL-6 inhibitors | IL6R | rs77243303 | 1 | 154856891 | T | C | 0.045 | 0.008 | 1.10E-09 | 35.67 | YES |
| IL-6 inhibitors | IL6R | rs127262 | 1 | 15417155 | G | A | -0.027 | 0.004 | 4.00E-1 | 39.14 | YES |

| Drug | Target Gene | SNPs | Chr | Position | EA | OA | Beta | SE | P | F-Statistic | Included in Analysis |
|---|---|---|---|---|---|---|---|---|---|---|---|
| | | 20 | | 0 | | | | | 1 | | |
| IL-6 inhibitors | IL6R | rs114697636 | 1 | 154127549 | G | C | 0.032 | 0.005 | 6.00E-11 | 39.42 | YES |
| IL-6 inhibitors | IL6R | rs72694260 | 1 | 153914261 | G | C | 0.022 | 0.004 | 2.10E-10 | 40.06 | YES |
| IL-6 inhibitors | IL6R | rs112203594 | 1 | 154553430 | A | C | 0.038 | 0.006 | 1.10E-10 | 40.39 | YES |
| IL-6 inhibitors | IL6R | rs111303174 | 1 | 154677979 | A | C | 0.036 | 0.006 | 3.50E-11 | 42.69 | YES |
| IL-6 inhibitors | IL6R | rs114445392 | 1 | 154438143 | T | C | -0.051 | 0.008 | 2.40E-10 | 44.63 | YES |
| IL-6 inhibitors | IL6R | rs115437600 | 1 | 154361126 | A | G | 0.030 | 0.004 | 3.00E-12 | 44.86 | YES |
| IL-6 inhibitors | IL6R | rs115462819 | 1 | 154265961 | T | C | 0.040 | 0.006 | 5.30E-11 | 46.96 | YES |
| IL-6 inhibitors | IL6R | rs35717427 | 1 | 154391882 | A | G | -0.020 | 0.003 | 4.10E-12 | 47.07 | YES |
| IL-6 inhibitors | IL6R | rs144029367 | 1 | 154455249 | C | T | 0.051 | 0.007 | 1.50E-12 | 50.07 | YES |
| IL-6 inhibitors | IL6R | rs77994054 | 1 | 154699197 | T | C | -0.051 | 0.007 | 4.20E-13 | 50.79 | YES |
| IL-6 inhibitors | IL6R | rs4845663 | 1 | 154692088 | C | T | 0.015 | 0.002 | 8.20E-15 | 54.40 | YES |
| IL-6 inhibitors | IL6R | rs58892549 | 1 | 154688488 | C | T | -0.032 | 0.004 | 4.00E-15 | 59.13 | YES |
| IL-6 inhibitors | IL6R | rs113191295 | 1 | 153912623 | A | G | -0.033 | 0.004 | 8.60E-18 | 63.87 | YES |
| IL-6 inhibitors | IL6R | rs78038982 | 1 | 154539156 | T | G | 0.060 | 0.007 | 2.10E-16 | 65.97 | YES |
| IL-6 inhibitors | IL6R | rs2274988 | 1 | 154316434 | T | C | -0.018 | 0.002 | 1.20E-16 | 66.58 | YES |
| IL-6 inhibitors | IL6R | rs41269913 | 1 | 154461480 | T | C | -0.042 | 0.005 | 2.20E-15 | 67.00 | YES |
| IL-6 inhibitors | IL6R | rs75164063 | 1 | 154036161 | A | G | -0.056 | 0.006 | 1.20E-19 | 78.70 | YES |
| IL-6 inhibitors | IL6R | rs150059283 | 1 | 153912533 | G | A | -0.085 | 0.009 | 5.10E-22 | 88.59 | YES |
| IL-6 inhibitors | IL6R | rs55997241 | 1 | 154671128 | A | G | 0.035 | 0.004 | 4.10E-22 | 89.09 | YES |
| IL-6 inhibitors | IL6R | rs41310887 | 1 | 154321623 | A | G | 0.044 | 0.005 | 1.40E-23 | 91.50 | YES |
| IL-6 inhibitors | IL6R | rs60517797 | 1 | 154642224 | C | T | 0.034 | 0.004 | 6.60E-24 | 93.42 | YES |
| IL-6 inhibitors | IL6R | rs2230324 | 1 | 153954646 | T | C | -0.048 | 0.005 | 1.80E-22 | 96.92 | YES |
| IL-6 inhibitors | IL6R | rs76289529 | 1 | 154516404 | T | C | -0.051 | 0.005 | 4.30E-24 | 98.11 | YES |
| IL-6 inhibitors | IL6R | rs77993403 | 1 | 154316996 | A | G | -0.047 | 0.005 | 2.20E-23 | 99.10 | YES |
| IL-6 inhibitors | IL6R | rs16835819 | 1 | 154108501 | C | T | -0.089 | 0.009 | 2.60E-25 | 103.69 | YES |
| IL-6 inhibitors | IL6R | rs1194611 | 1 | 154301246 | A | C | -0.021 | 0.002 | 1.30E-25 | 108.75 | YES |
| IL-6 inhibitors | IL6R | rs116805289 | 1 | 154510155 | C | A | 0.068 | 0.006 | 3.80E-26 | 109.31 | YES |
| IL-6 inhibitors | IL6R | rs413108 | 1 | 15451426 | T | C | 0.048 | 0.005 | 6.50E-2 | 112.75 | YES |

| Drug | Target Gene | SNPs | Chr | Position | EA | OA | Beta | SE | P | F-Statistic | Included in Analysis |
|---|---|---|---|---|---|---|---|---|---|---|---|
| | | 93 | | 7 | | | | | 8 | | |
| IL-6 inhibitors | IL6R | rs61806853 | 1 | 154154587 | C | T | -0.048 | 0.004 | 8.20E-27 | 116.15 | YES |
| IL-6 inhibitors | IL6R | rs77801962 | 1 | 154566932 | C | T | -0.062 | 0.006 | 2.90E-29 | 117.66 | YES |
| IL-6 inhibitors | IL6R | rs6668968 | 1 | 154293675 | A | G | -0.023 | 0.002 | 1.20E-30 | 123.34 | YES |
| IL-6 inhibitors | IL6R | rs79794939 | 1 | 154390932 | T | C | 0.045 | 0.004 | 5.10E-35 | 145.84 | YES |
| IL-6 inhibitors | IL6R | rs9427092 | 1 | 154553722 | C | T | 0.033 | 0.002 | 5.20E-44 | 177.66 | YES |
| IL-6 inhibitors | IL6R | rs11265608 | 1 | 154364140 | A | G | 0.044 | 0.003 | 7.90E-46 | 190.24 | YES |
| IL-6 inhibitors | IL6R | rs115718964 | 1 | 154290747 | A | G | -0.090 | 0.006 | 3.70E-44 | 194.49 | YES |
| IL-6 inhibitors | IL6R | rs12072348 | 1 | 154547292 | C | A | 0.047 | 0.003 | 1.30E-47 | 205.70 | YES |
| IL-6 inhibitors | IL6R | rs79219014 | 1 | 154415675 | T | G | -0.090 | 0.006 | 4.70E-51 | 223.12 | YES |
| IL-6 inhibitors | IL6R | rs9427104 | 1 | 154589232 | T | C | -0.035 | 0.002 | 9.40E-74 | 332.14 | YES |
| IL-6 inhibitors | IL6R | rs61811421 | 1 | 154653951 | T | C | 0.043 | 0.002 | 1.30E-80 | 348.82 | YES |
| IL-6 inhibitors | IL6R | rs10908421 | 1 | 154635885 | T | G | -0.041 | 0.002 | 9.80E-83 | 360.60 | YES |
| IL-6 inhibitors | IL6R | rs12719998 | 1 | 154335417 | G | A | -0.040 | 0.002 | 5.90E-86 | 365.69 | YES |
| IL-6 inhibitors | IL6R | rs77994623 | 1 | 154505106 | T | C | 0.051 | 0.003 | 4.50E-89 | 383.36 | YES |
| IL-6 inhibitors | IL6R | rs56047170 | 1 | 154528053 | A | G | 0.055 | 0.003 | 4.90E-91 | 397.85 | YES |
| IL-6 inhibitors | IL6R | rs56020456 | 1 | 154591882 | T | G | 0.049 | 0.002 | 1.10E-94 | 412.90 | YES |
| IL-6 inhibitors | IL6R | rs4845374 | 1 | 154426947 | A | T | 0.057 | 0.003 | 7.40E-103 | 447.11 | YES |
| IL-6 inhibitors | IL6R | rs12044132 | 1 | 154462360 | T | C | -0.058 | 0.003 | 5.79E-109 | 479.67 | YES |
| IL-6 inhibitors | IL6R | rs1386821 | 1 | 154382049 | G | T | -0.063 | 0.002 | 3.00E-158 | 674.87 | YES |
| IL-6 inhibitors | IL6R | rs12750774 | 1 | 154516477 | A | G | -0.070 | 0.002 | 1.00E-200 | 1096.10 | YES |
| IL-6 inhibitors | IL6R | rs6694817 | 1 | 154401972 | C | T | -0.066 | 0.002 | 1.00E-200 | 1100.16 | YES |
| IL-6 inhibitors | IL6R | rs12568083 | 1 | 154455949 | C | T | -0.070 | 0.002 | 1.00E-200 | 1160.35 | YES |
| JAK inhibitors | JAK1 | rs28425629 | 1 | 65733200 | A | T | -0.031 | 0.006 | 2.00E-08 | 28.45 | YES |
| JAK inhibitors | JAK1 | rs883824 | 1 | 65295857 | A | G | 0.014 | 0.003 | 5.70E-09 | 28.46 | YES |
| JAK inhibitors | JAK1 | rs1408994 | 1 | 65845446 | C | A | -0.023 | 0.004 | 1.10E-08 | 29.31 | YES |
| JAK inhibitors | JAK1 | rs1538345 | 1 | 65542831 | C | T | -0.019 | 0.003 | 5.60E-10 | 31.03 | YES |
| JAK inhibitors | JAK1 | rs11208512 | 1 | 65170405 | C | T | 0.011 | 0.002 | 3.00E-08 | 31.35 | YES |
| JAK inhibitors | JAK1 | rs310245 | 1 | 65306182 | T | C | 0.011 | 0.002 | 1.50E-0 | 31.43 | YES |

| Drug | Target Gene | SNPs | Chr | Position | EA | OA | Beta | SE | P | F-Statistic | Included in Analysis |
|---|---|---|---|---|---|---|---|---|---|---|---|
| | | | | | | | | | 8 | | |
| JAK inhibitors | JAK1 | rs79695825 | 1 | 65502527 | T | C | -0.023 | 0.004 | 2.60E-08 | 32.25 | YES |
| JAK inhibitors | JAK1 | rs12134044 | 1 | 65270549 | G | A | -0.016 | 0.003 | 1.70E-09 | 33.04 | YES |
| JAK inhibitors | JAK1 | rs114280901 | 1 | 65914995 | A | G | 0.035 | 0.006 | 1.50E-09 | 35.84 | YES |
| JAK inhibitors | JAK1 | rs55655800 | 1 | 65900690 | T | G | 0.026 | 0.004 | 4.40E-09 | 35.92 | YES |
| JAK inhibitors | JAK1 | rs11208648 | 1 | 65896612 | G | A | -0.031 | 0.005 | 2.00E-08 | 36.69 | YES |
| JAK inhibitors | JAK1 | rs515394 | 1 | 65729058 | G | A | 0.015 | 0.002 | 1.40E-11 | 41.49 | YES |
| JAK inhibitors | JAK1 | rs61753391 | 1 | 65849887 | A | G | 0.043 | 0.006 | 2.70E-12 | 46.65 | YES |
| JAK inhibitors | JAK1 | rs72921463 | 1 | 65936181 | A | G | 0.020 | 0.003 | 1.20E-10 | 46.71 | YES |
| JAK inhibitors | JAK1 | rs9436276 | 1 | 65836615 | G | A | -0.026 | 0.004 | 2.20E-12 | 47.44 | YES |
| JAK inhibitors | JAK1 | rs12046291 | 1 | 65413953 | G | A | -0.015 | 0.002 | 2.00E-14 | 52.76 | YES |
| JAK inhibitors | JAK1 | rs77451629 | 1 | 65911519 | A | G | 0.043 | 0.006 | 1.80E-14 | 56.05 | YES |
| JAK inhibitors | JAK1 | rs75148473 | 1 | 65884783 | C | T | -0.041 | 0.005 | 7.20E-14 | 57.15 | YES |
| JAK inhibitors | JAK1 | rs111573261 | 1 | 66001886 | G | A | 0.048 | 0.006 | 2.50E-13 | 58.42 | YES |
| JAK inhibitors | JAK1 | rs567910619 | 1 | 65210790 | T | C | -0.071 | 0.008 | 5.10E-17 | 70.78 | YES |
| JAK inhibitors | JAK1 | rs10889495 | 1 | 65155765 | C | T | 0.026 | 0.003 | 5.00E-18 | 73.82 | YES |
| JAK inhibitors | JAK1 | rs11208629 | 1 | 65781901 | A | G | -0.031 | 0.004 | 3.30E-19 | 74.10 | YES |
| JAK inhibitors | JAK1 | rs13374371 | 1 | 65696189 | G | T | 0.026 | 0.003 | 1.80E-20 | 76.99 | YES |
| JAK inhibitors | JAK1 | rs7548127 | 1 | 65686894 | T | G | -0.021 | 0.002 | 2.70E-19 | 80.27 | YES |
| JAK inhibitors | JAK1 | rs12135802 | 1 | 65992140 | G | A | -0.038 | 0.004 | 5.10E-18 | 81.42 | YES |
| JAK inhibitors | JAK1 | rs1570868 | 1 | 65830608 | C | T | 0.018 | 0.002 | 7.20E-23 | 86.99 | YES |
| JAK inhibitors | JAK1 | rs78248443 | 1 | 65155172 | T | C | 0.045 | 0.005 | 2.00E-23 | 96.05 | YES |
| JAK inhibitors | JAK1 | rs4916009 | 1 | 65371418 | T | C | 0.034 | 0.003 | 2.40E-25 | 101.71 | YES |
| JAK inhibitors | JAK1 | rs116509635 | 1 | 65810601 | T | C | 0.075 | 0.007 | 3.30E-27 | 108.55 | YES |
| JAK inhibitors | JAK1 | rs77273543 | 1 | 65976098 | G | C | 0.047 | 0.004 | 2.90E-29 | 114.22 | YES |
| JAK inhibitors | JAK1 | rs2767486 | 1 | 65991203 | G | A | 0.028 | 0.003 | 2.20E-27 | 122.85 | YES |
| JAK inhibitors | JAK1 | rs114328096 | 1 | 65898364 | T | A | -0.100 | 0.008 | 7.60E-42 | 174.60 | YES |
| JAK inhibitors | JAK1 | rs10493377 | 1 | 65879252 | G | A | -0.027 | 0.002 | 6.50E-45 | 187.96 | YES |
| JAK inhibitors | JAK1 | rs113256 | 1 | 65901345 | T | C | -0.103 | 0.007 | 3.20E-5 | 237.17 | YES |

| Drug | Target Gene | SNPs | Chr | Position | EA | OA | Beta | SE | P | F-Statistic | Included in Analysis |
|---|---|---|---|---|---|---|---|---|---|---|---|
| | | 767 | | | | | | | 5 | | |
| JAK inhibitors | JAK1 | rs6672331 | 1 | 65975847 | C | G | 0.142 | 0.005 | 1.60E-159 | 682.46 | YES |
| JAK inhibitors | JAK1 | rs1045895 | 1 | 65897981 | A | G | 0.072 | 0.002 | 1.00E-200 | 1262.48 | YES |
| JAK inhibitors | JAK1 | rs1751492 | 1 | 65992625 | T | C | 0.083 | 0.002 | 1.00E-200 | 1519.74 | YES |
| JAK inhibitors | JAK3 | rs2921 | 19 | 18288810 | G | A | 0.014 | 0.002 | 3.10E-08 | 31.07 | YES |
| JAK inhibitors | JAK3 | rs11554159 | 19 | 18285944 | A | G | -0.014 | 0.002 | 2.60E-10 | 40.10 | YES |
| JAK inhibitors | TYK2 | rs2116942 | 19 | 10334663 | G | T | -0.015 | 0.002 | 2.20E-12 | 50.46 | YES |

SNPs, Single-nucleotide polymorphiss. Chr, chromosome. EA, effect allele. OA, other allele. SE, standard error.

**Table S5.** Sample overlap between exposures and outcomes.

| Outcomes | UK Biobank | Total | Potential overlap |
|---|---|---|---|
| Atrial fibrillation | 395739 | 1,030,836 | 38.00% |
| Coronary artery disease | 0 | 184,305 | 0 |
| Myocardial infarction | 0 | 171,875 | 0 |
| Heart failure | 394,156 | 977,323 | 40.33% |
| Ischemic stroke | 0 | 440,328 | 0 |
| Hypertension | 0 | 218,754 | 0 |
| Total cholesterol | 0 | 187,365 | 0 |
| High-density lipoprotein cholesterol | 0 | 187,167 | 0 |
| Low-density lipoprotein cholesterol | 0 | 173,082 | 0 |
| Triglycerides | 0 | 177,861 | 0 |
| Aortic stenosis | 215,036 | 653,867 | 32.89% |

**Table S6.** Two-sample Mendelian randomization results for DMARD target genes on positive control outcomes.

| Target gene | Rheumatoid arthritis | | | | | |
|---|---|---|---|---|---|---|
| | ebi-a-GCST90018910 | | | ieu-a-832 | | |
| | Effect size estimates | Standard errors | P-values | Effect size estimates | Standard errors | P-values |
| Target gene: PLA2G1B(Sulfasalazine) | **0.285** | **0.070** | **4.18E-05** | 0.115 | 0.125 | 3.61E-01 |
| Target gene: REL(Sulfasalazine) | 0.383 | 1.097 | 7.27E-01 | -0.846 | 2.551 | 7.40E-01 |
| Target gene: RELB(Sulfasalazine) | **0.129** | **0.048** | **7.54E-03** | -0.006 | 0.069 | 9.32E-01 |
| Target gene: RELA(Sulfasalazine) | 0.314 | 0.470 | 5.04E-01 | **2.131** | **0.673** | **1.53E-03** |
| Target gene: ABCG2(Sulfasalazine) | -0.776 | 1.016 | 4.45E-01 | 1.128 | 1.986 | 5.70E-01 |
| Target gene: NFKB1(Sulfasalazine) | -1.703 | 1.452 | 2.41E-01 | 1.161 | 1.643 | 4.80E-01 |
| Target gene: PPP3R2(Cyclosporine) | -0.147 | 0.764 | 8.47E-01 | -0.390 | 1.712 | 8.20E-01 |
| Target gene: PPIF(Cyclosporine) | -0.160 | 0.437 | 7.15E-01 | **-1.466** | **0.697** | **3.55E-02** |
| Target gene: AHR(Leflunomide) | 0.397 | 0.989 | 6.88E-01 | **-3.057** | **1.532** | **4.60E-02** |
| Target gene: TNF/LTA(TNF-alpha inhibitors) | **2.455** | **0.255** | **5.65E-22** | **2.685** | **0.282** | **1.91E-21** |
| Target gene: FCGR1A(TNF-alpha inhibitors) | 1.328 | 0.796 | 9.53E-02 | -1.673 | 1.888 | 3.76E-01 |
| Target gene: C1QA/C1QB/C1QC(TNF-alpha inhibitors) | -0.474 | 1.120 | 6.72E-01 | - | - | - |
| Target gene: IL6R(IL-6 inhibitors) | **0.244** | **0.066** | **2.00E-04** | **0.540** | **0.109** | **6.56E-07** |
| Target gene: CD80(Abatacept) | 0.228 | 0.485 | 6.39E-01 | **-2.721** | **0.776** | **4.57E-04** |
| Target gene: MS4A1(Rituximab) | -0.026 | 0.463 | 9.56E-01 | -0.926 | 0.760 | 2.23E-01 |
| Target gene: JAK1(JAK inhibitors) | 0.068 | 0.069 | 3.23E-01 | **-0.212** | **0.100** | **3.33E-02** |
| Target gene: JAK3(JAK inhibitors) | **2.874** | **0.833** | **5.60E-04** | **2.945** | **1.028** | **4.19E-03** |
| Target gene: TYK2(JAK inhibitors) | 1.731 | 1.143 | 1.30E-01 | **3.339** | **1.140** | **3.40E-03** |

**Table S7 . Directional interpretation of two-sample Mendelian randomization results.**

| Drug | Target gene | Biological pathway | Action | Drug mechanism |
|---|---|---|---|---|
| **IL-6 inhibitors** | IL6R | IL-6 receptor signaling regulates acute-phase responses and systemic inflammatory programs, with downstream involvement in vascular inflammation and atherosclerotic cardiovascular outcomes. | Inhibitor | IL-6 inhibitors block IL6R-mediated signaling, thereby attenuating downstream IL-6 pathway activity. In the present TSMR analyses, genetically proxied IL-6 inhibitions showed negative associations with AF, CAD, MI, HF, IS, and AS, concurrent with a positive association with HDL-C. |
| **Abatacept** | CD80 | T-cell co-stimulation governs adaptive immune activation, influencing vascular inflammation and systemic inflammation relevant to atherosclerotic cardiovascular phenotype. | Antagonist | Abatacept antagonizes CD80, blocking CD28-mediated co-stimulation and suppressing T-cell activation. In the present TSMR analyses, abatacept showed negative associations with TG, HDL-C, and LDL-C, but positive associations with AF, CAD and MI. |
| **Cyclosporine** | PPIF | Mitochondrial stress-response pathways are coupled to inflammation via mPTP regulation, which has significant relevance to inflammation-associated cardiovascular outcomes. | Binder | Cyclosporine binds PPIF, mapping to modulation of mitochondrial stress pathways. In the present TSMR analyses, cyclosporine showed negative associations with HF and TG, but a positive association with IS. |
| **Leflunomide** | AHR | AHR-regulated transcription links ligand/xenobiotic sensing with immune and metabolic regulation, providing a potential mechanistic link to inflammation-associated cardiovascular phenotypes. | Agonist | Leflunomide activates AHR, and in the present TSMR analyses, genetically proxied leflunomide showed negative associations with CAD, MI, and TG, while exhibiting a positive association with HDL-C. |
| **TNF-α inhibitors** | TNF | TNF signaling regulates pro-inflammatory immune responses and systemic inflammation, with downstream relevance to atherosclerotic cardiovascular phenotypes. | Inhibitor | TNF-α inhibitors suppress TNF/TNF-superfamily inflammatory signaling. In the present TSMR analyses, TNF-α inhibition showed negative associations with HF, IS, HTN, and AS. |
| | LTA | TNF-superfamily cytokine signaling contributes to systemic and vascular inflammation, which exerts downstream effects on atherosclerotic risk and vascular dysfunction. | Antibody | |
| **Sulfasalazine** | PLA2G1B | Intestinal phospholipid hydrolysis and lipid metabolism drive | Antagonist | Sulfasalazine inhibits PLA2G1B while activating RELA and RELB, and in the |

| | | | | |
|---|---|---|---|---|
| | | downstream effects on circulating lipid profiles, with lipid-derived inflammatory pathways relevant to atherosclerotic risk. | | present TSMR analyses, sulfasalazine showed negative associations with CAD, MI, IS, TC, LDL-C, and TG, but positive associations with HF,HDL-C and AS. |
| | RELA | Canonical NF-kappaB signaling regulates inflammatory gene transcription and immune responses that are relevant to cardiovascular disease. | Activator | |
| | RELB | Non-canonical NF-kappaB signaling modulates the immune system and inflammatory balance, thereby influencing vascular inflammation and the progression of atherosclerosis. | Activator | |
| **JAK inhibitors** | JAK1 | JAK–STAT signaling operates downstream of cytokine receptors, driving inflammation-associated cardiovascular outcomes. | Inhibitor | JAK inhibitors inhibit JAK1, JAK3, and TYK2, broadly attenuating cytokine-induced JAK–STAT signaling. In the present TSMR analyses, JAK inhibitors showed negative associations with HF, IS, HTN and AS. |
| | JAK3 | JAK3-dependent signaling (notably common γ-chain family) affects lymphocyte activation and inflammatory balance. | Inhibitor | |
| | TYK2 | TYK2 mediates type I interferon, IL-12 and IL-23–related signaling, regulating inflammatory immune responses with potential implications for inflammation-associated cardiovascular outcomes. | Inhibitor | |

An overview of DMARDs, their target genes, implicated biological pathways, and actions, together with a brief description of the drug mechanism. Abbreviations: AF, atrial fibrillation; CAD, coronary artery disease; MI, myocardial infarction; HF, heart failure; IS, ischemic stroke; HTN, hypertension; TC, total cholesterol; HDL-C, high density lipoprotein cholesterol; LDL-C, low-density lipoprotein cholesterol; TG, triglycerides; and AS, aortic stenosis.

**Table S8.** MR analysis with Benjamini-Hochberg false discovery rate (FDR) correction.

| Drug Target | Outcome | Beta | SE | P value | P value (FDR-adjusted) | Significance |
|---|---|---|---|---|---|---|
| PLA2G1B (Sulfasalazine) | AF | 0.003 | 0.049 | 9.46E-01 | 9.62E-01 | |
| PLA2G1B (Sulfasalazine) | CAD | -0.138 | 0.057 | 1.47E-02 | 4.14E-02 | * |
| PLA2G1B (Sulfasalazine) | MI | -0.092 | 0.063 | 1.41E-01 | 2.83E-01 | |
| PLA2G1B (Sulfasalazine) | HF | -0.146 | 0.041 | 3.39E-04 | 2.05E-03 | ** |
| PLA2G1B (Sulfasalazine) | IS | 0.207 | 0.052 | 6.54E-05 | 5.66E-04 | *** |
| PLA2G1B (Sulfasalazine) | HTN | -0.015 | 0.054 | 7.75E-01 | 8.58E-01 | |
| PLA2G1B (Sulfasalazine) | TC | -0.117 | 0.034 | 6.97E-04 | 3.83E-03 | ** |
| PLA2G1B (Sulfasalazine) | HDL-C | -0.126 | 0.035 | 3.36E-04 | 2.05E-03 | ** |
| PLA2G1B (Sulfasalazine) | LDL-C | -0.119 | 0.042 | 4.68E-03 | 1.63E-02 | * |
| PLA2G1B (Sulfasalazine) | TG | -0.017 | 0.025 | 5.03E-01 | 6.62E-01 | |
| PLA2G1B (Sulfasalazine) | AS | -0.324 | 0.076 | 1.84E-05 | 2.03E-04 | *** |
| RELB (Sulfasalazine) | AF | -0.029 | 0.027 | 2.81E-01 | 4.25E-01 | |
| RELB (Sulfasalazine) | CAD | -0.318 | 0.058 | 3.30E-08 | 5.70E-07 | *** |
| RELB (Sulfasalazine) | MI | -0.320 | 0.058 | 4.55E-08 | 6.89E-07 | *** |
| RELB (Sulfasalazine) | HF | 0.077 | 0.031 | 1.20E-02 | 3.47E-02 | * |
| RELB (Sulfasalazine) | IS | -0.082 | 0.040 | 3.91E-02 | 9.11E-02 | |
| RELB (Sulfasalazine) | HTN | -0.041 | 0.043 | 3.45E-01 | 5.09E-01 | |
| RELB (Sulfasalazine) | TC | -1.191 | 0.150 | 1.81E-15 | 2.20E-13 | *** |
| RELB (Sulfasalazine) | HDL-C | 0.352 | 0.047 | 5.11E-14 | 3.09E-12 | *** |
| RELB (Sulfasalazine) | LDL-C | -1.538 | 0.241 | 1.77E-10 | 7.13E-09 | *** |
| RELB (Sulfasalazine) | TG | -0.186 | 0.077 | 1.59E-02 | 4.37E-02 | * |
| RELB (Sulfasalazine) | AS | 0.012 | 0.055 | 8.31E-01 | 8.99E-01 | |
| RELA (Sulfasalazine) | AF | 0.602 | 0.236 | 1.07E-02 | 3.20E-02 | * |
| RELA (Sulfasalazine) | CAD | 1.352 | 0.494 | 6.26E-03 | 2.05E-02 | * |
| RELA (Sulfasalazine) | MI | 1.565 | 0.393 | 6.81E-05 | 5.66E-04 | *** |
| RELA (Sulfasalazine) | HF | 0.799 | 0.278 | 3.99E-03 | 1.46E-02 | * |
| RELA (Sulfasalazine) | IS | -0.116 | 0.413 | 7.80E-01 | 8.58E-01 | |
| RELA (Sulfasalazine) | HTN | 0.997 | 0.344 | 3.72E-03 | 1.46E-02 | * |
| RELA (Sulfasalazine) | TC | -0.415 | 0.297 | 1.62E-01 | 3.02E-01 | |
| RELA (Sulfasalazine) | HDL-C | -0.058 | 0.273 | 8.32E-01 | 8.99E-01 | |
| RELA (Sulfasalazine) | LDL-C | -0.614 | 0.269 | 2.23E-02 | 5.75E-02 | |
| RELA (Sulfasalazine) | TG | 0.470 | 0.273 | 8.48E-02 | 1.83E-01 | |
| RELA (Sulfasalazine) | AS | -0.546 | 0.497 | 2.72E-01 | 4.17E-01 | |
| TNF/LTA (TNF-alpha inhibitors) | AF | 0.258 | 0.183 | 1.59E-01 | 3.01E-01 | |
| TNF/LTA (TNF-alpha inhibitors) | CAD | 0.661 | 0.492 | 1.79E-01 | 3.19E-01 | |
| TNF/LTA (TNF-alpha inhibitors) | MI | 0.278 | 0.355 | 4.35E-01 | 5.97E-01 | |
| TNF/LTA (TNF-alpha inhibitors) | HF | 0.506 | 0.173 | 3.43E-03 | 1.43E-02 | * |
| TNF/LTA (TNF-alpha inhibitors) | IS | 0.715 | 0.226 | 1.53E-03 | 7.71E-03 | ** |
| TNF/LTA (TNF-alpha inhibitors) | HTN | 0.942 | 0.243 | 1.04E-04 | 7.88E-04 | *** |

| | | | | | | |
|---|---|---|---|---|---|---|
| TNF/LTA (TNF-alpha inhibitors) | TC | -0.047 | 0.431 | 9.14E-01 | 9.60E-01 | |
| TNF/LTA (TNF-alpha inhibitors) | HDL-C | -0.093 | 0.277 | 7.38E-01 | 8.34E-01 | |
| TNF/LTA (TNF-alpha inhibitors) | LDL-C | 0.032 | 0.359 | 9.28E-01 | 9.60E-01 | |
| TNF/LTA (TNF-alpha inhibitors) | TG | -0.110 | 0.188 | 5.58E-01 | 7.03E-01 | |
| TNF/LTA (TNF-alpha inhibitors) | AS | 0.786 | 0.273 | 3.92E-03 | 1.46E-02 | * |
| PPIF (Cyclosporine) | AF | 0.360 | 0.534 | 5.01E-01 | 6.62E-01 | |
| PPIF (Cyclosporine) | CAD | 0.573 | 0.319 | 7.19E-02 | 1.61E-01 | |
| PPIF (Cyclosporine) | MI | 0.511 | 0.410 | 2.13E-01 | 3.53E-01 | |
| PPIF (Cyclosporine) | HF | -0.551 | 0.265 | 3.74E-02 | 9.05E-02 | |
| PPIF (Cyclosporine) | IS | 1.201 | 0.345 | 5.01E-04 | 2.89E-03 | ** |
| PPIF (Cyclosporine) | HTN | -0.186 | 0.316 | 5.56E-01 | 7.03E-01 | |
| PPIF (Cyclosporine) | TC | -0.258 | 0.225 | 2.50E-01 | 3.94E-01 | |
| PPIF (Cyclosporine) | HDL-C | -0.132 | 0.190 | 4.89E-01 | 6.57E-01 | |
| PPIF (Cyclosporine) | LDL-C | 0.307 | 0.232 | 1.87E-01 | 3.19E-01 | |
| PPIF (Cyclosporine) | TG | -0.515 | 0.186 | 5.49E-03 | 1.85E-02 | * |
| PPIF (Cyclosporine) | AS | 0.036 | 0.497 | 9.42E-01 | 9.62E-01 | |
| IL6R (IL-6 inhibitors) | AF | 0.257 | 0.053 | 1.47E-06 | 1.78E-05 | *** |
| IL6R (IL-6 inhibitors) | CAD | 0.324 | 0.062 | 1.47E-07 | 1.98E-06 | *** |
| IL6R (IL-6 inhibitors) | MI | 0.237 | 0.066 | 3.07E-04 | 2.05E-03 | ** |
| IL6R (IL-6 inhibitors) | HF | 0.127 | 0.056 | 2.45E-02 | 6.04E-02 | |
| IL6R (IL-6 inhibitors) | IS | 0.293 | 0.050 | 5.45E-09 | 1.10E-07 | *** |
| IL6R (IL-6 inhibitors) | HTN | -0.042 | 0.052 | 4.17E-01 | 5.93E-01 | |
| IL6R (IL-6 inhibitors) | TC | -0.033 | 0.030 | 2.67E-01 | 4.14E-01 | |
| IL6R (IL-6 inhibitors) | HDL-C | -0.073 | 0.031 | 1.68E-02 | 4.50E-02 | * |
| IL6R (IL-6 inhibitors) | LDL-C | -0.003 | 0.030 | 9.28E-01 | 9.60E-01 | |
| IL6R (IL-6 inhibitors) | TG | -0.046 | 0.029 | 1.19E-01 | 2.43E-01 | |
| IL6R (IL-6 inhibitors) | AS | 0.559 | 0.091 | 7.69E-10 | 2.33E-08 | *** |
| AHR (Leflunomide) | AF | -0.711 | 0.517 | 1.69E-01 | 3.09E-01 | |
| AHR (Leflunomide) | CAD | -2.045 | 0.724 | 4.72E-03 | 1.63E-02 | * |
| AHR (Leflunomide) | MI | -2.055 | 0.807 | 1.09E-02 | 3.20E-02 | * |
| AHR (Leflunomide) | HF | -0.300 | 0.614 | 6.26E-01 | 7.46E-01 | |
| AHR (Leflunomide) | IS | 0.382 | 0.794 | 6.30E-01 | 7.46E-01 | |
| AHR (Leflunomide) | HTN | -1.258 | 0.726 | 8.33E-02 | 1.83E-01 | |
| AHR (Leflunomide) | TC | -0.374 | 0.419 | 3.72E-01 | 5.42E-01 | |
| AHR (Leflunomide) | HDL-C | 1.228 | 0.397 | 1.97E-03 | 9.18E-03 | ** |
| AHR (Leflunomide) | LDL-C | -0.614 | 0.427 | 1.50E-01 | 2.93E-01 | |
| AHR (Leflunomide) | TG | -1.101 | 0.382 | 3.95E-03 | 1.46E-02 | * |
| AHR (Leflunomide) | AS | 0.052 | 1.089 | 9.62E-01 | 9.70E-01 | |
| CD80 (Abatacept) | AF | -0.651 | 0.252 | 9.67E-03 | 3.00E-02 | * |
| CD80 (Abatacept) | CAD | -0.704 | 0.340 | 3.83E-02 | 9.09E-02 | |
| CD80 (Abatacept) | MI | -0.768 | 0.380 | 4.32E-02 | 9.87E-02 | |
| CD80 (Abatacept) | HF | -0.550 | 0.334 | 9.98E-02 | 2.08E-01 | |
| CD80 (Abatacept) | IS | -0.538 | 0.379 | 1.56E-01 | 2.99E-01 | |
| CD80 (Abatacept) | HTN | 0.004 | 0.343 | 9.90E-01 | 9.90E-01 | |

| | | | | | | |
|---|---|---|---|---|---|---|
| CD80 (Abatacept) | TC | 1.127 | 0.186 | 1.48E-09 | 3.58E-08 | *** |
| CD80 (Abatacept) | HDL-C | 1.072 | 0.270 | 7.02E-05 | 5.66E-04 | *** |
| CD80 (Abatacept) | LDL-C | 0.787 | 0.191 | 3.77E-05 | 3.80E-04 | *** |
| CD80 (Abatacept) | TG | 0.060 | 0.173 | 7.31E-01 | 8.34E-01 | |
| CD80 (Abatacept) | AS | 0.413 | 0.871 | 6.35E-01 | 7.46E-01 | |
| JAK1 (JAK inhibitors) | AF | -0.055 | 0.047 | 2.43E-01 | 3.93E-01 | |
| JAK1 (JAK inhibitors) | CAD | -0.010 | 0.069 | 8.84E-01 | 9.47E-01 | |
| JAK1 (JAK inhibitors) | MI | 0.040 | 0.077 | 6.03E-01 | 7.30E-01 | |
| JAK1 (JAK inhibitors) | HF | -0.077 | 0.059 | 1.95E-01 | 3.28E-01 | |
| JAK1 (JAK inhibitors) | IS | 0.097 | 0.073 | 1.85E-01 | 3.19E-01 | |
| JAK1 (JAK inhibitors) | HTN | -0.254 | 0.085 | 2.80E-03 | 1.25E-02 | * |
| JAK1 (JAK inhibitors) | TC | -0.051 | 0.050 | 3.06E-01 | 4.58E-01 | |
| JAK1 (JAK inhibitors) | HDL-C | -0.038 | 0.051 | 4.59E-01 | 6.25E-01 | |
| JAK1 (JAK inhibitors) | LDL-C | -0.024 | 0.044 | 5.90E-01 | 7.21E-01 | |
| JAK1 (JAK inhibitors) | TG | 0.012 | 0.039 | 7.54E-01 | 8.45E-01 | |
| JAK1 (JAK inhibitors) | AS | 0.060 | 0.104 | 5.65E-01 | 7.05E-01 | |
| JAK3 (JAK inhibitors) | AF | 0.149 | 0.394 | 7.06E-01 | 8.14E-01 | |
| JAK3 (JAK inhibitors) | CAD | 1.672 | 0.567 | 3.19E-03 | 1.38E-02 | * |
| JAK3 (JAK inhibitors) | MI | 1.691 | 0.628 | 7.05E-03 | 2.24E-02 | * |
| JAK3 (JAK inhibitors) | HF | -0.193 | 0.462 | 6.77E-01 | 7.88E-01 | |
| JAK3 (JAK inhibitors) | IS | -0.392 | 0.614 | 5.23E-01 | 6.80E-01 | |
| JAK3 (JAK inhibitors) | HTN | 0.654 | 0.813 | 4.21E-01 | 5.93E-01 | |
| JAK3 (JAK inhibitors) | TC | -1.103 | 0.299 | 2.23E-04 | 1.59E-03 | ** |
| JAK3 (JAK inhibitors) | HDL-C | -0.320 | 0.279 | 2.51E-01 | 3.94E-01 | |
| JAK3 (JAK inhibitors) | LDL-C | -0.956 | 0.306 | 1.79E-03 | 8.66E-03 | ** |
| JAK3 (JAK inhibitors) | TG | -0.214 | 0.274 | 4.34E-01 | 5.97E-01 | |
| JAK3 (JAK inhibitors) | AS | -0.487 | 0.903 | 5.89E-01 | 7.21E-01 | |
| TYK2 (JAK inhibitors) | AF | 0.931 | 0.643 | 1.48E-01 | 2.93E-01 | |
| TYK2 (JAK inhibitors) | CAD | 0.795 | 0.681 | 2.43E-01 | 3.93E-01 | |
| TYK2 (JAK inhibitors) | MI | 0.649 | 0.756 | 3.91E-01 | 5.64E-01 | |
| TYK2 (JAK inhibitors) | HF | 1.074 | 0.814 | 1.87E-01 | 3.19E-01 | |
| TYK2 (JAK inhibitors) | IS | 0.431 | 0.718 | 5.49E-01 | 7.03E-01 | |
| TYK2 (JAK inhibitors) | HTN | 1.430 | 0.630 | 2.31E-02 | 5.82E-02 | |
| TYK2 (JAK inhibitors) | TC | 0.431 | 0.253 | 8.86E-02 | 1.88E-01 | |
| TYK2 (JAK inhibitors) | HDL-C | 0.027 | 0.246 | 9.12E-01 | 9.60E-01 | |
| TYK2 (JAK inhibitors) | LDL-C | 0.630 | 0.267 | 1.83E-02 | 4.82E-02 | * |
| TYK2 (JAK inhibitors) | TG | 0.780 | 0.239 | 1.13E-03 | 5.92E-03 | ** |
| TYK2 (JAK inhibitors) | AS | 1.550 | 1.170 | 1.85E-01 | 3.19E-01 | |

Statistical significance after FDR correction is indicated as follows: *** forFDR-adjusted P < 0.001, ** for FDR-adjusted P < 0.01, * for FDR-adjusted P < 0.05, and no symbol indicates non-significance. Abbreviations: SE, standard error; AF, atrial fibrillation; CAD, coronary artery disease; MI, myocardial infarction; HF, heart failure; IS, ischemic stroke; HTN, hypertension; TC, total cholesterol; HDL-C, high density lipoprotein cholesterol; LDL-C, low-density lipoprotein cholesterol; TG, triglycerides; AS, aortic stenosis.

**Table S9.** TSMR results for drug targets on cardiovascular disease outcomes using different methods.

| Drug Target | Outcome | method | OR(95%CI) |
|---|---|---|---|
| Target gene: RELB (Sulfasalazine) | AF | MR Egger | 1.03(0.96-1.11) |
| Target gene: RELB (Sulfasalazine) | AF | Weighted median | 1.05(0.98-1.13) |
| Target gene: RELB (Sulfasalazine) | AF | Inverse variance weighted (multiplicative random effects) | 0.97(0.92-1.02) |
| Target gene: RELB (Sulfasalazine) | AF | Inverse variance weighted (fixed effects) | 0.97(0.93-1.02) |
| Target gene: RELB (Sulfasalazine) | AF | Simple median | 0.91(0.82-1.01) |
| Target gene: RELB (Sulfasalazine) | CAD | MR Egger | 0.67(0.57-0.79) |
| Target gene: RELB (Sulfasalazine) | CAD | Weighted median | 0.77(0.68-0.87) |
| Target gene: RELB (Sulfasalazine) | CAD | Inverse variance weighted (multiplicative random effects) | 0.73(0.65-0.81) |
| Target gene: RELB (Sulfasalazine) | CAD | Inverse variance weighted (fixed effects) | 0.73(0.68-0.78) |
| Target gene: RELB (Sulfasalazine) | CAD | Simple median | 0.75(0.63-0.88) |
| Target gene: RELB (Sulfasalazine) | MI | MR Egger | 0.64(0.54-0.75) |
| Target gene: RELB (Sulfasalazine) | MI | Weighted median | 0.75(0.66-0.86) |
| Target gene: RELB (Sulfasalazine) | MI | Inverse variance weighted (multiplicative random effects) | 0.73(0.65-0.81) |
| Target gene: RELB (Sulfasalazine) | MI | Inverse variance weighted (fixed effects) | 0.73(0.67-0.79) |
| Target gene: RELB (Sulfasalazine) | MI | Simple median | 0.77(0.64-0.91) |
| Target gene: RELB (Sulfasalazine) | HF | MR Egger | 1.02(0.94-1.11) |
| Target gene: RELB (Sulfasalazine) | HF | Weighted median | 1.08(0.99-1.18) |
| Target gene: RELB (Sulfasalazine) | HF | Inverse variance weighted (multiplicative random effects) | 1.08(1.02-1.15) |
| Target gene: RELB (Sulfasalazine) | HF | Inverse variance weighted (fixed effects) | 1.08(1.02-1.14) |
| Target gene: RELB (Sulfasalazine) | HF | Simple median | 1.16(1.02-1.31) |
| Target gene: RELB (Sulfasalazine) | IS | MR Egger | 0.99(0.89-1.11) |
| Target gene: RELB (Sulfasalazine) | IS | Weighted median | 1.00(0.90-1.12) |
| Target gene: RELB (Sulfasalazine) | IS | Inverse variance weighted (multiplicative random effects) | 0.92(0.85-1.00) |
| Target gene: RELB (Sulfasalazine) | IS | Inverse variance weighted (fixed effects) | 0.92(0.85-0.99) |
| Target gene: RELB (Sulfasalazine) | IS | Simple median | 0.92(0.78-1.09) |
| Target gene: RELB (Sulfasalazine) | HTN | MR Egger | 1.02(0.90-1.14) |
| Target gene: RELB (Sulfasalazine) | HTN | Weighted median | 1.02(0.92-1.12) |
| Target gene: RELB (Sulfasalazine) | HTN | Inverse variance weighted (multiplicative random effects) | 0.96(0.88-1.04) |
| Target gene: RELB (Sulfasalazine) | HTN | Inverse variance weighted (fixed effects) | 0.96(0.90-1.02) |
| Target gene: RELB (Sulfasalazine) | HTN | Simple median | 0.98(0.84-1.13) |
| Target gene: RELB (Sulfasalazine) | TC | MR Egger | 0.30(0.19-0.48) |
| Target gene: RELB (Sulfasalazine) | TC | Weighted median | 0.35(0.32-0.38) |
| Target gene: RELB (Sulfasalazine) | TC | Inverse variance weighted (multiplicative random effects) | 0.30(0.23-0.41) |
| Target gene: RELB (Sulfasalazine) | TC | Inverse variance weighted (fixed effects) | 0.30(0.29-0.32) |
| Target gene: RELB (Sulfasalazine) | TC | Simple median | 0.42(0.35-0.51) |
| Target gene: RELB (Sulfasalazine) | HDL-C | MR Egger | 1.53(1.33-1.77) |
| Target gene: RELB (Sulfasalazine) | HDL-C | Weighted median | 1.38(1.30-1.47) |
| Target gene: RELB (Sulfasalazine) | HDL-C | Inverse variance weighted (multiplicative random effects) | 1.42(1.30-1.56) |
| Target gene: RELB (Sulfasalazine) | HDL-C | Inverse variance weighted (fixed effects) | 1.42(1.36-1.49) |

| | | | |
|---|---|---|---|
| Target gene: RELB (Sulfasalazine) | HDL-C | Simple median | 1.38(1.27-1.51) |
| Target gene: RELB (Sulfasalazine) | LDL-C | MR Egger | 0.20(0.10-0.44) |
| Target gene: RELB (Sulfasalazine) | LDL-C | Weighted median | 0.31(0.28-0.34) |
| Target gene: RELB (Sulfasalazine) | LDL-C | Inverse variance weighted (multiplicative random effects) | 0.21(0.13-0.34) |
| Target gene: RELB (Sulfasalazine) | LDL-C | Inverse variance weighted (fixed effects) | 0.21(0.20-0.23) |
| Target gene: RELB (Sulfasalazine) | LDL-C | Simple median | 0.33(0.27-0.39) |
| Target gene: RELB (Sulfasalazine) | TG | MR Egger | 0.79(0.62-1.00) |
| Target gene: RELB (Sulfasalazine) | TG | Weighted median | 0.73(0.67-0.81) |
| Target gene: RELB (Sulfasalazine) | TG | Inverse variance weighted (multiplicative random effects) | 0.83(0.71-0.97) |
| Target gene: RELB (Sulfasalazine) | TG | Inverse variance weighted (fixed effects) | 0.83(0.79-0.87) |
| Target gene: RELB (Sulfasalazine) | TG | Simple median | 0.82(0.72-0.93) |
| Target gene: RELB (Sulfasalazine) | AS | MR Egger | 0.94(0.81-1.10) |
| Target gene: RELB (Sulfasalazine) | AS | Weighted median | 1.05(0.89-1.25) |
| Target gene: RELB (Sulfasalazine) | AS | Inverse variance weighted (multiplicative random effects) | 1.01(0.91-1.13) |
| Target gene: RELB (Sulfasalazine) | AS | Inverse variance weighted (fixed effects) | 1.01(0.91-1.13) |
| Target gene: RELB (Sulfasalazine) | AS | Simple median | 1.02(0.83-1.26) |
| Target gene: RELA (Sulfasalazine) | AF | MR Egger | 4.13(0.18-92.60) |
| Target gene: RELA (Sulfasalazine) | AF | Weighted median | 1.76(1.03-3.00) |
| Target gene: RELA (Sulfasalazine) | AF | Inverse variance weighted (multiplicative random effects) | 1.83(1.52-2.19) |
| Target gene: RELA (Sulfasalazine) | AF | Inverse variance weighted (fixed effects) | 1.83(1.15-2.90) |
| Target gene: RELA (Sulfasalazine) | AF | Simple median | 1.74(0.95-3.19) |
| Target gene: RELA (Sulfasalazine) | CAD | MR Egger | 6.44(0.00-10514.70) |
| Target gene: RELA (Sulfasalazine) | CAD | Weighted median | 2.75(1.08-7.02) |
| Target gene: RELA (Sulfasalazine) | CAD | Inverse variance weighted (multiplicative random effects) | 3.86(1.47-10.19) |
| Target gene: RELA (Sulfasalazine) | CAD | Inverse variance weighted (fixed effects) | 3.86(2.01-7.42) |
| Target gene: RELA (Sulfasalazine) | CAD | Simple median | 2.77(1.04-7.33) |
| Target gene: RELA (Sulfasalazine) | MI | MR Egger | 8.21(0.02-2848.95) |
| Target gene: RELA (Sulfasalazine) | MI | Weighted median | 4.19(1.51-11.64) |
| Target gene: RELA (Sulfasalazine) | MI | Inverse variance weighted (multiplicative random effects) | 4.78(2.21-10.34) |
| Target gene: RELA (Sulfasalazine) | MI | Inverse variance weighted (fixed effects) | 4.78(2.33-9.83) |
| Target gene: RELA (Sulfasalazine) | MI | Simple median | 4.20(1.49-11.82) |
| Target gene: RELA (Sulfasalazine) | HF | MR Egger | 2.45(0.05-123.38) |
| Target gene: RELA (Sulfasalazine) | HF | Weighted median | 2.07(1.02-4.18) |
| Target gene: RELA (Sulfasalazine) | HF | Inverse variance weighted (multiplicative random effects) | 2.22(1.34-3.68) |
| Target gene: RELA (Sulfasalazine) | HF | Inverse variance weighted (fixed effects) | 2.22(1.29-3.83) |
| Target gene: RELA (Sulfasalazine) | HF | Simple median | 2.05(1.01-4.13) |
| Target gene: RELA (Sulfasalazine) | IS | MR Egger | 2.41(0.00-1167.51) |
| Target gene: RELA (Sulfasalazine) | IS | Weighted median | 1.11(0.46-2.67) |
| Target gene: RELA (Sulfasalazine) | IS | Inverse variance weighted (multiplicative random effects) | 0.89(0.40-2.00) |
| Target gene: RELA (Sulfasalazine) | IS | Inverse variance weighted (fixed effects) | 0.89(0.44-1.79) |
| Target gene: RELA (Sulfasalazine) | IS | Simple median | 1.01(0.40-2.53) |
| Target gene: RELA (Sulfasalazine) | HTN | MR Egger | 0.10(0.00-6.67) |
| Target gene: RELA (Sulfasalazine) | HTN | Weighted median | 2.18(0.95-5.00) |
| Target gene: RELA (Sulfasalazine) | HTN | Inverse variance weighted (multiplicative random effects) | 2.71(1.38-5.31) |

| | | | |
|---|---|---|---|
| Target gene: RELA (Sulfasalazine) | HTN | Inverse variance weighted (fixed effects) | 2.71(1.44-5.08) |
| Target gene: RELA (Sulfasalazine) | HTN | Simple median | 2.59(1.08-6.23) |
| Target gene: RELA (Sulfasalazine) | TC | MR Egger | 19.98(1.77-224.94) |
| Target gene: RELA (Sulfasalazine) | TC | Weighted median | 0.69(0.43-1.09) |
| Target gene: RELA (Sulfasalazine) | TC | Inverse variance weighted (multiplicative random effects) | 0.66(0.37-1.18) |
| Target gene: RELA (Sulfasalazine) | TC | Inverse variance weighted (fixed effects) | 0.66(0.46-0.95) |
| Target gene: RELA (Sulfasalazine) | TC | Simple median | 0.47(0.26-0.85) |
| Target gene: RELA (Sulfasalazine) | HDL-C | MR Egger | 1.69(0.03-97.36) |
| Target gene: RELA (Sulfasalazine) | HDL-C | Weighted median | 0.87(0.54-1.40) |
| Target gene: RELA (Sulfasalazine) | HDL-C | Inverse variance weighted (multiplicative random effects) | 0.94(0.55-1.61) |
| Target gene: RELA (Sulfasalazine) | HDL-C | Inverse variance weighted (fixed effects) | 0.94(0.67-1.33) |
| Target gene: RELA (Sulfasalazine) | HDL-C | Simple median | 0.73(0.42-1.27) |
| Target gene: RELA (Sulfasalazine) | LDL-C | MR Egger | 11.57(0.98-136.31) |
| Target gene: RELA (Sulfasalazine) | LDL-C | Weighted median | 0.54(0.33-0.88) |
| Target gene: RELA (Sulfasalazine) | LDL-C | Inverse variance weighted (multiplicative random effects) | 0.54(0.32-0.92) |
| Target gene: RELA (Sulfasalazine) | LDL-C | Inverse variance weighted (fixed effects) | 0.54(0.37-0.78) |
| Target gene: RELA (Sulfasalazine) | LDL-C | Simple median | 0.39(0.22-0.71) |
| Target gene: RELA (Sulfasalazine) | TG | MR Egger | 21.65(1.23-380.00) |
| Target gene: RELA (Sulfasalazine) | TG | Weighted median | 1.18(0.73-1.90) |
| Target gene: RELA (Sulfasalazine) | TG | Inverse variance weighted (multiplicative random effects) | 1.60(0.94-2.73) |
| Target gene: RELA (Sulfasalazine) | TG | Inverse variance weighted (fixed effects) | 1.60(1.14-2.23) |
| Target gene: RELA (Sulfasalazine) | TG | Simple median | 1.24(0.73-2.10) |
| Target gene: RELA (Sulfasalazine) | AS | MR Egger | 0.66(0.00-484.13) |
| Target gene: RELA (Sulfasalazine) | AS | Weighted median | 0.59(0.18-1.91) |
| Target gene: RELA (Sulfasalazine) | AS | Inverse variance weighted (multiplicative random effects) | 0.58(0.42-0.80) |
| Target gene: RELA (Sulfasalazine) | AS | Inverse variance weighted (fixed effects) | 0.58(0.22-1.53) |
| Target gene: RELA (Sulfasalazine) | AS | Simple median | 0.60(0.17-2.08) |
| Target gene: PLA2G1B (Sulfasalazine) | AF | MR Egger | 1.00(0.83-1.21) |
| Target gene: PLA2G1B (Sulfasalazine) | AF | Weighted median | 0.89(0.79-1.00) |
| Target gene: PLA2G1B (Sulfasalazine) | AF | Inverse variance weighted (multiplicative random effects) | 1.00(0.91-1.10) |
| Target gene: PLA2G1B (Sulfasalazine) | AF | Inverse variance weighted (fixed effects) | 1.00(0.94-1.07) |
| Target gene: PLA2G1B (Sulfasalazine) | AF | Simple median | 1.00(0.87-1.14) |
| Target gene: PLA2G1B (Sulfasalazine) | CAD | MR Egger | 0.81(0.65-1.00) |
| Target gene: PLA2G1B (Sulfasalazine) | CAD | Weighted median | 0.81(0.70-0.94) |
| Target gene: PLA2G1B (Sulfasalazine) | CAD | Inverse variance weighted (multiplicative random effects) | 0.87(0.78-0.97) |
| Target gene: PLA2G1B (Sulfasalazine) | CAD | Inverse variance weighted (fixed effects) | 0.87(0.79-0.96) |
| Target gene: PLA2G1B (Sulfasalazine) | CAD | Simple median | 0.94(0.79-1.12) |
| Target gene: PLA2G1B (Sulfasalazine) | MI | MR Egger | 0.84(0.66-1.06) |
| Target gene: PLA2G1B (Sulfasalazine) | MI | Weighted median | 0.86(0.73-1.01) |
| Target gene: PLA2G1B (Sulfasalazine) | MI | Inverse variance weighted (multiplicative random effects) | 0.91(0.81-1.03) |
| Target gene: PLA2G1B (Sulfasalazine) | MI | Inverse variance weighted (fixed effects) | 0.91(0.82-1.01) |
| Target gene: PLA2G1B (Sulfasalazine) | MI | Simple median | 0.91(0.75-1.11) |
| Target gene: PLA2G1B (Sulfasalazine) | HF | MR Egger | 0.94(0.81-1.10) |
| Target gene: PLA2G1B (Sulfasalazine) | HF | Weighted median | 0.90(0.80-1.01) |

| | | | |
|---|---|---|---|
| Target gene: PLA2G1B (Sulfasalazine) | HF | Inverse variance weighted (multiplicative random effects) | 0.86(0.80-0.94) |
| Target gene: PLA2G1B (Sulfasalazine) | HF | Inverse variance weighted (fixed effects) | 0.86(0.80-0.94) |
| Target gene: PLA2G1B (Sulfasalazine) | HF | Simple median | 0.87(0.76-1.00) |
| Target gene: PLA2G1B (Sulfasalazine) | IS | MR Egger | 1.10(0.91-1.35) |
| Target gene: PLA2G1B (Sulfasalazine) | IS | Weighted median | 1.28(1.09-1.50) |
| Target gene: PLA2G1B (Sulfasalazine) | IS | Inverse variance weighted (multiplicative random effects) | 1.23(1.11-1.36) |
| Target gene: PLA2G1B (Sulfasalazine) | IS | Inverse variance weighted (fixed effects) | 1.23(1.11-1.36) |
| Target gene: PLA2G1B (Sulfasalazine) | IS | Simple median | 1.16(0.97-1.38) |
| Target gene: PLA2G1B (Sulfasalazine) | HTN | MR Egger | 0.92(0.75-1.14) |
| Target gene: PLA2G1B (Sulfasalazine) | HTN | Weighted median | 0.98(0.86-1.12) |
| Target gene: PLA2G1B (Sulfasalazine) | HTN | Inverse variance weighted (multiplicative random effects) | 0.98(0.89-1.09) |
| Target gene: PLA2G1B (Sulfasalazine) | HTN | Inverse variance weighted (fixed effects) | 0.98(0.90-1.07) |
| Target gene: PLA2G1B (Sulfasalazine) | HTN | Simple median | 1.04(0.89-1.22) |
| Target gene: PLA2G1B (Sulfasalazine) | TC | MR Egger | 0.93(0.82-1.07) |
| Target gene: PLA2G1B (Sulfasalazine) | TC | Weighted median | 0.86(0.80-0.93) |
| Target gene: PLA2G1B (Sulfasalazine) | TC | Inverse variance weighted (multiplicative random effects) | 0.89(0.83-0.95) |
| Target gene: PLA2G1B (Sulfasalazine) | TC | Inverse variance weighted (fixed effects) | 0.89(0.84-0.94) |
| Target gene: PLA2G1B (Sulfasalazine) | TC | Simple median | 0.84(0.77-0.93) |
| Target gene: PLA2G1B (Sulfasalazine) | HDL-C | MR Egger | 0.93(0.81-1.07) |
| Target gene: PLA2G1B (Sulfasalazine) | HDL-C | Weighted median | 0.89(0.83-0.97) |
| Target gene: PLA2G1B (Sulfasalazine) | HDL-C | Inverse variance weighted (multiplicative random effects) | 0.88(0.82-0.94) |
| Target gene: PLA2G1B (Sulfasalazine) | HDL-C | Inverse variance weighted (fixed effects) | 0.88(0.84-0.93) |
| Target gene: PLA2G1B (Sulfasalazine) | HDL-C | Simple median | 0.86(0.78-0.94) |
| Target gene: PLA2G1B (Sulfasalazine) | LDL-C | MR Egger | 0.88(0.75-1.04) |
| Target gene: PLA2G1B (Sulfasalazine) | LDL-C | Weighted median | 0.82(0.75-0.89) |
| Target gene: PLA2G1B (Sulfasalazine) | LDL-C | Inverse variance weighted (multiplicative random effects) | 0.89(0.82-0.96) |
| Target gene: PLA2G1B (Sulfasalazine) | LDL-C | Inverse variance weighted (fixed effects) | 0.89(0.84-0.94) |
| Target gene: PLA2G1B (Sulfasalazine) | LDL-C | Simple median | 0.83(0.74-0.93) |
| Target gene: PLA2G1B (Sulfasalazine) | TG | MR Egger | 0.96(0.87-1.06) |
| Target gene: PLA2G1B (Sulfasalazine) | TG | Weighted median | 0.96(0.89-1.03) |
| Target gene: PLA2G1B (Sulfasalazine) | TG | Inverse variance weighted (multiplicative random effects) | 0.98(0.94-1.03) |
| Target gene: PLA2G1B (Sulfasalazine) | TG | Inverse variance weighted (fixed effects) | 0.98(0.94-1.03) |
| Target gene: PLA2G1B (Sulfasalazine) | TG | Simple median | 1.01(0.93-1.09) |
| Target gene: PLA2G1B (Sulfasalazine) | AS | MR Egger | 0.71(0.53-0.94) |
| Target gene: PLA2G1B (Sulfasalazine) | AS | Weighted median | 0.70(0.56-0.87) |
| Target gene: PLA2G1B (Sulfasalazine) | AS | Inverse variance weighted (multiplicative random effects) | 0.72(0.62-0.84) |
| Target gene: PLA2G1B (Sulfasalazine) | AS | Inverse variance weighted (fixed effects) | 0.72(0.63-0.83) |
| Target gene: PLA2G1B (Sulfasalazine) | AS | Simple median | 0.81(0.64-1.02) |
| Target gene: IL6R (IL-6 inhibitors) | AF | MR Egger | 1.39(1.05-1.84) |
| Target gene: IL6R (IL-6 inhibitors) | AF | Weighted median | 1.24(1.10-1.40) |
| Target gene: IL6R (IL-6 inhibitors) | AF | Inverse variance weighted (multiplicative random effects) | 1.29(1.16-1.44) |
| Target gene: IL6R (IL-6 inhibitors) | AF | Inverse variance weighted (fixed effects) | 1.29(1.21-1.38) |
| Target gene: IL6R (IL-6 inhibitors) | AF | Simple median | 1.22(1.08-1.39) |
| Target gene: IL6R (IL-6 inhibitors) | CAD | MR Egger | 1.68(1.22-2.32) |

| | | | |
|---|---|---|---|
| Target gene: IL6R (IL-6 inhibitors) | CAD | Weighted median | 1.30(1.10-1.54) |
| Target gene: IL6R (IL-6 inhibitors) | CAD | Inverse variance weighted (multiplicative random effects) | 1.38(1.23-1.56) |
| Target gene: IL6R (IL-6 inhibitors) | CAD | Inverse variance weighted (fixed effects) | 1.38(1.26-1.52) |
| Target gene: IL6R (IL-6 inhibitors) | CAD | Simple median | 1.35(1.10-1.66) |
| Target gene: IL6R (IL-6 inhibitors) | MI | MR Egger | 1.74(1.25-2.43) |
| Target gene: IL6R (IL-6 inhibitors) | MI | Weighted median | 1.35(1.13-1.62) |
| Target gene: IL6R (IL-6 inhibitors) | MI | Inverse variance weighted (multiplicative random effects) | 1.27(1.11-1.44) |
| Target gene: IL6R (IL-6 inhibitors) | MI | Inverse variance weighted (fixed effects) | 1.27(1.14-1.41) |
| Target gene: IL6R (IL-6 inhibitors) | MI | Simple median | 1.33(1.08-1.63) |
| Target gene: IL6R (IL-6 inhibitors) | HF | MR Egger | 1.03(0.77-1.39) |
| Target gene: IL6R (IL-6 inhibitors) | HF | Weighted median | 1.11(0.97-1.28) |
| Target gene: IL6R (IL-6 inhibitors) | HF | Inverse variance weighted (multiplicative random effects) | 1.14(1.02-1.27) |
| Target gene: IL6R (IL-6 inhibitors) | HF | Inverse variance weighted (fixed effects) | 1.14(1.05-1.23) |
| Target gene: IL6R (IL-6 inhibitors) | HF | Simple median | 1.22(1.05-1.42) |
| Target gene: IL6R (IL-6 inhibitors) | IS | MR Egger | 1.30(1.00-1.69) |
| Target gene: IL6R (IL-6 inhibitors) | IS | Weighted median | 1.29(1.12-1.49) |
| Target gene: IL6R (IL-6 inhibitors) | IS | Inverse variance weighted (multiplicative random effects) | 1.34(1.23-1.46) |
| Target gene: IL6R (IL-6 inhibitors) | IS | Inverse variance weighted (fixed effects) | 1.34(1.22-1.48) |
| Target gene: IL6R (IL-6 inhibitors) | IS | Simple median | 1.36(1.15-1.60) |
| Target gene: IL6R (IL-6 inhibitors) | HTN | MR Egger | 0.93(0.71-1.22) |
| Target gene: IL6R (IL-6 inhibitors) | HTN | Weighted median | 0.99(0.87-1.13) |
| Target gene: IL6R (IL-6 inhibitors) | HTN | Inverse variance weighted (multiplicative random effects) | 0.96(0.87-1.06) |
| Target gene: IL6R (IL-6 inhibitors) | HTN | Inverse variance weighted (fixed effects) | 0.96(0.88-1.05) |
| Target gene: IL6R (IL-6 inhibitors) | HTN | Simple median | 0.91(0.78-1.06) |
| Target gene: IL6R (IL-6 inhibitors) | TC | MR Egger | 0.94(0.81-1.09) |
| Target gene: IL6R (IL-6 inhibitors) | TC | Weighted median | 0.98(0.91-1.06) |
| Target gene: IL6R (IL-6 inhibitors) | TC | Inverse variance weighted (multiplicative random effects) | 0.97(0.93-1.01) |
| Target gene: IL6R (IL-6 inhibitors) | TC | Inverse variance weighted (fixed effects) | 0.97(0.91-1.03) |
| Target gene: IL6R (IL-6 inhibitors) | TC | Simple median | 0.95(0.87-1.03) |
| Target gene: IL6R (IL-6 inhibitors) | HDL-C | MR Egger | 1.06(0.92-1.23) |
| Target gene: IL6R (IL-6 inhibitors) | HDL-C | Weighted median | 0.90(0.83-0.98) |
| Target gene: IL6R (IL-6 inhibitors) | HDL-C | Inverse variance weighted (multiplicative random effects) | 0.93(0.88-0.99) |
| Target gene: IL6R (IL-6 inhibitors) | HDL-C | Inverse variance weighted (fixed effects) | 0.93(0.88-0.98) |
| Target gene: IL6R (IL-6 inhibitors) | HDL-C | Simple median | 0.88(0.81-0.97) |
| Target gene: IL6R (IL-6 inhibitors) | LDL-C | MR Egger | 0.91(0.78-1.06) |
| Target gene: IL6R (IL-6 inhibitors) | LDL-C | Weighted median | 1.01(0.93-1.09) |
| Target gene: IL6R (IL-6 inhibitors) | LDL-C | Inverse variance weighted (multiplicative random effects) | 1.00(0.95-1.05) |
| Target gene: IL6R (IL-6 inhibitors) | LDL-C | Inverse variance weighted (fixed effects) | 1.00(0.94-1.06) |
| Target gene: IL6R (IL-6 inhibitors) | LDL-C | Simple median | 1.02(0.94-1.12) |
| Target gene: IL6R (IL-6 inhibitors) | TG | MR Egger | 1.00(0.86-1.16) |
| Target gene: IL6R (IL-6 inhibitors) | TG | Weighted median | 0.97(0.90-1.04) |
| Target gene: IL6R (IL-6 inhibitors) | TG | Inverse variance weighted (multiplicative random effects) | 0.96(0.90-1.01) |
| Target gene: IL6R (IL-6 inhibitors) | TG | Inverse variance weighted (fixed effects) | 0.96(0.91-1.01) |
| Target gene: IL6R (IL-6 inhibitors) | TG | Simple median | 0.98(0.90-1.07) |

| | | | |
|---|---|---|---|
| Target gene: IL6R (IL-6 inhibitors) | AS | MR Egger | 1.57(0.98-2.51) |
| Target gene: IL6R (IL-6 inhibitors) | AS | Weighted median | 1.56(1.21-2.03) |
| Target gene: IL6R (IL-6 inhibitors) | AS | Inverse variance weighted (multiplicative random effects) | 1.75(1.46-2.09) |
| Target gene: IL6R (IL-6 inhibitors) | AS | Inverse variance weighted (fixed effects) | 1.75(1.52-2.01) |
| Target gene: IL6R (IL-6 inhibitors) | AS | Simple median | 1.93(1.46-2.56) |

Abbreviations: SE, standard error; AF, atrial fibrillation; CAD, coronary artery disease; MI, myocardial infarction; HF, heart failure; IS, ischemic stroke; HTN, hypertension; TC, total cholesterol; HDL-C, high density lipoprotein cholesterol; LDL-C, low-density lipoprotein cholesterol; TG, triglycerides; AS, aortic stenosis.

**Table S10.** Heterogeneity and MR-Egger pleiotropy test results in MR analysis.

| Drug Target | Outcome | Cochran's Q | Q_pval | $I^2$ | Egger_intercept | SE | P |
|---|---|---|---|---|---|---|---|
| Target gene: CD80 (Abatacept) | AS | 5.332 | 0.070 | 62.49% | 0.095 | 0.041 | 0.260 |
| Target gene: CD80 (Abatacept) | AF | 0.968 | 0.616 | 0% | -0.019 | 0.019 | 0.505 |
| Target gene: CD80 (Abatacept) | IS | 1.454 | 0.483 | 0% | -0.033 | 0.029 | 0.460 |
| Target gene: CD80 (Abatacept) | HF | 2.557 | 0.278 | 21.79% | -0.036 | 0.023 | 0.358 |
| Target gene: CD80 (Abatacept) | HTN | 1.731 | 0.421 | 0% | -0.034 | 0.026 | 0.415 |
| Target gene: CD80 (Abatacept) | HDL-C | 2.297 | 0.130 | 56.46% | NA | NA | NA |
| Target gene: CD80 (Abatacept) | LDL-C | 0.023 | 0.879 | 0% | NA | NA | NA |
| Target gene: CD80 (Abatacept) | TC | 0.067 | 0.796 | 0% | NA | NA | NA |
| Target gene: CD80 (Abatacept) | TG | 0.987 | 0.321 | 0% | NA | NA | NA |
| Target gene: CD80 (Abatacept) | CAD | 0.399 | 0.819 | 0% | -0.016 | 0.026 | 0.644 |
| Target gene: CD80 (Abatacept) | MI | 0.408 | 0.816 | 0% | -0.018 | 0.029 | 0.639 |
| Target gene: CD80 (Abatacept) | MVP | 0.029 | 0.865 | 0% | NA | NA | NA |
| Target gene: IL6R (IL-6 inhibitors) | AS | 84.227 | 0.004 | 37.07% | 0.005 | 0.011 | 0.633 |
| Target gene: IL6R (IL-6 inhibitors) | AF | 149.394 | 0.000 | 61.85% | -0.004 | 0.006 | 0.577 |
| Target gene: IL6R (IL-6 inhibitors) | IS | 44.689 | 0.882 | 0% | 0.002 | 0.006 | 0.803 |
| Target gene: IL6R (IL-6 inhibitors) | HF | 100.938 | 0.000 | 49.47% | 0.005 | 0.007 | 0.506 |
| Target gene: IL6R (IL-6 inhibitors) | HTN | 75.420 | 0.043 | 25.75% | 0.002 | 0.006 | 0.807 |
| Target gene: IL6R (IL-6 inhibitors) | HDL-C | 25.461 | 0.228 | 17.52% | -0.007 | 0.004 | 0.067 |
| Target gene: IL6R (IL-6 inhibitors) | LDL-C | 16.177 | 0.760 | 0% | 0.005 | 0.004 | 0.208 |
| Target gene: IL6R (IL-6 inhibitors) | TC | 10.609 | 0.970 | 0% | 0.002 | 0.004 | 0.682 |
| Target gene: IL6R (IL-6 inhibitors) | TG | 24.194 | 0.284 | 13.20% | -0.002 | 0.004 | 0.543 |
| Target gene: IL6R (IL-6 inhibitors) | CAD | 92.588 | 0.002 | 39.52% | -0.010 | 0.007 | 0.206 |
| Target gene: IL6R (IL-6 inhibitors) | MI | 85.201 | 0.007 | 34.27% | -0.016 | 0.008 | 0.048 |
| Target gene: IL6R (IL-6 inhibitors) | MVP | 50.422 | 0.340 | 6.79% | -0.021 | 0.017 | 0.219 |
| Target gene: JAK1 (JAK inhibitors) | AS | 23.204 | 0.872 | 0% | -0.001 | 0.007 | 0.865 |
| Target gene: JAK1 (JAK inhibitors) | AF | 31.009 | 0.705 | 0% | 0.003 | 0.003 | 0.369 |
| Target gene: JAK1 (JAK inhibitors) | IS | 35.561 | 0.442 | 1.58% | -0.001 | 0.005 | 0.792 |
| Target gene: JAK1 (JAK inhibitors) | HF | 20.682 | 0.898 | 0% | -0.003 | 0.004 | 0.392 |
| Target gene: JAK1 (JAK inhibitors) | HTN | 68.222 | 0.000 | 50.16% | -0.005 | 0.006 | 0.382 |
| Target gene: JAK1 (JAK inhibitors) | HDL-C | 33.294 | 0.031 | 39.93% | 0.004 | 0.003 | 0.265 |
| Target gene: JAK1 (JAK inhibitors) | LDL-C | 20.979 | 0.398 | 4.67% | -0.001 | 0.003 | 0.643 |
| Target gene: JAK1 (JAK inhibitors) | TC | 28.602 | 0.096 | 30.07% | -0.002 | 0.003 | 0.441 |
| Target gene: JAK1 (JAK inhibitors) | TG | 16.168 | 0.706 | 0% | 0.001 | 0.002 | 0.600 |
| Target gene: JAK1 (JAK inhibitors) | CAD | 25.715 | 0.813 | 0% | 0.004 | 0.004 | 0.364 |
| Target gene: JAK1 (JAK inhibitors) | MI | 24.968 | 0.841 | 0% | 0.008 | 0.005 | 0.089 |
| Target gene: JAK1 (JAK inhibitors) | MVP | 27.640 | 0.430 | 2.32% | -0.005 | 0.012 | 0.658 |
| Target gene: JAK3 (JAK inhibitors) | AS | 0.223 | 0.636 | 0% | NA | NA | NA |
| Target gene: JAK3 (JAK inhibitors) | AF | 0.878 | 0.349 | 0% | NA | NA | NA |
| Target gene: JAK3 (JAK inhibitors) | IS | 0.018 | 0.893 | 0% | NA | NA | NA |

| | | | | | | | |
|---|---|---|---|---|---|---|---|
| Target gene: JAK3 (JAK inhibitors) | HF | 0.320 | 0.572 | 0% | NA | NA | NA |
| Target gene: JAK3 (JAK inhibitors) | HTN | 2.307 | 0.129 | 56.66% | NA | NA | NA |
| Target gene: JAK3 (JAK inhibitors) | HDL-C | 0.995 | 0.318 | 0% | NA | NA | NA |
| Target gene: JAK3 (JAK inhibitors) | LDL-C | 0.905 | 0.341 | 0% | NA | NA | NA |
| Target gene: JAK3 (JAK inhibitors) | TC | 0.082 | 0.775 | 0% | NA | NA | NA |
| Target gene: JAK3 (JAK inhibitors) | TG | 0.019 | 0.891 | 0% | NA | NA | NA |
| Target gene: JAK3 (JAK inhibitors) | CAD | 0.000 | 0.993 | 0% | NA | NA | NA |
| Target gene: JAK3 (JAK inhibitors) | MI | 0.399 | 0.528 | 0% | NA | NA | NA |
| Target gene: JAK3 (JAK inhibitors) | MVP | 0.000 | 0.987 | 0% | NA | NA | NA |
| Target gene: PLA2G1B (Sulfasalazine) | AS | 67.415 | 0.267 | 9.52% | 0.001 | 0.007 | 0.868 |
| Target gene: PLA2G1B (Sulfasalazine) | AF | 140.281 | 0.000 | 52.24% | 0.000 | 0.004 | 0.987 |
| Target gene: PLA2G1B (Sulfasalazine) | IS | 64.101 | 0.508 | 0% | 0.005 | 0.004 | 0.221 |
| Target gene: PLA2G1B (Sulfasalazine) | HF | 56.469 | 0.532 | 0% | -0.005 | 0.004 | 0.203 |
| Target gene: PLA2G1B (Sulfasalazine) | HTN | 96.694 | 0.008 | 31.74% | 0.003 | 0.005 | 0.483 |
| Target gene: PLA2G1B (Sulfasalazine) | HDL-C | 65.232 | 0.001 | 46.35% | -0.003 | 0.003 | 0.379 |
| Target gene: PLA2G1B (Sulfasalazine) | LDL-C | 81.001 | 0.000 | 56.79% | 0.000 | 0.004 | 0.933 |
| Target gene: PLA2G1B (Sulfasalazine) | TC | 58.554 | 0.008 | 40.23% | -0.002 | 0.003 | 0.430 |
| Target gene: PLA2G1B (Sulfasalazine) | TG | 31.417 | 0.642 | 0% | 0.001 | 0.002 | 0.558 |
| Target gene: PLA2G1B (Sulfasalazine) | CAD | 88.741 | 0.022 | 27.88% | 0.004 | 0.005 | 0.433 |
| Target gene: PLA2G1B (Sulfasalazine) | MI | 85.592 | 0.031 | 26.40% | 0.004 | 0.005 | 0.418 |
| Target gene: PLA2G1B (Sulfasalazine) | MVP | 48.893 | 0.216 | 14.10% | 0.022 | 0.014 | 0.126 |
| Target gene: PPIF (Cyclosporine) | AS | 2.557 | 0.634 | 0% | -0.012 | 0.033 | 0.747 |
| Target gene: PPIF (Cyclosporine) | AF | 22.855 | 0.000 | 82.50% | -0.048 | 0.030 | 0.205 |
| Target gene: PPIF (Cyclosporine) | IS | 0.586 | 0.965 | 0% | -0.003 | 0.023 | 0.916 |
| Target gene: PPIF (Cyclosporine) | HF | 1.779 | 0.776 | 0% | -0.008 | 0.018 | 0.676 |
| Target gene: PPIF (Cyclosporine) | HTN | 1.220 | 0.875 | 0% | 0.010 | 0.020 | 0.657 |
| Target gene: PPIF (Cyclosporine) | HDL-C | 2.194 | 0.533 | 0% | -0.013 | 0.014 | 0.438 |
| Target gene: PPIF (Cyclosporine) | LDL-C | 3.791 | 0.285 | 20.87% | -0.003 | 0.021 | 0.894 |
| Target gene: PPIF (Cyclosporine) | TC | 1.328 | 0.515 | 0% | -0.004 | 0.016 | 0.843 |
| Target gene: PPIF (Cyclosporine) | TG | 1.747 | 0.627 | 0% | -0.007 | 0.013 | 0.648 |
| Target gene: PPIF (Cyclosporine) | CAD | 3.589 | 0.464 | 0% | -0.001 | 0.023 | 0.962 |
| Target gene: PPIF (Cyclosporine) | MI | 5.363 | 0.252 | 25.42% | -0.010 | 0.031 | 0.758 |
| Target gene: PPIF (Cyclosporine) | MVP | 7.287 | 0.063 | 58.83% | -0.073 | 0.090 | 0.504 |
| Target gene: RELA (Sulfasalazine) | AS | 0.428 | 0.980 | 0% | -0.002 | 0.046 | 0.973 |
| Target gene: RELA (Sulfasalazine) | AF | 0.618 | 0.961 | 0% | -0.011 | 0.022 | 0.639 |
| Target gene: RELA (Sulfasalazine) | IS | 5.361 | 0.252 | 25.39% | -0.014 | 0.044 | 0.770 |
| Target gene: RELA (Sulfasalazine) | HF | 3.441 | 0.487 | 0% | -0.001 | 0.028 | 0.965 |
| Target gene: RELA (Sulfasalazine) | HTN | 4.583 | 0.333 | 12.73% | 0.046 | 0.030 | 0.217 |
| Target gene: RELA (Sulfasalazine) | HDL-C | 9.743 | 0.045 | 58.95% | -0.008 | 0.029 | 0.793 |
| Target gene: RELA (Sulfasalazine) | LDL-C | 8.043 | 0.090 | 50.27% | -0.043 | 0.018 | 0.091 |
| Target gene: RELA (Sulfasalazine) | TC | 10.366 | 0.035 | 61.41% | -0.048 | 0.017 | 0.068 |
| Target gene: RELA (Sulfasalazine) | TG | 10.208 | 0.037 | 60.82% | -0.037 | 0.020 | 0.169 |
| Target gene: RELA (Sulfasalazine) | CAD | 8.844 | 0.065 | 54.77% | -0.007 | 0.052 | 0.900 |
| Target gene: RELA (Sulfasalazine) | MI | 4.576 | 0.334 | 12.59% | -0.008 | 0.041 | 0.866 |

| | | | | | | | |
|---|---|---|---|---|---|---|---|
| Target gene: RELA (Sulfasalazine) | MVP | 1.161 | 0.762 | 0% | 0.008 | 0.098 | 0.945 |
| Target gene: RELB (Sulfasalazine) | AS | 73.228 | 0.438 | 1.68% | 0.005 | 0.004 | 0.219 |
| Target gene: RELB (Sulfasalazine) | AF | 79.522 | 0.181 | 13.23% | -0.004 | 0.002 | 0.042 |
| Target gene: RELB (Sulfasalazine) | IS | 68.197 | 0.369 | 4.69% | -0.005 | 0.003 | 0.080 |
| Target gene: RELB (Sulfasalazine) | HF | 73.823 | 0.354 | 5.18% | 0.004 | 0.002 | 0.063 |
| Target gene: RELB (Sulfasalazine) | HTN | 132.435 | 0.000 | 42.61% | -0.004 | 0.003 | 0.204 |
| Target gene: RELB (Sulfasalazine) | HDL-C | 123.689 | 0.000 | 75.75% | -0.004 | 0.003 | 0.197 |
| Target gene: RELB (Sulfasalazine) | LDL-C | 2864.863 | 0.000 | 98.95% | 0.003 | 0.017 | 0.868 |
| Target gene: RELB (Sulfasalazine) | TC | 1209.482 | 0.000 | 97.52% | 0.001 | 0.011 | 0.926 |
| Target gene: RELB (Sulfasalazine) | TG | 352.796 | 0.000 | 91.50% | 0.003 | 0.005 | 0.584 |
| Target gene: RELB (Sulfasalazine) | CAD | 168.152 | 0.000 | 56.59% | 0.005 | 0.004 | 0.167 |
| Target gene: RELB (Sulfasalazine) | MI | 136.424 | 0.000 | 47.22% | 0.008 | 0.004 | 0.032 |
| Target gene: RELB (Sulfasalazine) | MVP | 89.319 | 0.005 | 35.06% | -0.007 | 0.009 | 0.447 |
| Target gene: TNF/LTA (TNF-alpha inhibitors) | AS | 6.912 | 0.546 | 0% | -0.014 | 0.018 | 0.473 |
| Target gene: TNF/LTA (TNF-alpha inhibitors) | AF | 17.116 | 0.047 | 47.42% | 0.003 | 0.014 | 0.807 |
| Target gene: TNF/LTA (TNF-alpha inhibitors) | IS | 2.722 | 0.974 | 0% | -0.004 | 0.016 | 0.827 |
| Target gene: TNF/LTA (TNF-alpha inhibitors) | HF | 11.386 | 0.250 | 20.95% | -0.004 | 0.012 | 0.772 |
| Target gene: TNF/LTA (TNF-alpha inhibitors) | HTN | 10.084 | 0.121 | 40.50% | -0.013 | 0.015 | 0.439 |
| Target gene: TNF/LTA (TNF-alpha inhibitors) | HDL-C | 29.522 | 0.000 | 86.45% | -0.016 | 0.018 | 0.443 |
| Target gene: TNF/LTA (TNF-alpha inhibitors) | LDL-C | 41.737 | 0.000 | 90.42% | -0.006 | 0.026 | 0.842 |
| Target gene: TNF/LTA (TNF-alpha inhibitors) | TC | 65.270 | 0.000 | 93.87% | -0.018 | 0.030 | 0.580 |
| Target gene: TNF/LTA (TNF-alpha inhibitors) | TG | 14.570 | 0.006 | 72.55% | -0.006 | 0.013 | 0.695 |
| Target gene: TNF/LTA (TNF-alpha inhibitors) | CAD | 20.052 | 0.001 | 75.07% | 0.010 | 0.037 | 0.808 |
| Target gene: TNF/LTA (TNF-alpha inhibitors) | MI | 8.472 | 0.132 | 40.98% | 0.004 | 0.027 | 0.901 |
| Target gene: TNF/LTA (TNF-alpha inhibitors) | MVP | 1.821 | 0.873 | 0% | -0.041 | 0.039 | 0.358 |
| Abatacept (CD80) | AS | 5.332 | 0.070 | 62.49% | 0.095 | 0.041 | 0.260 |
| Abatacept (CD80) | AF | 0.968 | 0.616 | 0% | -0.019 | 0.019 | 0.505 |
| Abatacept (CD80) | IS | 1.454 | 0.483 | 0% | -0.033 | 0.029 | 0.460 |
| Abatacept (CD80) | HF | 2.557 | 0.278 | 21.79% | -0.036 | 0.023 | 0.358 |
| Abatacept (CD80) | HTN | 1.731 | 0.421 | 0% | -0.034 | 0.026 | 0.415 |
| Abatacept (CD80) | HDL-C | 2.297 | 0.130 | 56.46% | NA | NA | NA |
| Abatacept (CD80) | LDL-C | 0.023 | 0.879 | 0% | NA | NA | NA |
| Abatacept (CD80) | TC | 0.067 | 0.796 | 0% | NA | NA | NA |
| Abatacept (CD80) | TG | 0.987 | 0.321 | 0% | NA | NA | NA |
| Abatacept (CD80) | CAD | 0.399 | 0.819 | 0% | -0.016 | 0.026 | 0.644 |
| Abatacept (CD80) | MI | 0.408 | 0.816 | 0% | -0.018 | 0.029 | 0.639 |
| Abatacept (CD80) | MVP | 0.029 | 0.865 | 0% | NA | NA | NA |
| TNF-alpha inhibitors (TNF/LTA) | AS | 6.912 | 0.546 | 0% | -0.014 | 0.018 | 0.473 |
| TNF-alpha inhibitors (TNF/LTA) | AF | 17.116 | 0.047 | 47.42% | 0.003 | 0.014 | 0.807 |
| TNF-alpha inhibitors (TNF/LTA) | IS | 2.722 | 0.974 | 0% | -0.004 | 0.016 | 0.827 |
| TNF-alpha inhibitors (TNF/LTA) | HF | 11.386 | 0.250 | 20.95% | -0.004 | 0.012 | 0.772 |
| TNF-alpha inhibitors (TNF/LTA) | HTN | 10.084 | 0.121 | 40.50% | -0.013 | 0.015 | 0.439 |
| TNF-alpha inhibitors (TNF/LTA) | HDL-C | 29.522 | 0.000 | 86.45% | -0.016 | 0.018 | 0.443 |
| TNF-alpha inhibitors (TNF/LTA) | LDL-C | 41.737 | 0.000 | 90.42% | -0.006 | 0.026 | 0.842 |

| | | | | | | | |
|---|---|---|---|---|---|---|---|
| TNF-alpha inhibitors (TNF/LTA) | TC | 65.270 | 0.000 | 93.87% | -0.018 | 0.030 | 0.580 |
| TNF-alpha inhibitors (TNF/LTA) | TG | 14.570 | 0.006 | 72.55% | -0.006 | 0.013 | 0.695 |
| TNF-alpha inhibitors (TNF/LTA) | CAD | 20.052 | 0.001 | 75.07% | 0.010 | 0.037 | 0.808 |
| TNF-alpha inhibitors (TNF/LTA) | MI | 8.472 | 0.132 | 40.98% | 0.004 | 0.027 | 0.901 |
| TNF-alpha inhibitors (TNF/LTA) | MVP | 1.821 | 0.873 | 0% | -0.041 | 0.039 | 0.358 |
| Cyclosporine (PPIF) | AS | 2.557 | 0.634 | 0% | -0.012 | 0.033 | 0.747 |
| Cyclosporine (PPIF) | AF | 22.855 | 0.000 | 82.50% | -0.048 | 0.030 | 0.205 |
| Cyclosporine (PPIF) | IS | 0.586 | 0.965 | 0% | -0.003 | 0.023 | 0.916 |
| Cyclosporine (PPIF) | HF | 1.779 | 0.776 | 0% | -0.008 | 0.018 | 0.676 |
| Cyclosporine (PPIF) | HTN | 1.220 | 0.875 | 0% | 0.010 | 0.020 | 0.657 |
| Cyclosporine (PPIF) | HDL-C | 3.659 | 0.454 | 0% | -0.002 | 0.013 | 0.887 |
| Cyclosporine (PPIF) | LDL-C | 7.691 | 0.104 | 47.99% | -0.016 | 0.018 | 0.456 |
| Cyclosporine (PPIF) | TC | 2.027 | 0.731 | 0% | -0.003 | 0.013 | 0.819 |
| Cyclosporine (PPIF) | TG | 2.184 | 0.702 | 0% | -0.001 | 0.011 | 0.927 |
| Cyclosporine (PPIF) | CAD | 3.589 | 0.464 | 0% | -0.001 | 0.023 | 0.962 |
| Cyclosporine (PPIF) | MI | 5.363 | 0.252 | 25.42% | -0.010 | 0.031 | 0.758 |
| Cyclosporine (PPIF) | MVP | 7.287 | 0.063 | 58.83% | -0.073 | 0.090 | 0.504 |
| Sulfasalazine (PLA2G1B+RELB+RELA) | AS | 154.877 | 0.169 | 10.25% | 0.003 | 0.003 | 0.395 |
| Sulfasalazine (PLA2G1B+RELB+RELA) | AF | 227.698 | 0.000 | 37.64% | -0.001 | 0.002 | 0.611 |
| Sulfasalazine (PLA2G1B+RELB+RELA) | IS | 141.429 | 0.357 | 3.84% | -0.005 | 0.002 | 0.017 |
| Sulfasalazine (PLA2G1B+RELB+RELA) | HF | 141.897 | 0.304 | 5.57% | 0.005 | 0.002 | 0.002 |
| Sulfasalazine (PLA2G1B+RELB+RELA) | HTN | 244.737 | 0.000 | 39.53% | -0.001 | 0.002 | 0.730 |
| Sulfasalazine (PLA2G1B+RELB+RELA) | HDL-C | 244.501 | 0.000 | 70.96% | -0.003 | 0.002 | 0.179 |
| Sulfasalazine (PLA2G1B+RELB+RELA) | LDL-C | 4957.221 | 0.000 | 98.57% | 0.006 | 0.011 | 0.566 |
| Sulfasalazine (PLA2G1B+RELB+RELA) | TC | 2634.328 | 0.000 | 97.30% | 0.005 | 0.008 | 0.489 |
| Sulfasalazine (PLA2G1B+RELB+RELA) | TG | 441.607 | 0.000 | 83.92% | 0.003 | 0.003 | 0.305 |
| Sulfasalazine (PLA2G1B+RELB+RELA) | CAD | 341.194 | 0.000 | 58.09% | 0.007 | 0.003 | 0.016 |
| Sulfasalazine (PLA2G1B+RELB+RELA) | MI | 284.675 | 0.000 | 50.47% | 0.009 | 0.003 | 0.003 |
| Sulfasalazine (PLA2G1B+RELB+RELA) | MVP | | | | | | |
| IL-6 inhibitors (IL6R) | AS | 84.227 | 0.004 | 37.07% | 0.005 | 0.011 | 0.633 |
| IL-6 inhibitors (IL6R) | AF | 149.394 | 0.000 | 61.85% | -0.004 | 0.006 | 0.577 |
| IL-6 inhibitors (IL6R) | IS | 44.689 | 0.882 | 0% | 0.002 | 0.006 | 0.803 |
| IL-6 inhibitors (IL6R) | HF | 100.938 | 0.000 | 49.47% | 0.005 | 0.007 | 0.506 |
| IL-6 inhibitors (IL6R) | HTN | 75.420 | 0.043 | 25.75% | 0.002 | 0.006 | 0.807 |
| IL-6 inhibitors (IL6R) | HDL-C | 25.461 | 0.228 | 17.52% | -0.007 | 0.004 | 0.067 |
| IL-6 inhibitors (IL6R) | LDL-C | 16.177 | 0.760 | 0% | 0.005 | 0.004 | 0.208 |
| IL-6 inhibitors (IL6R) | TC | 10.609 | 0.970 | 0% | 0.002 | 0.004 | 0.682 |
| IL-6 inhibitors (IL6R) | TG | 24.194 | 0.284 | 13.20% | -0.002 | 0.004 | 0.543 |
| IL-6 inhibitors (IL6R) | CAD | 92.588 | 0.002 | 39.52% | -0.010 | 0.007 | 0.206 |
| IL-6 inhibitors (IL6R) | MI | 85.201 | 0.007 | 34.27% | -0.016 | 0.008 | 0.048 |
| IL-6 inhibitors (IL6R) | MVP | 50.422 | 0.340 | 6.79% | -0.021 | 0.017 | 0.219 |
| JAK inhibitors (JAK1+JAK3+TYK2) | AS | 25.416 | 0.883 | 0% | 0.000 | 0.006 | 0.953 |
| JAK inhibitors (JAK1+JAK3+TYK2) | AF | 34.471 | 0.676 | 0% | 0.004 | 0.003 | 0.193 |
| JAK inhibitors (JAK1+JAK3+TYK2) | IS | 36.428 | 0.542 | 0% | -0.002 | 0.004 | 0.704 |

| | | | | | | | |
|---|---|---|---|---|---|---|---|
| JAK inhibitors (JAK1+JAK3+TYK2) | HF | 23.058 | 0.901 | 0% | -0.002 | 0.003 | 0.543 |
| JAK inhibitors (JAK1+JAK3+TYK2) | HTN | 80.364 | 0.000 | 53.96% | 0.000 | 0.005 | 0.927 |
| JAK inhibitors (JAK1+JAK3+TYK2) | HDL-C | 35.373 | 0.048 | 34.98% | 0.002 | 0.003 | 0.435 |
| JAK inhibitors (JAK1+JAK3+TYK2) | LDL-C | 37.154 | 0.031 | 38.09% | -0.001 | 0.003 | 0.678 |
| JAK inhibitors (JAK1+JAK3+TYK2) | TC | 44.675 | 0.004 | 48.52% | -0.003 | 0.003 | 0.368 |
| JAK inhibitors (JAK1+JAK3+TYK2) | TG | 27.001 | 0.256 | 14.82% | 0.003 | 0.002 | 0.218 |
| JAK inhibitors (JAK1+JAK3+TYK2) | CAD | 35.687 | 0.483 | 0% | 0.008 | 0.004 | 0.042 |
| JAK inhibitors (JAK1+JAK3+TYK2) | MI | 32.780 | 0.623 | 0% | 0.011 | 0.004 | 0.012 |
| JAK inhibitors (JAK1+JAK3+TYK2) | MVP | 27.948 | 0.521 | 0% | -0.007 | 0.011 | 0.528 |

Abbreviations: AF, atrial fibrillation; CAD, coronary artery disease; MI, myocardial infarction; HF, heart failure; IS, ischemic stroke; HTN, hypertension; TC, total cholesterol; HDL-C, high density lipoprotein cholesterol; LDL-C, low-density lipoprotein cholesterol; TG, triglycerides; AS, aortic stenosis.

**Table S11.** Results from the MR-PRESSO analysis.

| Drug Target | Outcome | Outlier.corrected.Beta | Outlier.corrected.Beta.Sd | Outlier.corrected.Beta.P | Global.Test.Pvalue | Global.RSSobs | Distortion.Test |
|---|---|---|---|---|---|---|---|
| Target gene: IL6R (IL-6 inhibitors) | AS | 0.610 | 0.087 | 0.000 | 0.007 | 90.041 | 0.667 |
| Target gene: IL6R (IL-6 inhibitors) | AF | 0.208 | 0.053 | 0.000 | <0.001 | 157.372 | 0.182 |
| Target gene: IL6R (IL-6 inhibitors) | IS | | | | 0.886 | 46.102 | |
| Target gene: IL6R (IL-6 inhibitors) | HF | | | | <0.001 | 105.611 | |
| Target gene: IL6R (IL-6 inhibitors) | HTN | | | | 0.039 | 77.606 | |
| Target gene: IL6R (IL-6 inhibitors) | HDL-C | | | | 0.207 | 28.621 | |
| Target gene: IL6R (IL-6 inhibitors) | LDL-C | | | | 0.819 | 16.938 | |
| Target gene: IL6R (IL-6 inhibitors) | TC | | | | 0.973 | 11.264 | |
| Target gene: IL6R (IL-6 inhibitors) | TG | | | | 0.319 | 25.839 | |
| Target gene: IL6R (IL-6 inhibitors) | CAD | 0.246 | 0.057 | 0.000 | 0.003 | 99.333 | 0.079 |
| Target gene: IL6R (IL-6 inhibitors) | MI | 0.162 | 0.064 | 0.014 | 0.004 | 91.325 | 0.097 |
| Target gene: IL6R (IL-6 inhibitors) | MVP | | | | 0.343 | 52.840 | |
| Target gene: JAK1 (JAK inhibitors) | AS | | | | 0.892 | 23.963 | |
| Target gene: JAK1 (JAK inhibitors) | AF | | | | 0.728 | 32.264 | |
| Target gene: JAK1 (JAK inhibitors) | IS | | | | 0.419 | 38.682 | |
| Target gene: JAK1 (JAK inhibitors) | HF | | | | 0.897 | 21.950 | |
| Target gene: JAK1 (JAK inhibitors) | HTN | | | | 0.002 | 70.611 | |
| Target gene: JAK1 (JAK inhibitors) | HDL-C | -0.050 | 0.043 | 0.259 | 0.044 | 36.651 | 0.813 |
| Target gene: JAK1 (JAK inhibitors) | LDL-C | | | | 0.433 | 23.067 | |
| Target gene: JAK1 (JAK inhibitors) | TC | | | | 0.130 | 32.175 | |
| Target gene: JAK1 (JAK inhibitors) | TG | | | | 0.690 | 18.065 | |
| Target gene: JAK1 (JAK inhibitors) | CAD | | | | 0.810 | 27.711 | |
| Target gene: JAK1 (JAK inhibitors) | MI | | | | 0.802 | 27.771 | |
| Target gene: JAK1 (JAK inhibitors) | MVP | | | | 0.502 | 28.642 | |
| Target gene: TNF/LTA (TNF-alpha inhibitors) | AS | | | | 0.589 | 8.407 | |
| Target gene: TNF/LTA (TNF-alpha inhibitors) | AF | | | | 0.063 | 21.027 | |
| Target gene: TNF/LTA (TNF-alpha inhibitors) | IS | | | | 0.979 | 3.283 | |
| Target gene: TNF/LTA (TNF-alpha inhibitors) | HF | | | | 0.249 | 14.541 | |
| Target gene: TNF/LTA (TNF-alpha inhibitors) | HTN | | | | 0.215 | 12.930 | |
| Target gene: TNF/LTA (TNF-alpha inhibitors) | HDL-C | -0.143 | 0.224 | 0.639 | 0.003 | 43.797 | 1.000 |
| Target gene: TNF/LTA (TNF-alpha inhibitors) | LDL-C | 0.124 | 0.157 | 0.511 | <0.001 | 60.989 | 0.680 |
| Target gene: TNF/LTA (TNF-alpha inhibitors) | TC | 0.174 | 0.041 | 0.050 | <0.001 | 97.915 | <0.001 |
| Target gene: TNF/LTA (TNF-alpha inhibitors) | TG | -0.116 | 0.059 | 0.187 | 0.025 | 27.216 | 1.000 |
| Target gene: TNF/LTA (TNF-alpha inhibitors) | CAD | 0.672 | 0.375 | 0.171 | 0.004 | 25.957 | 1.000 |
| Target gene: TNF/LTA (TNF-alpha inhibitors) | MI | | | | 0.194 | 10.963 | |
| Target gene: TNF/LTA (TNF-alpha inhibitors) | MVP | | | | 0.873 | 2.542 | |
| Target gene: PLA2G1B (Sulfasalazine) | AS | | | | 0.278 | 69.377 | |
| Target gene: PLA2G1B (Sulfasalazine) | AF | -0.014 | 0.047 | 0.767 | <0.001 | 146.282 | 0.119 |
| Target gene: PLA2G1B (Sulfasalazine) | IS | | | | 0.527 | 65.965 | |

| | | | | | | | |
|---|---|---|---|---|---|---|---|
| Target gene: PLA2G1B (Sulfasalazine) | HF | | | | 0.561 | 58.168 | |
| Target gene: PLA2G1B (Sulfasalazine) | TC | -0.136 | 0.030 | 0.000 | 0.003 | 63.019 | 0.606 |
| Target gene: PLA2G1B (Sulfasalazine) | HTN | | | | 0.010 | 98.952 | |
| Target gene: PLA2G1B (Sulfasalazine) | CAD | | | | 0.034 | 90.926 | |
| Target gene: PLA2G1B (Sulfasalazine) | MI | | | | 0.028 | 87.585 | |
| Target gene: PLA2G1B (Sulfasalazine) | HDL-C | | | | 0.001 | 68.937 | |
| Target gene: PLA2G1B (Sulfasalazine) | MVP | | | | 0.222 | 50.942 | |
| Target gene: PLA2G1B (Sulfasalazine) | LDL-C | -0.171 | 0.034 | 0.000 | <0.001 | 89.831 | 0.388 |
| Target gene: PLA2G1B (Sulfasalazine) | TG | | | | 0.649 | 32.688 | |
| Target gene: PPIF (Cyclosporine) | AS | | | | 0.685 | 3.667 | |
| Target gene: PPIF (Cyclosporine) | AF | 0.438 | 0.373 | 0.361 | 0.009 | 34.049 | 1.000 |
| Target gene: PPIF (Cyclosporine) | IS | | | | 0.975 | 0.833 | |
| Target gene: PPIF (Cyclosporine) | HF | | | | 0.803 | 2.520 | |
| Target gene: PPIF (Cyclosporine) | HTN | | | | 0.890 | 1.780 | |
| Target gene: PPIF (Cyclosporine) | HDL-C | | | | 0.526 | 4.283 | |
| Target gene: PPIF (Cyclosporine) | HDL-C | | | | 0.373 | 5.946 | |
| Target gene: PPIF (Cyclosporine) | TC | | | | 0.766 | 2.915 | |
| Target gene: PPIF (Cyclosporine) | TG | | | | 0.706 | 2.666 | |
| Target gene: PPIF (Cyclosporine) | CAD | | | | 0.573 | 4.917 | |
| Target gene: PPIF (Cyclosporine) | MI | | | | 0.338 | 7.494 | |
| Target gene: PPIF (Cyclosporine) | MVP | | | | 0.151 | 12.365 | |
| Target gene: RELA (Sulfasalazine) | AS | | | | 0.971 | 0.741 | |
| Target gene: RELA (Sulfasalazine) | AF | | | | 0.957 | 1.036 | |
| Target gene: RELA (Sulfasalazine) | IS | | | | 0.335 | 7.410 | |
| Target gene: RELA (Sulfasalazine) | HF | | | | 0.582 | 4.826 | |
| Target gene: RELA (Sulfasalazine) | HTN | | | | 0.408 | 6.930 | |
| Target gene: RELA (Sulfasalazine) | HDL-C | | | | 0.084 | 14.732 | |
| Target gene: RELA (Sulfasalazine) | LDL-C | | | | 0.131 | 13.208 | |
| Target gene: RELA (Sulfasalazine) | TC | | | | 0.076 | 16.422 | |
| Target gene: RELA (Sulfasalazine) | TG | | | | 0.080 | 17.420 | |
| Target gene: RELA (Sulfasalazine) | CAD | | | | 0.147 | 12.844 | |
| Target gene: RELA (Sulfasalazine) | MI | | | | 0.460 | 6.492 | |
| Target gene: RELA (Sulfasalazine) | MVP | | | | 0.774 | 2.152 | |
| Target gene: RELB (Sulfasalazine) | AS | | | | 0.445 | 75.097 | |
| Target gene: RELB (Sulfasalazine) | AF | | | | 0.147 | 84.977 | |
| Target gene: RELB (Sulfasalazine) | IS | | | | 0.356 | 72.675 | |
| Target gene: RELB (Sulfasalazine) | HF | | | | 0.389 | 74.865 | |
| Target gene: RELB (Sulfasalazine) | HTN | | | | <0.001 | 138.771 | |
| Target gene: RELB (Sulfasalazine) | HDL-C | 0.332 | 0.027 | 0.000 | <0.001 | 130.434 | 0.236 |
| Target gene: RELB (Sulfasalazine) | LDL-C | -1.459 | 0.066 | 0.000 | <0.001 | 3224.903 | 0.006 |
| Target gene: RELB (Sulfasalazine) | TC | -1.144 | 0.066 | 0.000 | <0.001 | 1349.049 | 0.062 |
| Target gene: RELB (Sulfasalazine) | TG | -0.194 | 0.047 | 0.000 | <0.001 | 410.568 | 0.770 |
| Target gene: RELB (Sulfasalazine) | CAD | -0.263 | 0.049 | 0.000 | <0.001 | 173.592 | 0.187 |
| Target gene: RELB (Sulfasalazine) | MI | -0.268 | 0.050 | 0.000 | <0.001 | 139.319 | 0.132 |

| | | | | | | | |
|---|---|---|---|---|---|---|---|
| Target gene: RELB (Sulfasalazine) | MVP | | | | 0.012 | 91.406 | |
| TNF-alpha inhibitors (TNF/LTA) | AS | | | | 0.571 | 8.407 | |
| TNF-alpha inhibitors (TNF/LTA) | AF | 0.132 | 0.139 | 0.369 | 0.047 | 21.027 | |
| TNF-alpha inhibitors (TNF/LTA) | IS | | | | 0.977 | 3.283 | |
| TNF-alpha inhibitors (TNF/LTA) | HF | | | | 0.252 | 14.541 | |
| TNF-alpha inhibitors (TNF/LTA) | HTN | | | | 0.209 | 12.930 | |
| TNF-alpha inhibitors (TNF/LTA) | HDL-C | -0.143 | 0.224 | 0.639 | <0.001 | 43.797 | |
| TNF-alpha inhibitors (TNF/LTA) | LDL-C | 0.124 | 0.157 | 0.511 | <0.001 | 60.989 | |
| TNF-alpha inhibitors (TNF/LTA) | TC | 0.174 | 0.041 | 0.050 | <0.001 | 97.915 | |
| TNF-alpha inhibitors (TNF/LTA) | TG | -0.116 | 0.059 | 0.187 | 0.030 | 27.216 | |
| TNF-alpha inhibitors (TNF/LTA) | CAD | 0.672 | 0.375 | 0.171 | 0.004 | 25.957 | |
| TNF-alpha inhibitors (TNF/LTA) | MI | | | | 0.202 | 10.963 | |
| TNF-alpha inhibitors (TNF/LTA) | MVP | | | | 0.867 | 2.542 | |
| Cyclosporine (PPIF) | AS | | | | 0.668 | 3.667 | |
| Cyclosporine (PPIF) | AF | 0.438 | 0.373 | 0.361 | 0.005 | 34.049 | 1.000 |
| Cyclosporine (PPIF) | IS | | | | 0.977 | 0.833 | |
| Cyclosporine (PPIF) | HF | | | | 0.833 | 2.520 | |
| Cyclosporine (PPIF) | HTN | | | | 0.880 | 1.780 | |
| Cyclosporine (PPIF) | HDL-C | | | | 0.481 | 5.440 | |
| Cyclosporine (PPIF) | LDL-C | | | | 0.175 | 10.936 | |
| Cyclosporine (PPIF) | TC | | | | 0.755 | 2.915 | |
| Cyclosporine (PPIF) | TG | | | | 0.756 | 3.137 | |
| Cyclosporine (PPIF) | CAD | | | | 0.559 | 4.917 | |
| Cyclosporine (PPIF) | MI | | | | 0.347 | 7.494 | |
| Cyclosporine (PPIF) | MVP | | | | 0.159 | 12.365 | |
| Sulfasalazine (PLA2G1B+RELB+RELA) | HDL-C | 0.255 | 0.026 | 0.000 | <0.001 | 255.922 | 0.793 |
| Sulfasalazine (PLA2G1B+RELB+RELA) | LDL-C | -0.602 | 0.069 | 0.000 | <0.001 | 5335.435 | <0.001 |
| Sulfasalazine (PLA2G1B+RELB+RELA) | AS | NA | NA | NA | 0.179 | 157.679 | |
| Sulfasalazine (PLA2G1B+RELB+RELA) | AF | NA | NA | NA | <0.001 | 232.218 | NA |
| Sulfasalazine (PLA2G1B+RELB+RELA) | IS | NA | NA | NA | 0.286 | 146.316 | |
| Sulfasalazine (PLA2G1B+RELB+RELA) | HF | NA | NA | NA | 0.307 | 143.449 | |
| Sulfasalazine (PLA2G1B+RELB+RELA) | HTN | -0.009 | 0.033 | 0.791 | <0.001 | 248.178 | 0.679 |
| Sulfasalazine (PLA2G1B+RELB+RELA) | CAD | -0.106 | 0.040 | 0.009 | <0.001 | 349.354 | 0.528 |
| Sulfasalazine (PLA2G1B+RELB+RELA) | MI | -0.120 | 0.042 | 0.005 | <0.001 | 290.736 | 0.461 |
| Sulfasalazine (PLA2G1B+RELB+RELA) | MVP | | | | | | |
| Sulfasalazine (PLA2G1B+RELB+RELA) | TC | -0.051 | 0.025 | 0.049 | <0.001 | 483.501 | 0.040 |
| Sulfasalazine (PLA2G1B+RELB+RELA) | TG | -0.419 | 0.056 | 0.000 | <0.001 | 2852.603 | <0.001 |
| IL-6 inhibitors (IL6R) | AS | 0.610 | 0.087 | 0.000 | 0.001 | 90.041 | 0.667 |
| IL-6 inhibitors (IL6R) | AF | 0.208 | 0.053 | 0.000 | <0.001 | 157.372 | 0.182 |
| IL-6 inhibitors (IL6R) | IS | | | | 0.887 | 46.102 | |
| IL-6 inhibitors (IL6R) | HF | | | | <0.001 | 105.611 | |
| IL-6 inhibitors (IL6R) | HTN | | | | 0.041 | 77.606 | |
| IL-6 inhibitors (IL6R) | HDL-C | | | | 0.198 | 28.621 | |
| IL-6 inhibitors (IL6R) | LDL-C | | | | 0.802 | 16.938 | |

| | | | | | | | |
|---|---|---|---|---|---|---|---|
| IL-6 inhibitors (IL6R) | TC | | | | 0.970 | 11.264 | |
| IL-6 inhibitors (IL6R) | TG | | | | 0.300 | 25.839 | |
| IL-6 inhibitors (IL6R) | CAD | 0.257 | 0.061 | 0.000 | <0.001 | 99.333 | 0.079 |
| IL-6 inhibitors (IL6R) | MI | 0.162 | 0.064 | 0.014 | 0.009 | 91.325 | 0.097 |
| IL-6 inhibitors (IL6R) | MVP | | | | 0.358 | 52.840 | |
| JAK inhibitors (JAK1+JAK3+TYK2) | AS | | | | 0.896 | 26.187 | |
| JAK inhibitors (JAK1+JAK3+TYK2) | AF | | | | 0.698 | 35.725 | |
| JAK inhibitors (JAK1+JAK3+TYK2) | IS | | | | 0.512 | 39.415 | |
| JAK inhibitors (JAK1+JAK3+TYK2) | HF | | | | 0.906 | 24.262 | |
| JAK inhibitors (JAK1+JAK3+TYK2) | HTN | -0.215 | 0.084 | 0.014 | <0.001 | 82.701 | 0.822 |
| JAK inhibitors (JAK1+JAK3+TYK2) | HDL-C | | | | 0.062 | 38.271 | |
| JAK inhibitors (JAK1+JAK3+TYK2) | LDL-C | | | | 0.062 | 39.649 | |
| JAK inhibitors (JAK1+JAK3+TYK2) | TC | -0.047 | 0.053 | 0.390 | 0.008 | 48.668 | 0.746 |
| JAK inhibitors (JAK1+JAK3+TYK2) | TG | | | | 0.271 | 29.626 | |
| JAK inhibitors (JAK1+JAK3+TYK2) | CAD | | | | 0.471 | 38.438 | |
| JAK inhibitors (JAK1+JAK3+TYK2) | MI | | | | 0.564 | 36.376 | |
| JAK inhibitors (JAK1+JAK3+TYK2) | MVP | | | | 0.582 | 28.973 | |

Abbreviations: AF, atrial fibrillation; CAD, coronary artery disease; MI, myocardial infarction; HF, heart failure; IS, ischemic stroke; HTN, hypertension; TC, total cholesterol; HDL-C, high density lipoprotein cholesterol; LDL-C, low-density lipoprotein cholesterol; TG, triglycerides; AS, aortic stenosis.
