# Supplementary material for: Association of Disease-Modifying Antirheumatic Drugs (DMARDs) with Cardiovascular Diseases: Evidence from a Drug Target Mendelian Randomization Study
Source: Glob Heart. 2026 Feb 26;21(1):15. doi: 10.5334/gh.1526 (PMC12947826; doi:10.5334/gh.1526)
Supplement: Supplementary File 2. — Figure S1–S15. [file gh-21-1-1526-s2.pdf]

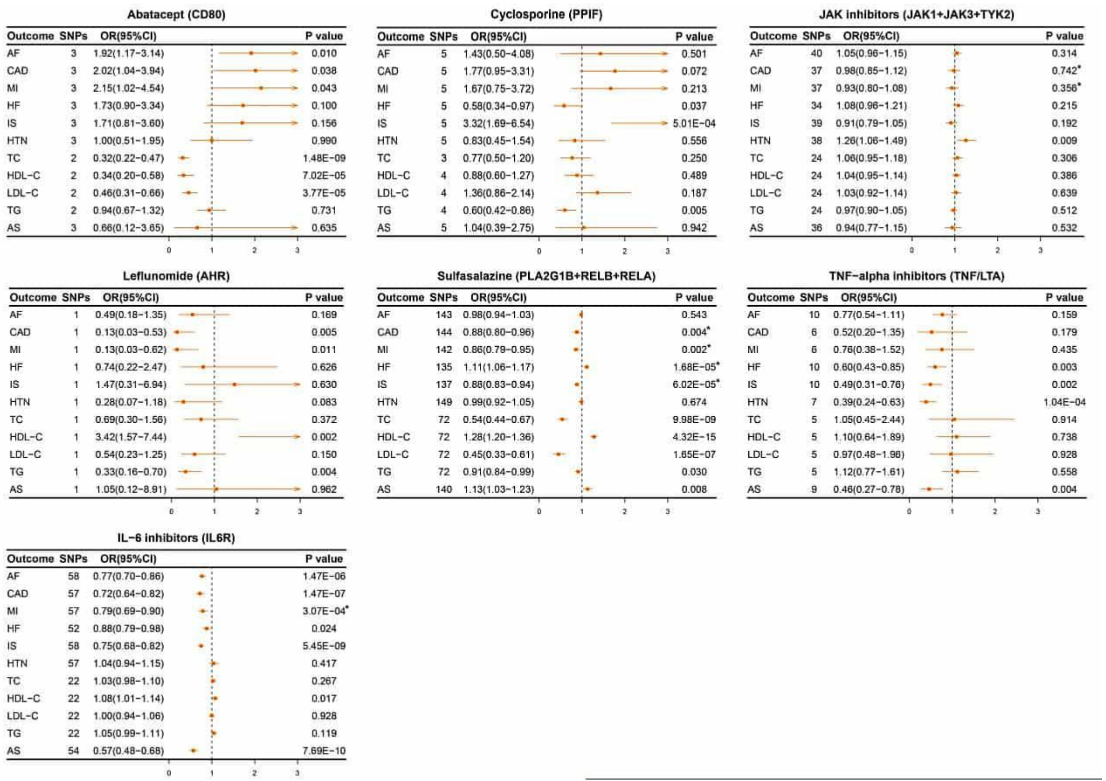

Figure S1. Forest plots of the effects of combined DMARD drug targets on cardiovascular diseases. * indicates P < 0.05 in the MR-Egger intercept test. Abbreviations: AF, atrial fibrillation; CAD, coronary artery disease; MI, myocardial infarction; HF, heart failure; IS, ischemic stroke; HTN, hypertension; TC, total cholesterol; HDL-C, high density lipoprotein cholesterol; LDL-C, low-density lipoprotein cholesterol; TG, triglycerides; AS, aortic stenosis.

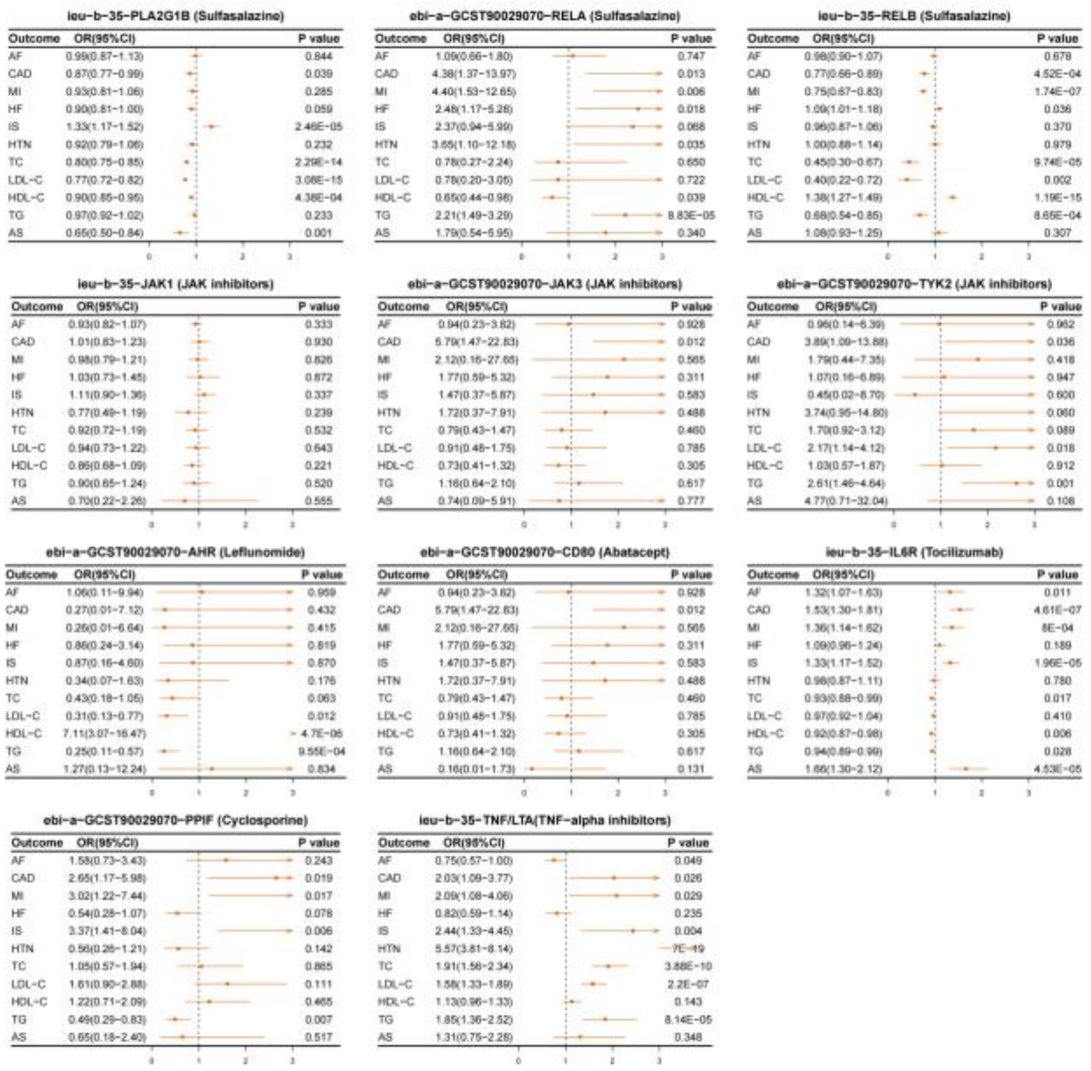

Figure S2. Forest plots of the effects of the DMARD drug targets on CVD outcomes in replication analysis. Abbreviations: AF, atrial fibrillation; CAD, coronary artery disease; MI, myocardial infarction; HF, heart failure; IS, ischemic stroke; HTN, hypertension; TC, total cholesterol; HDL-C, high density lipoprotein cholesterol; LDL-C, low-density lipoprotein cholesterol; TG, triglycerides; AS, aortic stenosis.

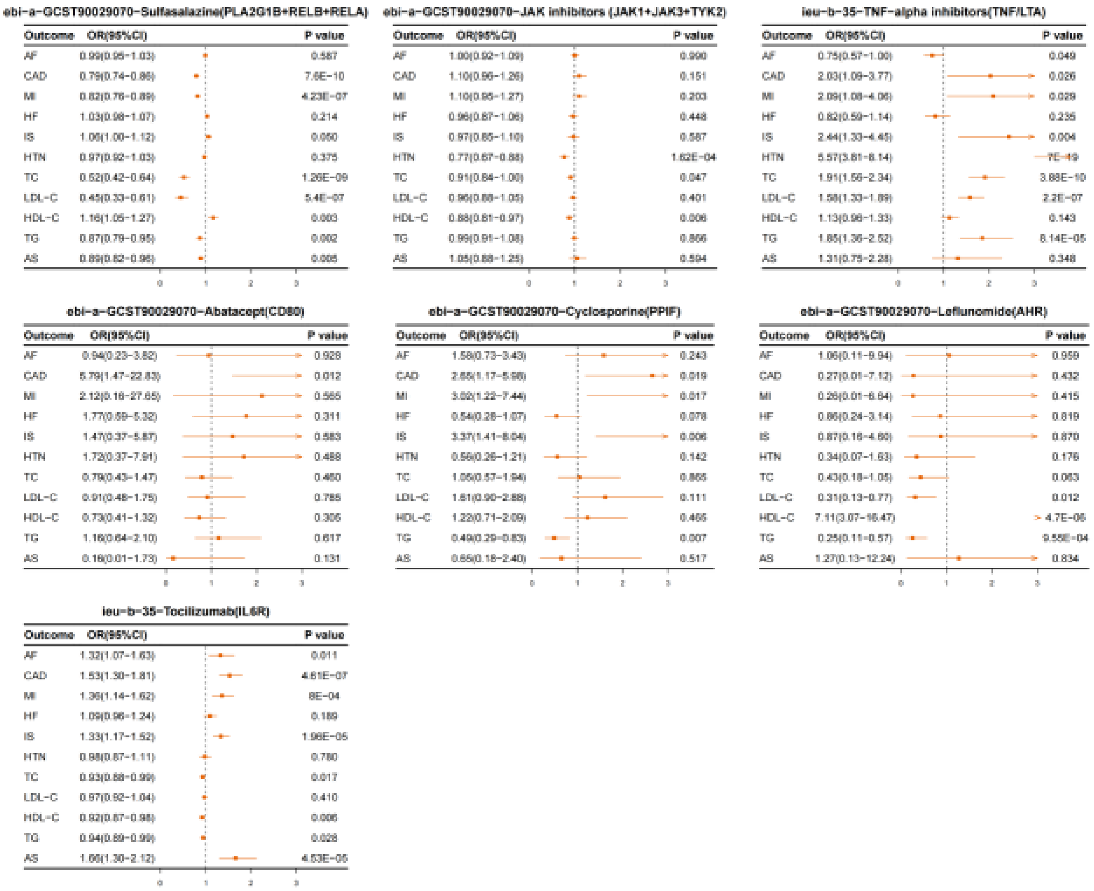

Figure S3. Forest plots of the effects of the combined DMARD drug targets on CVD outcomes in replication analysis. Abbreviations: AF, atrial fibrillation; CAD, coronary artery disease; MI, myocardial infarction; HF, heart failure; IS, ischemic stroke; HTN, hypertension; TC, total cholesterol; HDL-C, high density lipoprotein cholesterol; LDL-C, low-density lipoprotein cholesterol; TG, triglycerides; AS, aortic stenosis.

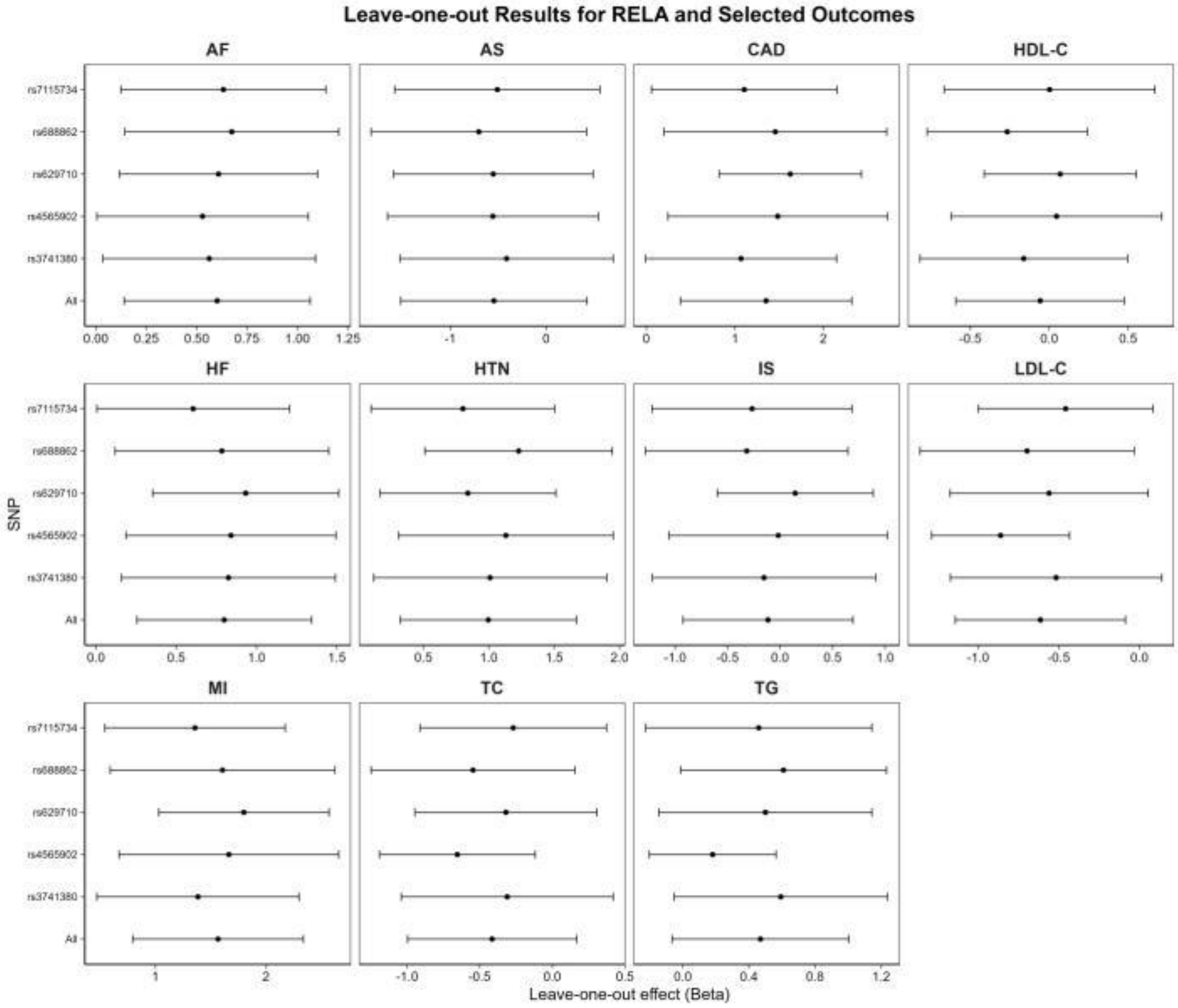

Figure S4. Leave-one-out analyses for RELA across 11 cardiovascular outcomes. The results indicate the presence of potentially influential SNPs driving the causal association of RELA with CAD and LDL-C, and these findings need to be interpreted with caution. Abbreviations: AF, atrial fibrillation; CAD, coronary artery disease; MI, myocardial infarction; HF, heart failure; IS, ischemic stroke; HTN, hypertension; TC, total cholesterol; HDL-C, high density lipoprotein cholesterol; LDL-C, low-density lipoprotein cholesterol; TG, triglycerides; AS, aortic stenosis.

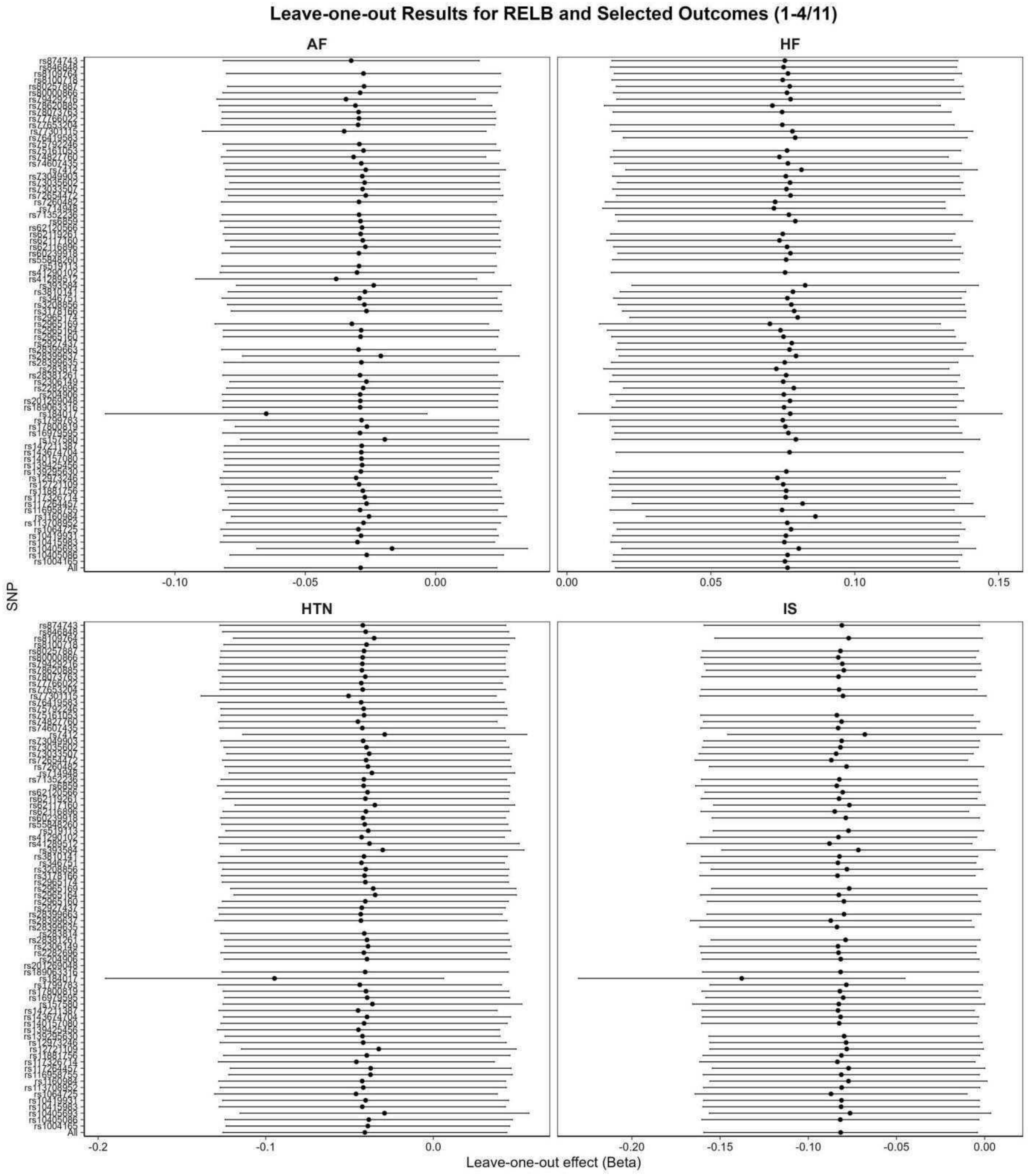

Figure S5. Leave-one-out analyses for RELB (Outcomes 1-4 of 11). This figure, together with Figures S6 and S7, presents the leave-one-out analysis for RELB across 11 outcomes. The results indicate the presence of potentially influential SNPs driving the causal association between RELB and IS, and this specific finding needs to be interpreted with caution. Abbreviations: AF, atrial fibrillation; HF, heart failure; IS, ischemic stroke; HTN, hypertension.

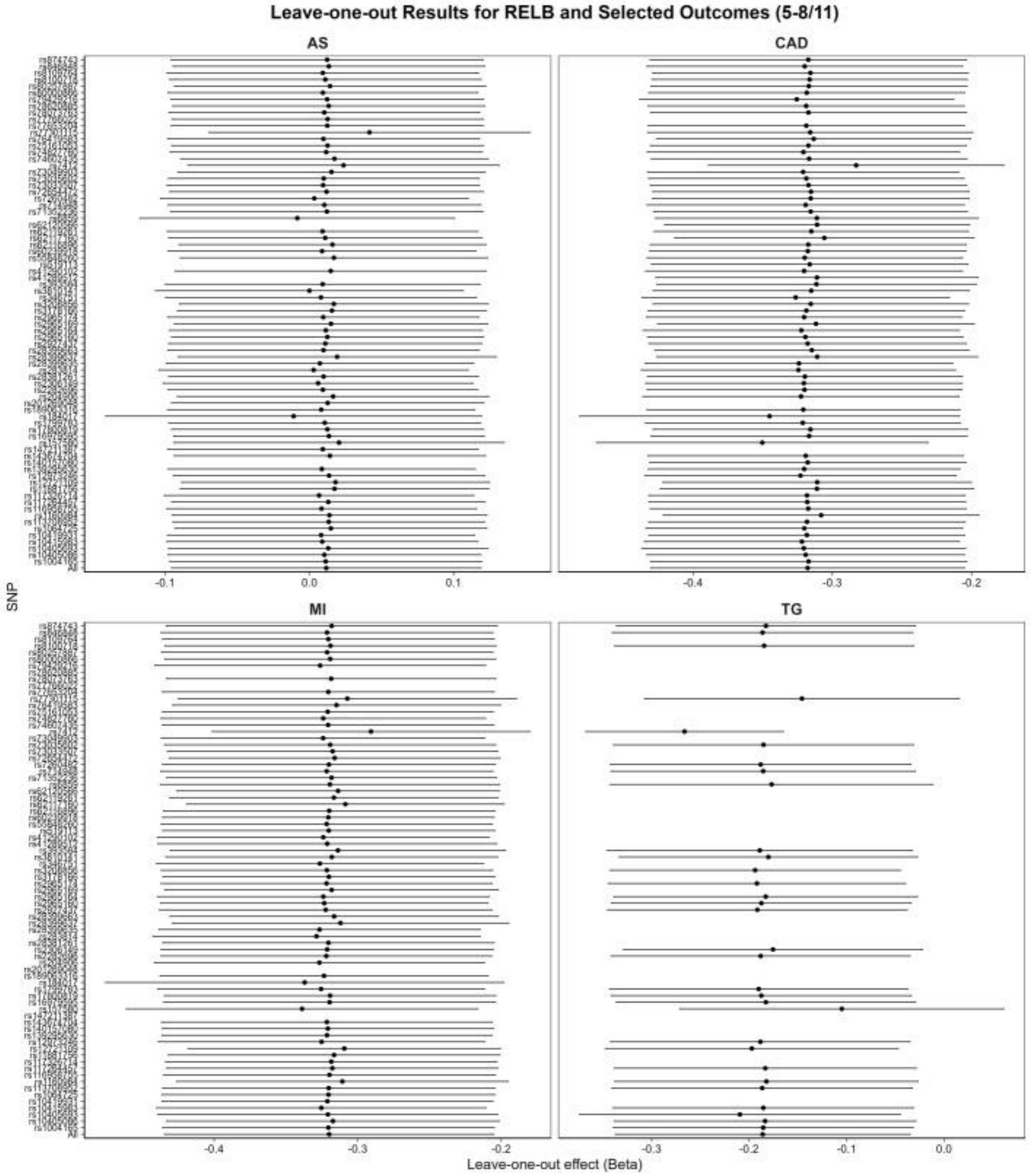

Figure S6. Leave-one-out analyses for RELB (Outcomes 5-8 of 11). This figure, together with Figures S5 and S7, presents the leave-one-out analysis for RELB across 11 outcomes. The results indicate the presence of potentially influential SNPs driving the causal association between RELB and TG, and this specific finding needs to be interpreted with caution. Abbreviations: CAD, coronary artery disease; MI, myocardial infarction; TG, triglycerides; AS, aortic stenosis.

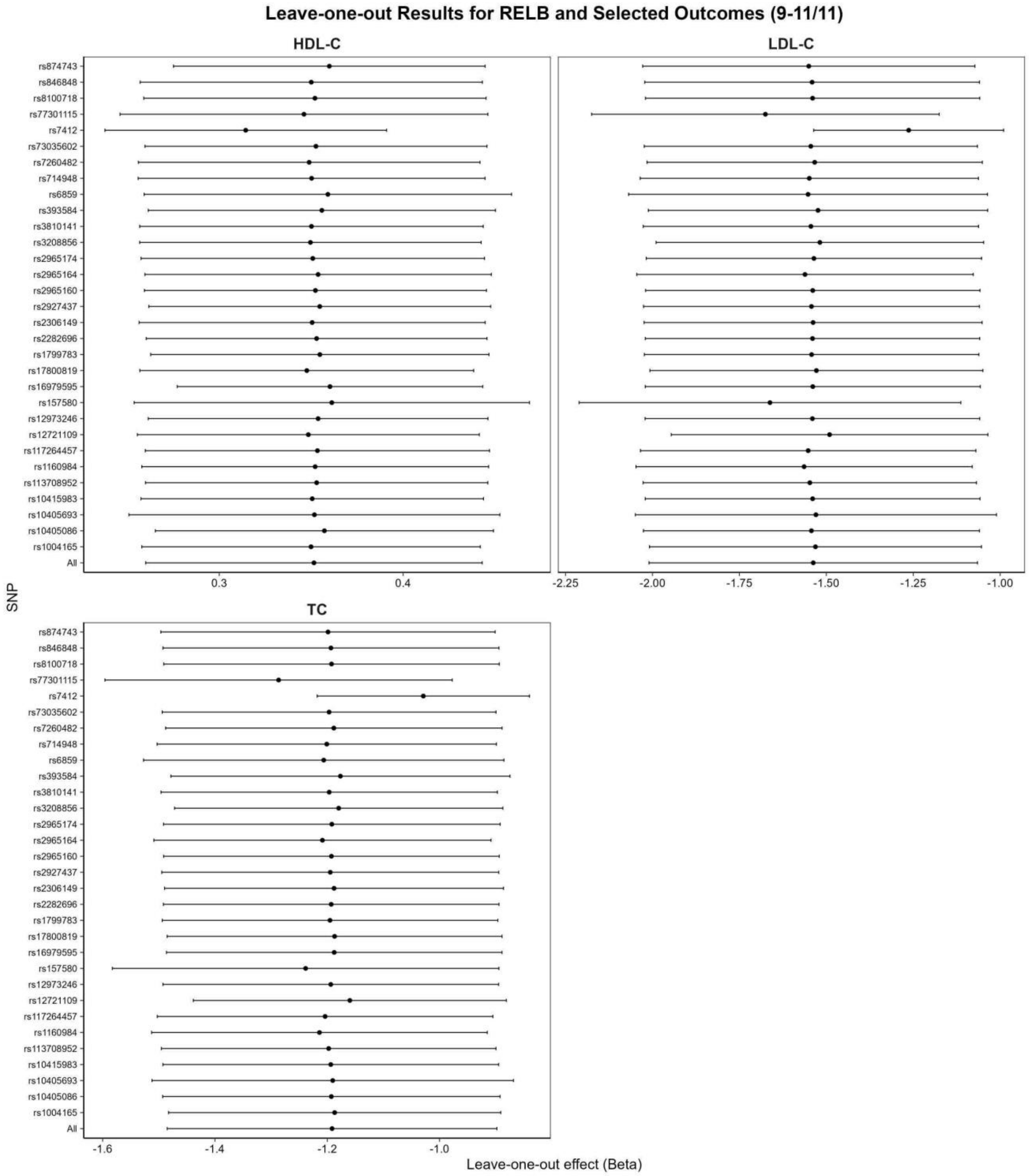

Figure S7. Leave-one-out analyses for RELB (Outcomes 9-11 of 11). This figure, together with Figures S5 and S6, presents the leave-one-out analysis for RELB across 11 outcomes. Abbreviations: TC, total cholesterol; HDL-C, high density lipoprotein cholesterol; LDL-C, low-density lipoprotein cholesterol.

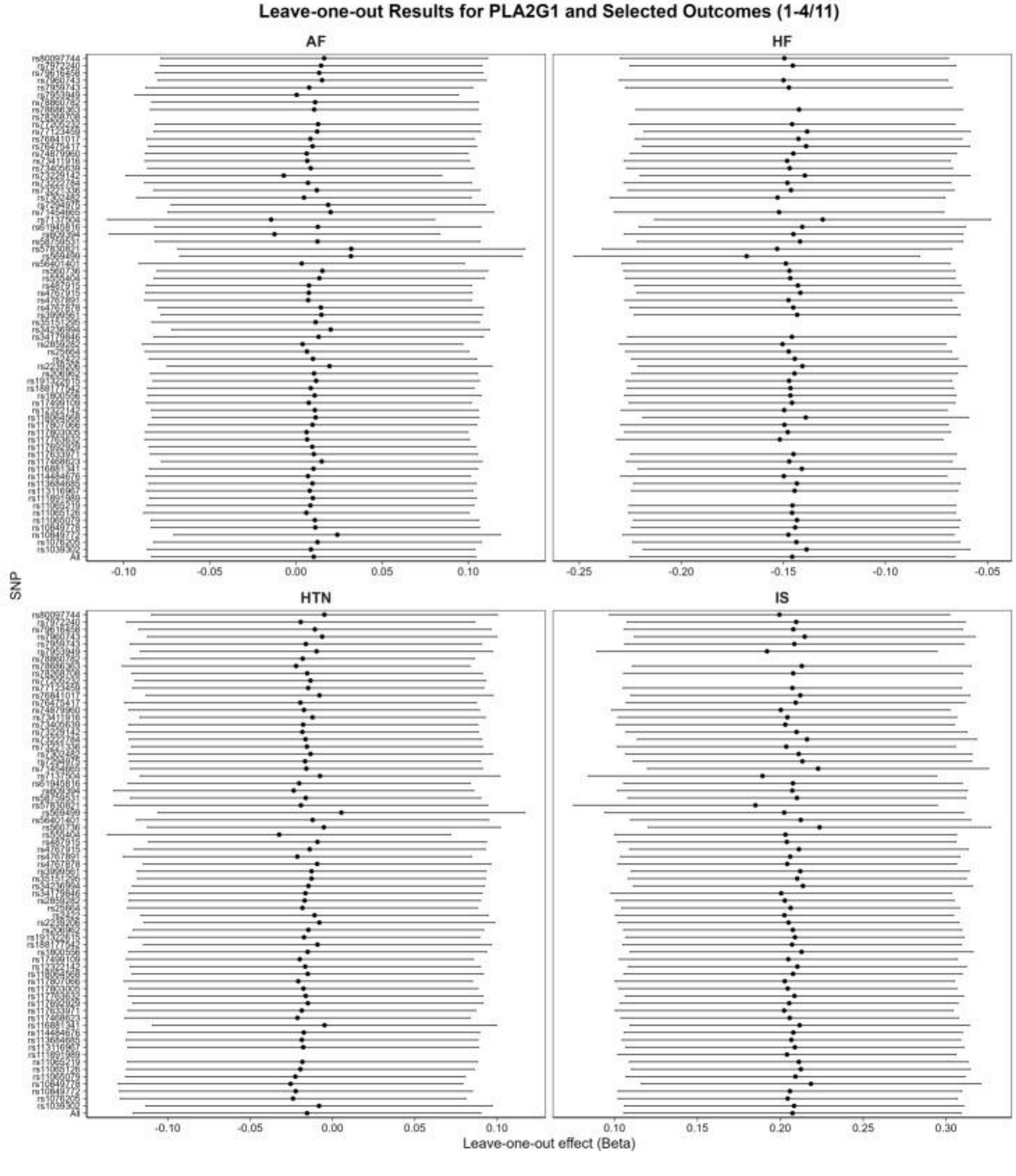

Figure S8. Leave-one-out analyses for PLA2G1 (Outcomes 1-4 of 11). This figure, together with Figures S9 and S10, presents the leave-one-out analysis for PLA2G1 across 11 outcomes. Abbreviations: AF, atrial fibrillation; HF, heart failure; IS, ischemic stroke; HTN, hypertension.

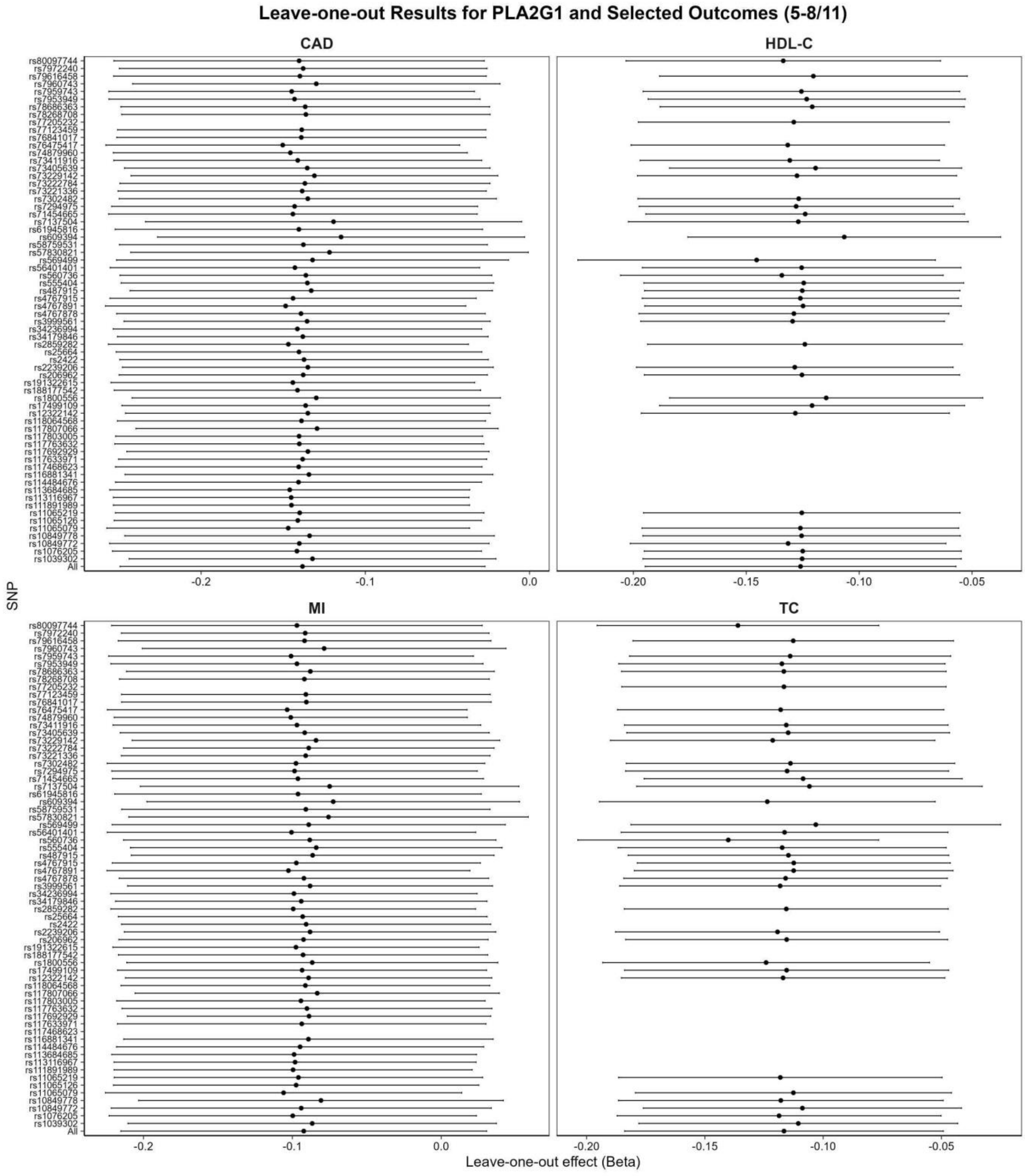

Figure S9. Leave-one-out analyses for PLA2G1 (Outcomes 5-8 of 11). This figure, together with Figures S8 and S10, presents the leave-one-out analysis for PLA2G1 across 11 outcomes. Abbreviations: CAD, coronary artery disease; MI, myocardial infarction; TC, total cholesterol; HDL-C, high density lipoprotein cholesterol.

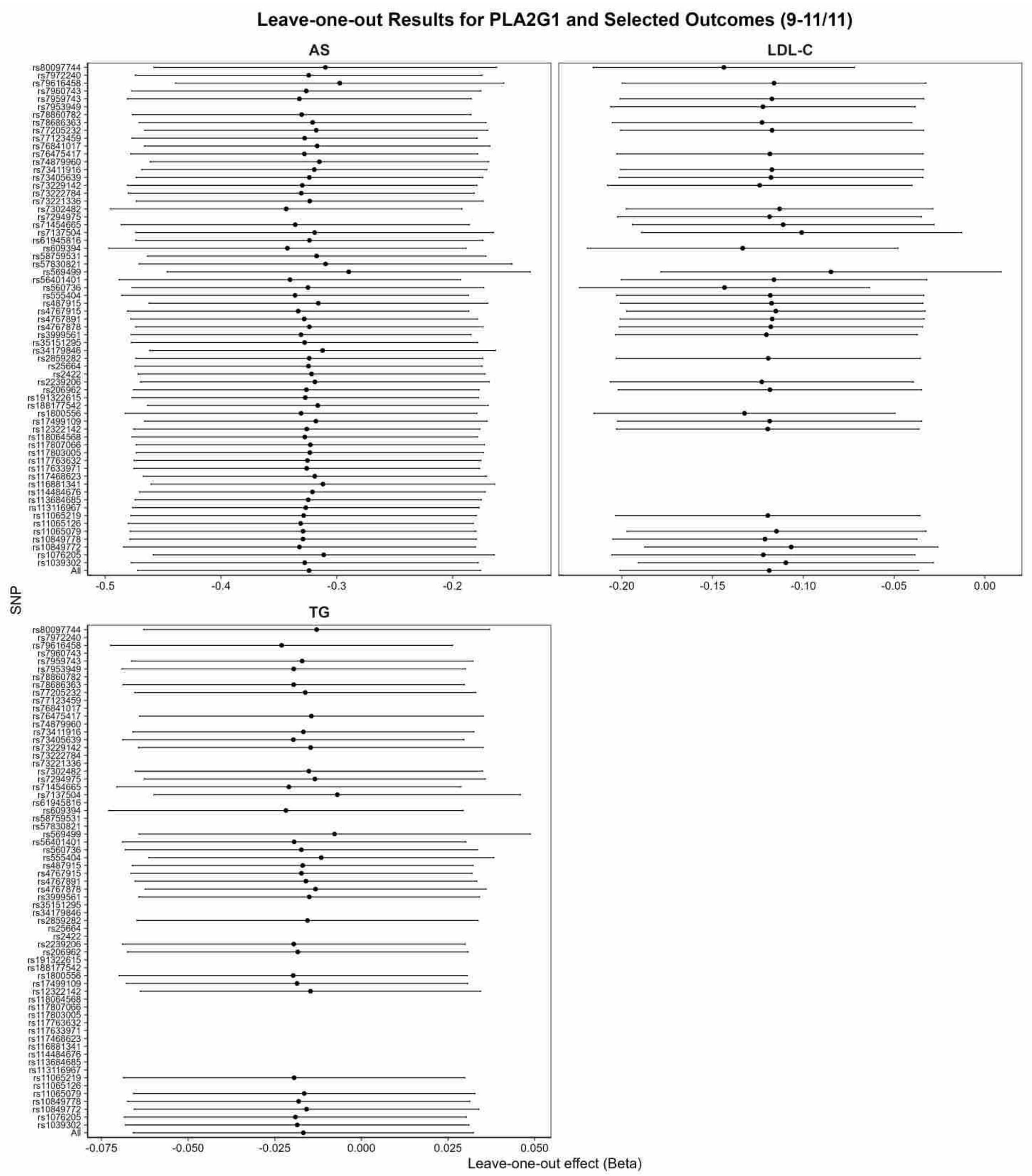

Figure S10. Leave-one-out analyses for PLA2G1 (Outcomes 9-11 of 11). This figure, together with Figures S8 and S9, presents the leave-one-out analysis for PLA2G1 across 11 outcomes. The results indicate the presence of potentially influential SNPs driving the causal association between PLA2G1B and LDL-C, and this specific finding needs to be interpreted with caution. Abbreviations: LDL-C, low-density lipoprotein cholesterol; TG, triglycerides; AS, aortic stenosis.

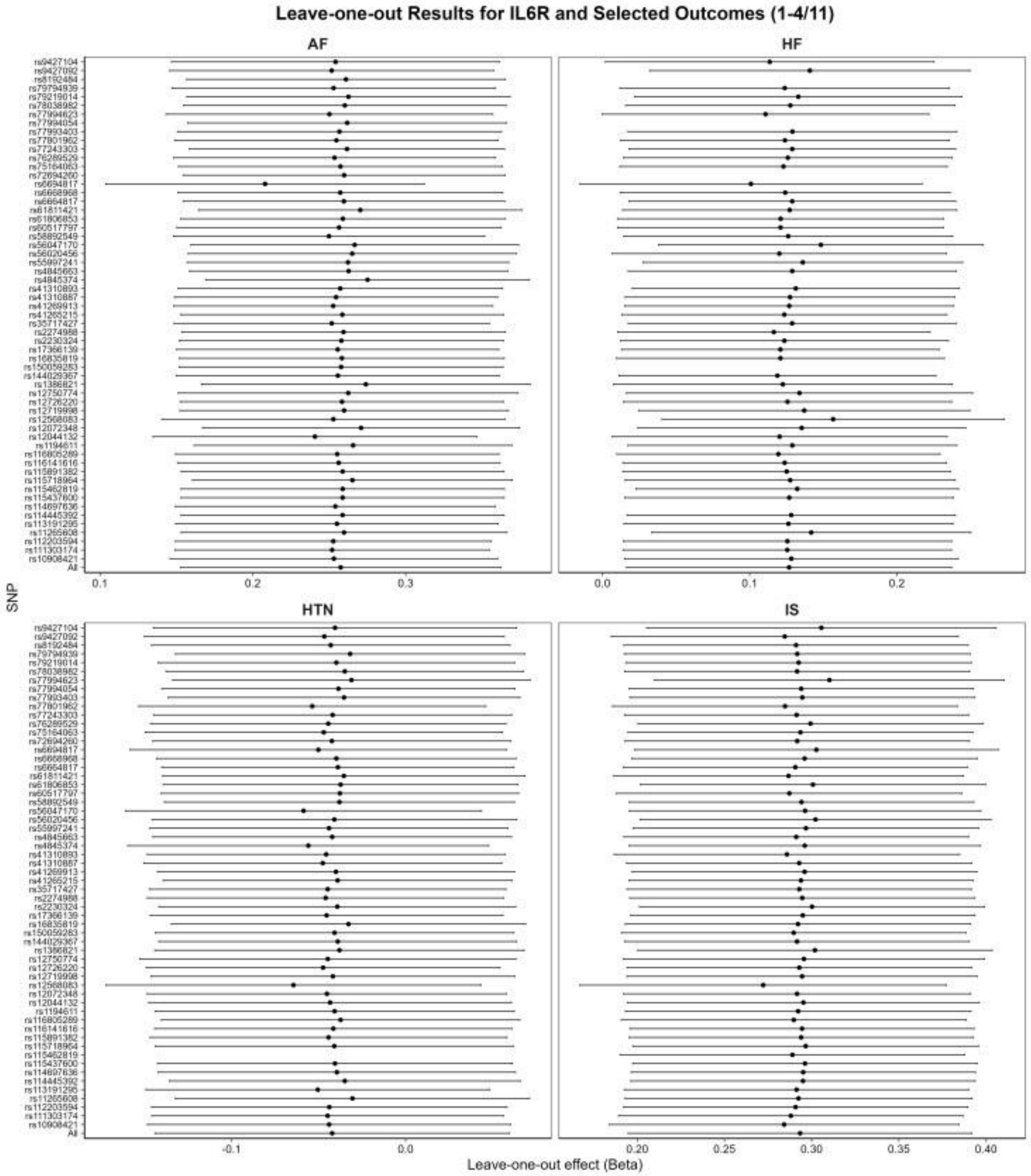

Figure S11. Leave-one-out analyses for IL6R (Outcomes 1-4 of 11). This figure, together with Figures S12 and S13, presents the leave-one-out analysis for IL6R across 11 outcomes. The results indicate the presence of potentially influential SNPs driving the causal association between IL6R and HF, and this specific finding needs to be interpreted with caution. Abbreviations: AF, atrial fibrillation; HF, heart failure; IS, ischemic stroke; HTN, hypertension.

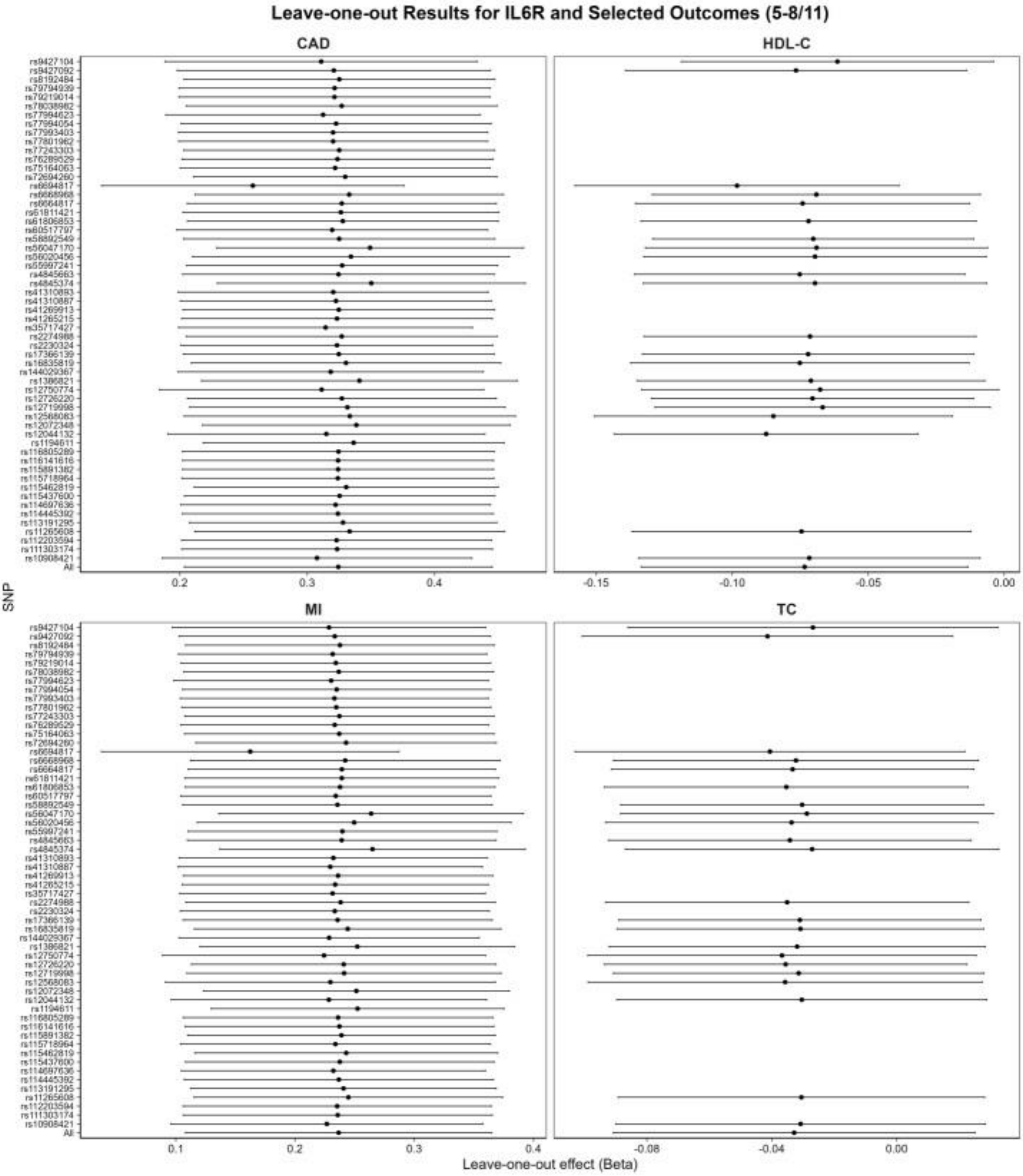

Figure S12. Leave-one-out analyses for IL6R (Outcomes 5-8 of 11). This figure, together with Figures S11 and S13, presents the leave-one-out analysis for IL6R across 11 outcomes. Abbreviations: CAD, coronary artery disease; MI, myocardial infarction; TC, total cholesterol; HDL-C, high density lipoprotein cholesterol.

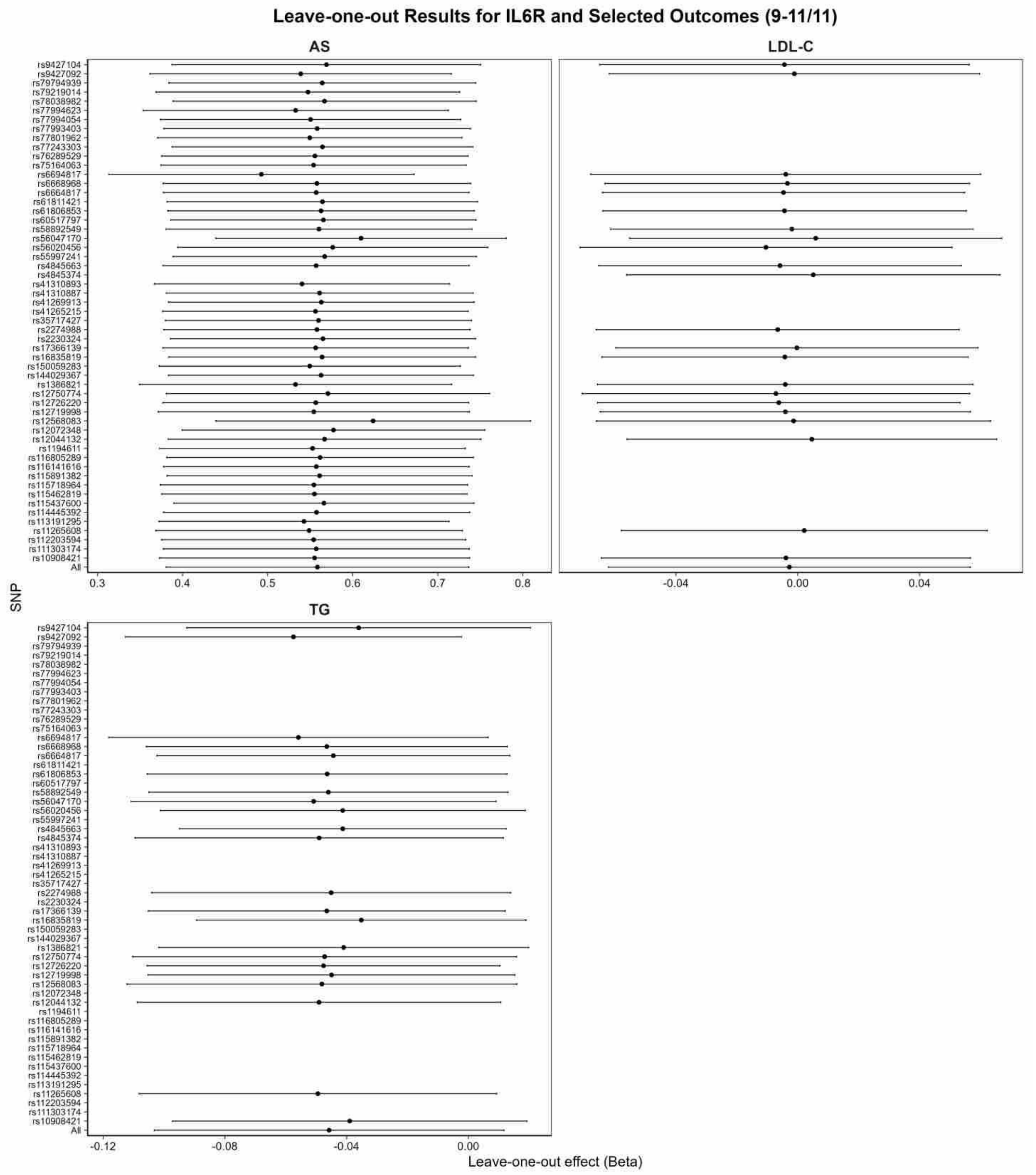

Figure S13. Leave-one-out analyses for IL6R (Outcomes 9-11 of 11). This figure, together with Figures S11 and S12, presents the leave-one-out analysis for IL6R across 11 outcomes. Abbreviations: LDL-C, low-density lipoprotein cholesterol; TG, triglycerides; AS, aortic stenosis.

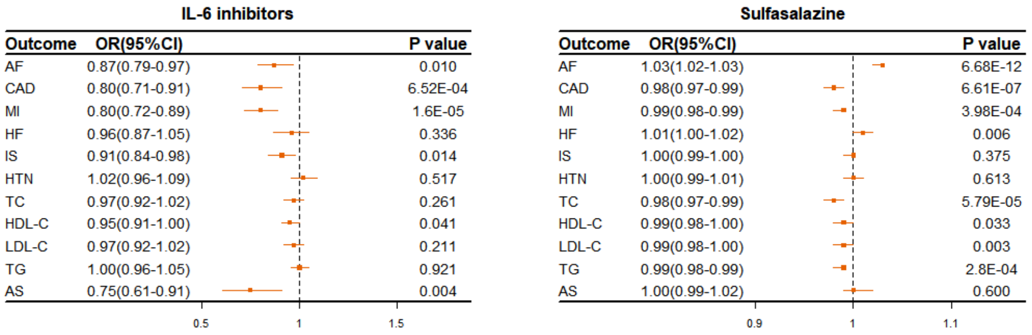

Figure S14. Forest plots of MR estimates for IL-6 inhibitors and sulfasalazine on cardiovascular outcomes using cis-eQTL-based instruments.

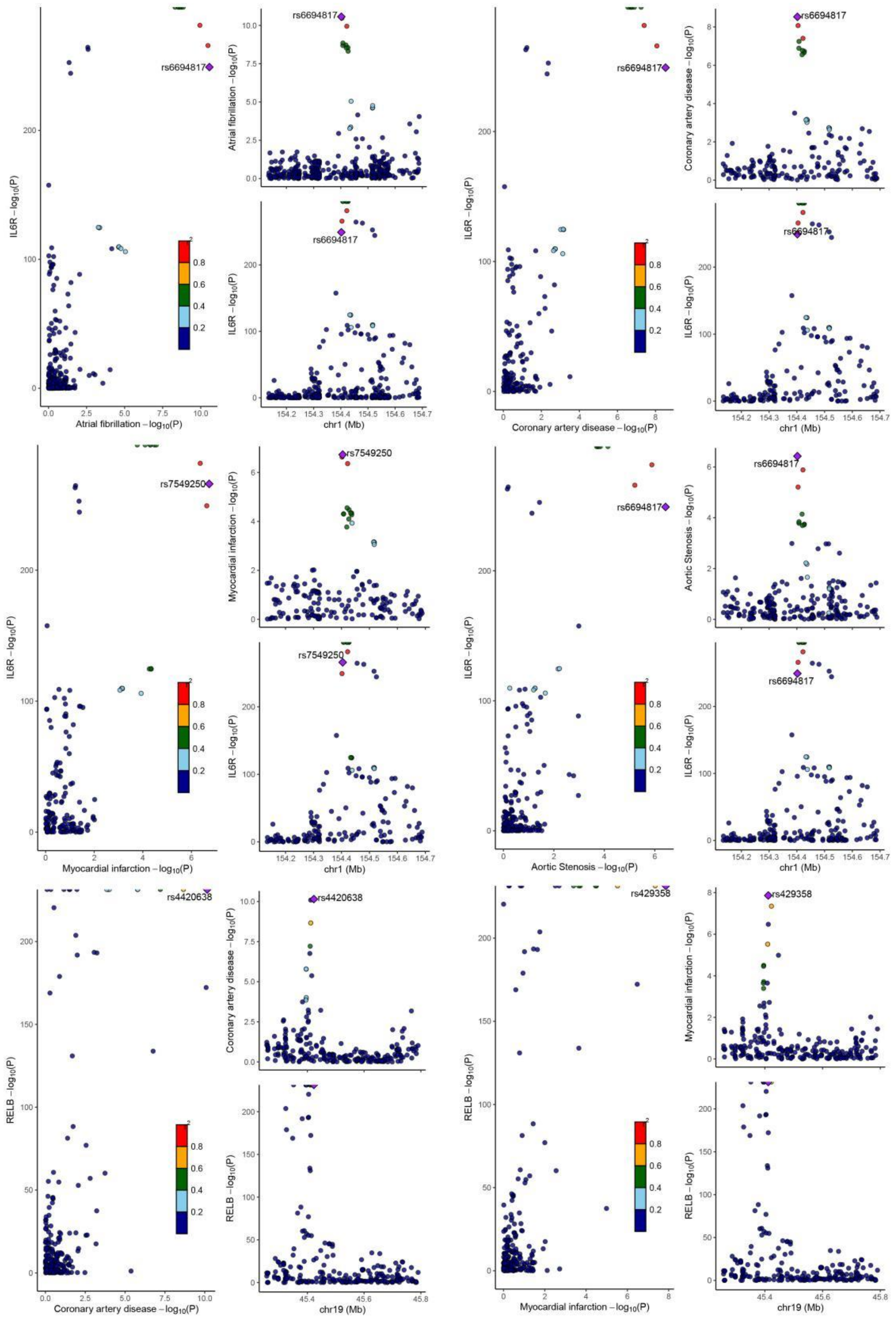

Figure S15. Regional colocalization plots of target genes with cardiovascular disease outcomes. Only results with strong evidence of colocalization (PPH4 > 0.8) are shown.
